# Reassigning CI chondrite parent bodies based on reflectance spectroscopy of samples from carbonaceous asteroid Ryugu and meteorites

**DOI:** 10.1126/sciadv.adi3789

**Published:** 2023-12-06

**Authors:** Kana Amano, Moe Matsuoka, Tomoki Nakamura, Eiichi Kagawa, Yuri Fujioka, Sandra M. Potin, Takahiro Hiroi, Eri Tatsumi, Ralph E. Milliken, Eric Quirico, Pierre Beck, Rosario Brunetto, Masayuki Uesugi, Yoshio Takahashi, Takahiro Kawai, Shohei Yamashita, Yuma Enokido, Taiga Wada, Yoshihiro Furukawa, Michael E. Zolensky, Driss Takir, Deborah L. Domingue, Camilo Jaramillo-Correa, Faith Vilas, Amanda R. Hendrix, Mizuha Kikuiri, Tomoyo Morita, Hisayoshi Yurimoto, Takaaki Noguchi, Ryuji Okazaki, Hikaru Yabuta, Hiroshi Naraoka, Kanako Sakamoto, Shogo Tachibana, Toru Yada, Masahiro Nishimura, Aiko Nakato, Akiko Miyazaki, Kasumi Yogata, Masanao Abe, Tatsuaki Okada, Tomohiro Usui, Makoto Yoshikawa, Takanao Saiki, Satoshi Tanaka, Fuyuto Terui, Satoru Nakazawa, Sei-ichiro Watanabe, Yuichi Tsuda

**Affiliations:** ^1^ Department of Earth Science, Tohoku University, Sendai 980-8578, Japan.; ^2^ Geological Survey of Japan, National Institute of Advanced Industrial Science and Technology (AIST), Tsukuba 305-8567, Japan.; ^3^ Faculty of Aerospace Engineering, Delft University of Technology, 2629 HS Delft, Netherlands.; ^4^ Department of Earth, Environmental, and Planetary Sciences, Brown University, Providence, RI 02912, USA.; ^5^ Institute of Space and Astronautical Science (ISAS), Japan Aerospace Exploration Agency (JAXA), Sagamihara 252-5210, Japan.; ^6^ Institut de Planétologie et d’Astrophysique de Grenoble (IPAG), CNRS, Université Grenoble Alpes, Grenoble 38000, France.; ^7^ Institut d’Astrophysique Spatiale, Université Paris-Saclay, CNRS, Orsay 91405, France.; ^8^ Scattering and Imaging Division, Japan Synchrotron Radiation Research Institute, Sayo 679-5198, Japan.; ^9^ Department of Earth and Planetary Science, The University of Tokyo, Tokyo 113-0033, Japan.; ^10^ Isotope Science Center, The University of Tokyo, Tokyo 113-0032, Japan.; ^11^ Department of Materials Structure Science, The Graduate University for Advanced Studies (SOKENDAI), Tsukuba 305-0801, Japan.; ^12^ Institute of Materials Structure Science, High-Energy Accelerator Research Organization, Tsukuba 305-0801, Japan.; ^13^ NASA Johnson Space Center, Houston, TX 77058, USA.; ^14^ Jacobs, NASA Johnson Space Center, Houston, TX 77058, USA.; ^15^ Planetary Science Institute, Tucson, AZ 85179, USA.; ^16^ The Pennsylvania State University, University Park, PA 16802, USA.; ^17^ Department of Earth and Planetary Sciences, Hokkaido University, Sapporo 060-0810, Japan.; ^18^ Division of Earth and Planetary Sciences, Kyoto University, Kyoto 606-8502, Japan.; ^19^ Department of Earth and Planetary Sciences, Kyushu University, Fukuoka 819-0395, Japan.; ^20^ Department of Earth and Planetary Systems Science, Hiroshima University, Higashi-Hiroshima 739-8526, Japan.; ^21^ Department of Mechanical Engineering, Kanagawa Institute of Technology, Atsugi 243-0292, Japan.; ^22^ Department of Earth and Environmental Sciences, Nagoya University, Nagoya 464-8601, Japan.

## Abstract

The carbonaceous asteroid Ryugu has been explored by the Hayabusa2 spacecraft to elucidate the actual nature of hydrous asteroids. Laboratory analyses revealed that the samples from Ryugu are comparable to unheated CI carbonaceous chondrites; however, reflectance spectra of Ryugu samples and CIs do not coincide. Here, we demonstrate that Ryugu sample spectra are reproduced by heating Orgueil CI chondrite at 300°C under reducing conditions, which caused dehydration of terrestrial weathering products and reduction of iron in phyllosilicates. Terrestrial weathering of CIs accounts for the spectral differences between Ryugu sample and CIs, which is more severe than space weathering that likely explains those between asteroid Ryugu and the collected samples. Previous assignments of CI chondrite parent bodies, i.e., chemically most primitive objects in the solar system, are based on the spectra of CI chondrites. This study indicates that actual spectra of CI parent bodies are much darker and flatter at ultraviolet to visible wavelengths than the spectra of CI chondrites.

## Teaser

The samples from the carbonaceous asteroid Ryugu demonstrate actual reflectance spectra of parent bodies of primitive meteorites.

## INTRODUCTION

Carbonaceous asteroids are dark bodies that can contain hydrated minerals and organic compounds and that likely formed beyond the water snow line [e.g., (*1*)]. Spectroscopic observations of hydrous asteroids have been conducted to investigate the compositional distribution in the solar system [e.g., (*2*)]. Visible–near-infrared (Vis-NIR) reflectance spectroscopy remains the primary technique by which insights from laboratory analyses of meteorites are linked to asteroids, via ground- and space-based instruments, but such observations typically provide only broad spectral properties. The study of microscopic properties of asteroids is crucial to understand their composition better. In this context, detailed laboratory studies of carbonaceous chondrite meteorites, believed to originate from C-complex asteroids, have revealed the likely mineralogical and chemical properties of these small bodies. However, meteorites have already lost the direct connection to their parent bodies, and most primitive meteorites have been chemically and/or mineralogically modified by terrestrial alteration (*3*). This has resulted in uncertainties in the extent to which laboratory spectra of carbonaceous chondrites can be reliably used to infer the composition of hydrous asteroids.

To understand the true composition of C-type asteroids, the spacecraft Hayabusa2 returned 5.4 g of samples from the near-Earth C-type asteroid (162173) Ryugu (*4*). The spacecraft performed close-up observations of the asteroid (*5*) with the onboard multiband camera for visible wavelengths, telescopic optical navigation camera (ONC-T) (*6*, *7*), reflectance spectroscopy with the near-infrared spectrometer (NIRS3) (*8*, *9*), and two landing operations (*10*, *11*). At a global scale, the reflectance properties of Ryugu are quite homogeneous and are characterized by ~2% visible reflectance and an absorption feature at 2.72 μm with 10% depth attributed to Mg-rich hydrated silicates (*7*, *8*). The collected samples show Vis-NIR reflectance spectra roughly consistent with ONC-T and NIRS3 observations (*4*, *12*, *13*), although some differences potentially due to space weathering exist (*14*). The present study reports the spectral variation of millimeter-sized Ryugu samples in the range of ultraviolet (UV), visible, NIR, and mid-infrared (MIR) (0.2 to 18 μm) measured without atmospheric exposure to avoid terrestrial alteration. We evaluate these spectra in the context of the independently determined Ryugu sample mineralogy (*13*, *15*) and discuss the similarities and differences compared to analogous carbonaceous chondrites.

Reflectance spectra of Ryugu acquired by the spacecraft were pointed out to be most similar to laboratory spectra of heated and partially dehydrated carbonaceous chondrites, particularly experimentally heated CI chondrites (*7*, *8*). However, the mineralogical and chemical properties of samples returned from Ryugu are most similar to those of unheated CI chondrites (*13*, *16*). The Vis-NIR reflectance spectra of Ryugu samples are much darker and flatter than those of unheated CI chondrites (*4*, *13*). Thus, to identify the causes of the spectral similarities between Ryugu and heated CI chondrites and the spectral differences between Ryugu samples and unheated CI chondrites, we performed experimental heating of Orgueil CI chondrite and compared the spectral properties to those of Ryugu samples. We investigated spectral and mineralogical changes depending on the heating temperature and duration, which allows us to examine the extent of the heating that Ryugu might have experienced. The experimental heating of CI chondrite samples was performed at 300° to 900°C under reducing conditions. The previous studies that performed heating experiments using hydrous chondrites revealed marked changes in mineral composition (*17*–*19*), organic composition (*20*), and reflectance spectra [e.g., (*21*, *22*)] with heating. Furthermore, naturally heated CI and CM carbonaceous chondrites, or CY chondrites, have been characterized in terms of mineral composition (*23*–*25*), organic composition (*26*), water contents (*25*, *27*), and infrared (IR) spectroscopy (*25*, *28*–*31*). However, atmospheric alteration such as water adsorption on phyllosilicates and oxidation of minerals and organics occurs in both experimentally and naturally heated meteorites [e.g., (*32*)], which makes it difficult to compare the properties of the heated samples to asteroidal materials heated in space. In particular, for reflectance spectra of heated carbonaceous chondrites, the absorption band depth at 2.7 to 2.8 μm in wavelength due to −OH stretching in hydrated silicates can be masked by a broad absorption feature at ~2.9 μm due to adsorbed water (*32*–*35*), whereas the absorption band at 2.7 to 2.8 μm can be an indicator of the degree of hydration and dehydration (*30*–*32*, *34*). Thus, reflectance spectra of the heated samples were measured before exposure to air to avoid effects of terrestrial alteration after heating.

## RESULTS

### Visible spectral diversity of Ryugu samples at visible wavelengths

**Fig. 1.**
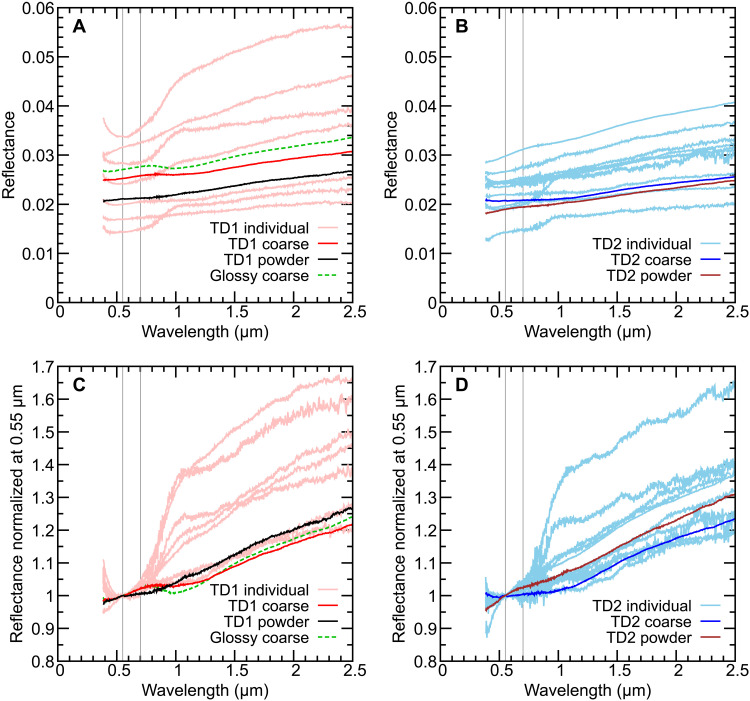
Vis-NIR reflectance spectra of Ryugu samples. (**A**) Vis-NIR spectra of TD1 samples, (**B**) Vis-NIR spectra of TD2 samples, (**C**) TD1 sample spectra normalized at 0.55 μm, and (**D**) TD2 sample spectra normalized at 0.55 μm. The vertical gray lines indicate 0.55 and 0.70 μm. Spectral data of TD1 coarse, TD1 powder, TD2 coarse, and TD2 powder are reported in (*13*). Ryugu samples exhibit dark and red-sloped Vis-NIR reflectance spectra. Low signal levels at ~0.38 to 0.5 μm in wavelength may affect the spectral data, particularly resulting in an abrupt change in slope toward the UV region.

Reflectance spectra for 14 different millimeter-sized Ryugu grains were measured (see Materials and Methods for the sample IDs) at incidence, emergence, and phase angles of 30°, 0°, and 30° in the principal plane, respectively, within an air-tight cell for the purpose of not exposing samples to air. Incident beam size was modified from ~1 to 5 mm in diameter depending on sample size. Ryugu grains collected at the first and second touchdown sites were stored in chambers A and C, respectively, and are named accordingly. Ryugu individual grains exhibit ~2 to 4% of reflectance at v-band (0.55 μm in wavelength) and red-sloped spectra at Vis-NIR wavelengths with 0.03 to 0.44 μm^−1^ of spectral slope from 0.48 to 0.86 μm, i.e., b-x slope (Fig. 1). Millimeter-sized grains collected from the first touchdown site (TD1; previously undisturbed surface of asteroid Ryugu) (*10*) were measured individually and are referred to here as the “TD1 individual grains.” Spectra of these grains showed a wide range of v-band reflectance values (Fig. 1A) and spectral slope (Fig. 1C), whereas grains collected at the second touchdown site [TD2; site including materials excavated by small carry-on impactor (SCI) experiment] (*11*, *36*), hereafter referred to as the “TD2 individual grains,” exhibit less variability in these properties (Fig. 1, B and D). The 0.7-μm absorption band commonly attributed to Fe^2+^ + Fe^3+^–bearing phyllosilicates (e.g., cronstedtite and other serpentine minerals) is typically seen in CM2 chondrites [e.g., (*37*)]. However, it was not observed in Ryugu samples. No pronounced features due to anhydrous silicates such as olivine and pyroxene (e.g., absorption bands at 1 and 2 μm) (*37*) are observed in Ryugu samples. Some Ryugu grains (A0064, A0067, C0040, and C0055) were analyzed twice in different sample orientations and are distinguished as settings A and B. They show spectra with different v-band reflectance and spectral slope (fig. S1), suggesting the heterogeneous angular distribution of diffuse reflection from their surfaces.

Individual grains were combined to create bulk aggregates samples of TD1 and TD2, hereafter, referred to as the “TD1 coarse” and “TD2 coarse,” respectively. Spectra of these coarse aggregates are brighter than the spectra of aggregate samples of fine particles (<1 mm of diameter, hereafter, referred to as the “TD1 powder” and “TD2 powder”) (Fig. 1, A and B) (*13*). Some individual grains (three grains from TD1 suite and five grains from TD2 suite) exhibited a glossy and smooth surface and were combined to create a separate aggregate sample (fig. S2). This “glossy coarse” aggregate sample exhibits a higher reflectance value than TD1 and TD2 coarse (Fig. 1A). Reflectance spectra of both coarse and powder samples of TD1 and TD2 are within the spectral range of Ryugu TD1 and TD2 individual samples, respectively, suggesting that spectral data of TD1 and TD2 coarse and powder aggregates samples can be regarded as representative of Ryugu returned samples, in which light reflected from different surface orientations is averaged.

### Spectral comparison among Ryugu samples, chondrite chip samples, and experimentally heated Orgueil powder samples in visible region

**Fig. 2.**
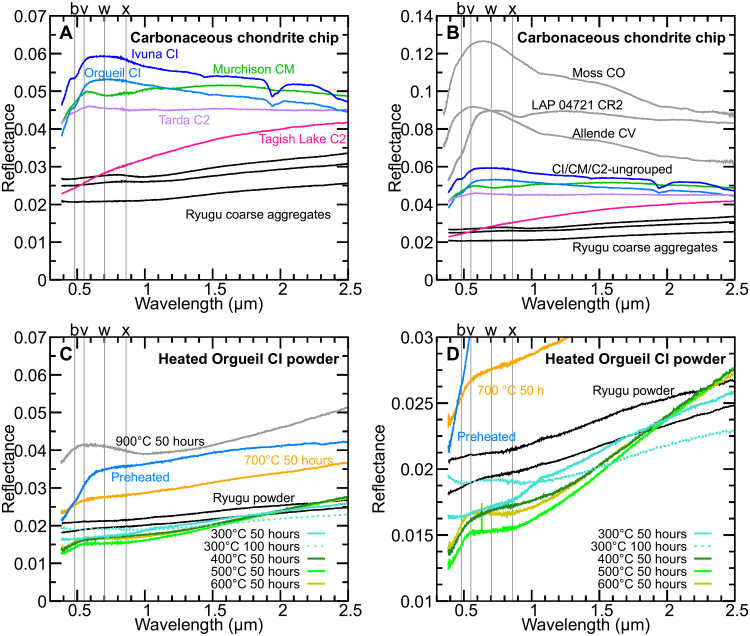
Vis-NIR reflectance spectra of Ryugu and chondrite samples. (**A** and **B**) Ryugu coarse aggregate samples (TD1, TD2, and glossy coarse) and carbonaceous chondrite chip samples (Orgueil CI, Ivuna CI, Tagish Lake C2–ungrouped, Tarda C2–ungrouped, Murchison CM, Allende CV, Moss CO, and LAP 04721 CR2). Spectral data of Allende and Moss chondrites are cited from (*40*). The vertical gray lines indicate 0.48, 0.55, 0.70, and 0.86 μm in wavelength (b-, v-, w-, and x-bands, respectively) (*6*). Ryugu’s Vis-NIR reflectance spectra are darker than hydrated carbonaceous chondrites (A) and anhydrous carbonaceous chondrites (B). (**C** and **D**) Vis-NIR reflectance spectra of Ryugu powder aggregate samples and experimentally heated Orgueil (CI) powder samples. Ryugu’s dark and flat spectra are similar to those of Orgueil powder samples heated at 300° to 600°C.

Ryugu sample spectra have lower reflectance values compared with chip samples of hydrated carbonaceous chondrites falls, such as Orgueil CI, Ivuna CI, Murchison CM, and Tarda C2–ungrouped chondrites, and are more similar to reflectance values of Tagish Lake C2–ungrouped chondrite chips (Fig. 2A). Ryugu TD1 and TD2 coarse aggregate samples show red-sloped spectra without a sharp drop in reflectance at UV wavelengths, i.e., UV drop-off features (Fig. 2A and fig. S1) (*38*). The Tagish Lake spectrum is more red-sloped than those of Ryugu samples (Fig. 2A). The CI, CM, and Tarda chip samples exhibit UV drop-off features. The shoulder-like absorption feature at 0.5 μm observed in Orgueil CI and Ivuna CI, which can be attributable to iron oxides/hydroxides (*39*), is not observed in the Ryugu samples used in this study. Ryugu’s dark and flat reflectance spectra are also not similar to those of the Allende CV and Moss CO meteorite chip samples (Fig. 2B) (*40*). Absorption features due to Fe^2+^ in olivine (1 μm) or pyroxene (1 and 2 μm) (*37*) were not observed in Ryugu sample spectra (Fig. 2B and fig. S1). The absence of those bands in Ryugu spectra precludes a compositional link between Ryugu and anhydrous CV and CO carbonaceous chondrites. In addition, the CR chondrite LAP 04721, which consists primarily of hydrated minerals (mostly serpentine) and magnetite (fig. S3), exhibits spectra that are brighter and bluer than Ryugu grains, as well as an absorption feature at 0.9 μm due to Fe hydroxides, terrestrial weathering products (Fig. 2B) (*41*). This comparison demonstrates that Ryugu samples are spectrally distinct and perhaps unique when compared with spectra of natural (i.e., not experimentally modified) carbonaceous chondrites, including CIs and other samples that are compositionally similar to Ryugu.

To investigate spectral changes of CI chondrites due to heating, Orgueil powder samples with a maximum grain size of 155 μm were heated at 300°, 400°, 500°, 600°, 700°, and 900°C for 50 hours under vacuum (~10^−6^ torr) and reducing conditions (at Iron-Wüstite buffer, using iron powder) (see also the “Mineralogical changes of Orgueil upon experimental heating” section). A sample heated at 300°C for 100 hours was also measured to assess effects of heating duration. Another portion of Orgueil powder was preheated at 150°C, a temperature at which phyllosilicates do not decompose (*17*), for 3 hours in an N_2_ atmosphere to remove adsorbed water. This sample is referred to hereafter as “preheated” sample to distinguish it from the “unheated” sample that was not treated to remove adsorbed water (see Materials and Methods for details). Reflectance spectra of preheated and heated samples were measured before exposure to air using an air-tight cell. Preheated Orgueil powder exhibits ~3.5% reflectance at v-band (Fig. 2C), which is darker than unmodified Orgueil chip (Fig. 2A). Orgueil powder samples heated at 300° to 600°C for 50 hours under reducing conditions exhibit reflectance values of <2% at v-band and shallower UV drop-off features than the preheated sample (Fig. 2, C and D). The starting points of the drop in reflectance toward UV wavelengths of the heated samples were moved to a shorter wavelength than that of preheated sample (Fig. 2C). With the exception of some irregularly bright spectra (possibly due to enhanced specular reflection), reflectance spectra of Ryugu samples are consistent with those of Orgueil powder heated at 300° to 600°C in terms of the low reflectance (~2% at v-band), spectral flatness (low b-x slope), and the absence of strong UV drop-off features (fig. S4, A and B).

### NIR reflectance spectra (2 to 4 μm)

**Fig. 3.**
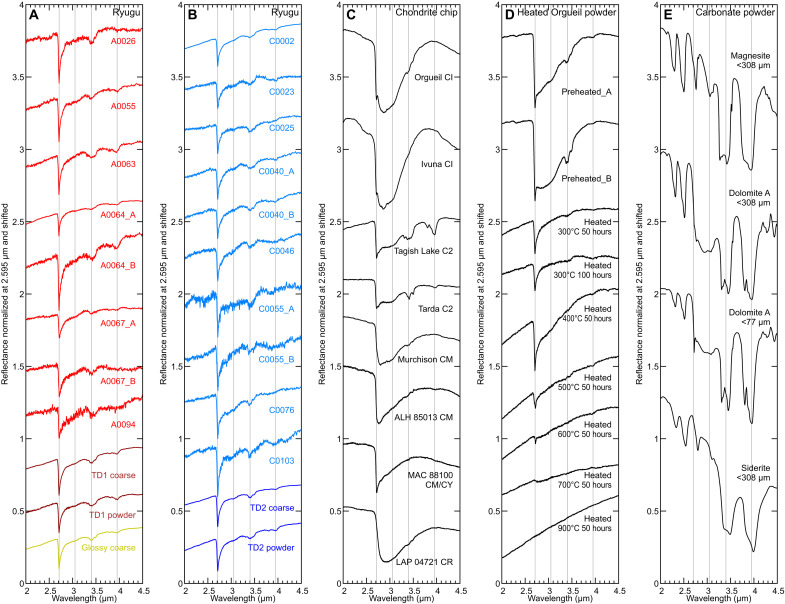
IR (2 to 4 μm) reflectance spectra. (**A** and **B**) Ryugu samples, (**C**) hydrated carbonaceous chondrite chip samples, (**D**) experimentally heated Orgueil CI powder samples without exposure to air after experimental heating, and (**E**) carbonate powder samples. All the spectra are normalized at 2.595 μm and shifted arbitrarily. Gray lines indicate 2.71, 3.05, 3.40, and 3.95 μm.

Previous studies have reported that the maximum absorption position of metal-OH absorption at ~2.7 μm correlates with Fe/Mg ratios of phyllosilicates (*33*). Ryugu samples most commonly have an absorption maximum at 2.71 μm indicative of Mg-rich phyllosilicates (Fig. 3, A and B) (*13*), which is similar to narrow features observed in spectra of Orgueil CI, Ivuna CI, Tagish Lake, and Tarda chondrites (Fig. 3 and fig. S5A). On the other hand, the maximum absorption position of many CM and CR chondrites is located at a longer wavelength than is observed for Ryugu samples, indicating that some phyllosilicates in those samples are richer in Fe (Fig. 3C and fig. S5A) (*33*). The band depth of samples was evaluated on the basis of the spectral deconvolution of the Ryugu sample spectra using the exponentially modified Gaussian (EMG) profile (fig. S6) (*42*) as described in the Supplementary Text. The 2.7-μm band depth of Ryugu samples ranges from 20 to 40%, which is much weaker than natural Orgueil (>50%) (fig. S5A). The 2.7-μm band shapes of Ryugu samples are asymmetric and sharper than those of most carbonaceous chondrites, where the latter commonly exhibit a broad absorption feature with a local minimum near 2.9 μm due to molecular water [e.g., (*43*)]. This suggests that Ryugu samples contain less H_2_O than CI chondrites, being consistent with H_2_O concentration revealed by previous analyses (*13*, *16*). Ryugu sample spectra show a small absorption band at 3.05 to 3.1 μm (Fig. 3, A and B) in agreement with previous spectral analyses performed at Japan Aerospace Exploration Agency (JAXA) curation facility with pristine Ryugu samples (*12*). The 3.1-μm absorption band observed in Ryugu grains in this study is notably weaker than those seen in a particular Ryugu grain reported in Ryugu samples (*12*) and as observed for the dwarf planet Ceres by the DAWN spacecraft (*44*). Even so, the EMG fitting results (*42*) suggest a weak component at 3.05 to 3.07 μm (fig. S6). This feature may be attributed to ammonia-bearing phyllosilicates, salts, or organics [e.g., (*12*, *45*)], which should accompany a stronger absorption feature at ~7 μm (fig. S7) (*46*). We have not yet concluded the cause of the faintness of the 3.1-μm band, and the origin of the band remains elusive in the absence of coupled absorption features.

Preheated and heated Orgueil powder samples show a marked change in the shapes and strength of the 3 μm region as well as the strength of the 2.7-μm absorption band (Fig. 3D). Preheated Orgueil powder samples exhibit a band depth value of >50% for the 2.7-μm OH, 2.9-μm molecular water, and ~3.4-μm organic and carbonate features. The 2.7-μm band depth decreases with increasing temperature until 600°C, and the band disappears at 700°C. This is consistent with x-ray diffraction (XRD) patterns collected from the matrix grains of the heated sample that indicate phyllosilicates completely decomposed at these higher temperatures (see the “Mineralogical changes of Orgueil upon experimental heating” section). The 700°C-heated Orgueil spectrum shows a faint absorption due to a small amount of adsorbed water. The specific positions of the 2.7-μm band of preheated, 300°C-heated, and 400°C-heated Orgueil are located at 2.71 μm, whereas those of 500°C-heated and 600°C-heated samples are shifted to 2.72 μm. Orgueil samples heated at 300°C for 50 or 100 hours are spectroscopically similar to Ryugu samples regarding the 2.7-μm band depth, its wavelength position, the lack of molecular water, and the red-sloped continuum (Fig. 3, A, B, and D). The 3.4-μm band due to carbonates (Fig. 3E) (*47*) and aliphatic compounds (*48*) are stronger in Ryugu samples than in heated Orgueil samples (Fig. 3, A, B, and D).

### IR reflectance spectra (4 to 8 μm)

At IR wavelengths (4 to 8 μm), Ryugu grains have a red spectral slope (fig. S8, A and B), which is similar to the Tagish Lake chip sample (fig. S8C). The chip samples of other carbonaceous chondrites show bluer spectra at both shorter and longer wavelengths than the 2.7-μm absorption band (fig. S8C). Ryugu spectra do not show intense absorption features at ~6 μm, suggesting less molecular water in Ryugu samples than natural carbonaceous chondrites (fig. S8C). The Orgueil samples heated at 300° to 500°C exhibit a shallower absorption at ~6 μm than those of the preheated Orgueil samples (fig. S8D).

The Ryugu IR reflectance spectra are dominated by carbonate features that include absorption bands at ~3.4 and 3.95 μm and peaks due to asymmetric stretching vibration of the CO_3_^2−^ anion at ~6 to 7 μm (fig. S8, A, B, and E) (*47*). Some Ryugu grains (e.g., A0063; fig. S8A) show absorption features at 4.20, 4.58, and/or 5.5 μm, which are additional minor features due to carbonates (*47*), indicating particularly high abundance of carbonates in those grains. The peak positions of those carbonate features in Ryugu spectra suggest that most carbonates in Ryugu samples are dolomite (fig. S8E). The 3.95-μm absorption band due to carbonates is observed in Ryugu, Tagish Lake, and Tarda; however, it is not obvious in Orgueil, Ivuna, or some CM chondrites (fig. S8, A to C). The 5.5-μm absorption band due to carbonates is seen in some Ryugu grains and Tagish Lake; faint in Tarda; and not detected in Orgueil, Ivuna, or CM chondrites. Carbonates also have overtone features at Vis-NIR wavelengths (~1.75, 1.9, 2.0, 2.3, and 2.5 μm) (*47*); however, Ryugu spectra are free from those features (fig. S1, A, B, and E) due to severe masking effects at Vis-NIR wavelengths than at longer wavelengths. Darkening agents in carbonaceous chondrites are still debated but mixing carbon with minerals produces similar effects (fig. S9). The Orgueil samples preheated and heated at 300° to 500°C show spectral features due to carbonates (fig. S8D), whereas these features are absent in the 700°C-heated Orgueil sample, which is in good agreement with the XRD results.

Sulfates and ammoniated salts or ammonium-bearing phyllosilicates have intense absorption at ~4.5 μm (fig. S10A) (*49*) and ~7 μm (fig. S7) (*46*), respectively; however, such features are not observed in spectra of Ryugu samples (fig. S8, A and B). A sharp absorption band at 5.83 μm (= 1715 cm^−1^ in wavenumber) is observed in the Ryugu individual grains A0026, A0055, A0063, A0064, A0067, C0023, C0025, C0040, and C0103 and coarse aggregate samples TD1 coarse, TD2 coarse, and glossy coarse (fig. S8, A and B). The 5.8-μm absorption band tends to be more common in Ryugu grains with a glossy surface (fig. S2). The 5.8-μm absorption band can be attributed to C=O stretching vibration in aldehydes (*50*, *51*). Some transmittance spectra of Ryugu fine particles and insoluble organic matter extracted from them exhibit absorption features at ~5.87 μm (1705 cm^−1^), but not at 5.83 μm (*52*). This may imply that the host materials of the 5.8-μm absorption band may occur only at the surface of Ryugu coarse grains or instantly react with the atmosphere.

### MIR reflectance spectra (8 to 18 μm)

**Fig. 4.**
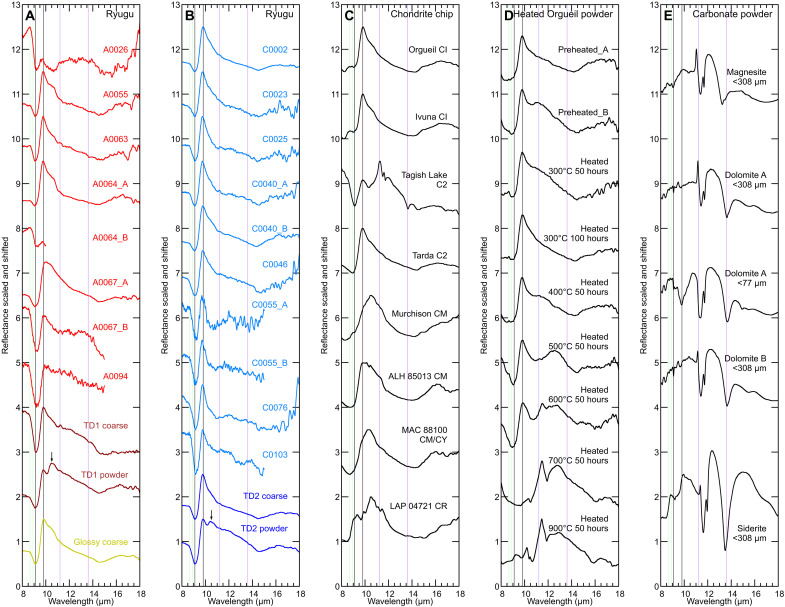
MIR (8 to 18 μm) reflectance spectra. (**A** and **B**) Ryugu samples, (**C**) hydrated carbonaceous chondrite chip samples, (**D**) experimentally heated Orgueil CI powder samples after exposure to air, and (**E**) carbonate powder samples. All the spectra are scaled to have the same difference between reflectance minimum and maximum and shifted arbitrarily. The vertical gray lines indicate 9.1 and 9.8 μm for Christiansen feature (CF) and Reststrahlen band (RB) peak of phyllosilicates, respectively. The vertical green lines indicate the peaks of sulfates in CI chondrites at 8.65 and 8.9 μm, which are not observed in Ryugu spectra. The vertical purple lines indicate the peaks of dolomite at 11.2 and 13.6 μm. Black arrows indicate 10.5 μm feature observed in Ryugu powder samples probably due to multiple reflection from a sapphire dish (*13*). MIR Ryugu spectra are almost homogenous and similar to that of Orgueil CI, Ivuna CI, and Tarda C2 chondrites, suggesting that Ryugu samples have saponite-rich phyllosilicate composition.

MIR reflectance spectra of Ryugu grains exhibit a Reststrahlen band (RB) with a maximum peak at 9.8 μm (Fig. 4, A and B) similar to spectra of CI and Tarda C2–ungrouped carbonaceous chondrites (Fig. 4C) dominated by phyllosilicates, particularly saponite-like ones [e.g., (*28*)]. This feature is notably different from what is observed for Murchison CM and LAP 04721 CR2 chondrites. Spectra of some Ryugu samples (e.g., A0055 and A0064) have a shoulder at ~10.5 μm in addition to the main RB feature, consistent with variability in phyllosilicate compositions, and serpentine in particular (*28*, *53*). The RB peak position of A0067 is shifted to a longer wavelength compared with other grains, and evidence of space weathering has been reported for this grain (*13*, *15*). Although not definitive, this suggests that space weathering may influence the position of the RB band for Ryugu materials, and a similar wavelength shift was reproduced by helium ion irradiation using Alais CI carbonaceous chondrites (see the “Spectral variations of Ryugu samples” section) (*54*).

Spectra of most Ryugu grains exhibit a 16-μm feature due to Mg-OH that is also commonly observed in hydrated chondrites (Fig. 4, A and B) (*25*), i.e., carbonaceous chondrites grouped as heating stage I (*23*). This indicates that Ryugu grains have avoided intense heating after aqueous alteration that would have led to the decomposition of phyllosilicates.

Speciation of carbonates is expected to reflect primary phases and aqueous alteration environments (*55*). Ryugu samples show a small peak at 11.20 μm due to out-of-plane bending (ν2) of carbonates, most consistent with dolomite (Fig. 4E) (*47*), suggesting parent body history similar to CI chondrites (*55*). In contrast, a large peak at ~11.25 μm in Tagish Lake spectrum indicates the presence of siderite, ankerite, or calcite (*47*).

The Christiansen feature (CF) of Ryugu samples is located at 9.1 μm, which is also similar to those of Tarda and Tagish Lake (Fig. 4, A to C). This is in contrast to CFs of Orgueil and Ivuna that are located at shorter wavelengths (Fig. 4, A to C). However, the two CI chondrites contain Ca and Mg sulfates that formed after falling on the Earth (*56*, *57*), and such phases can exhibit high v-band reflectance and peaks at ~8 to 9 μm (fig. S10A) (*49*, *58*). A whitish surface of Orgueil chip exhibits a higher v-band reflectance and more conspicuous peaks at ~8 to 9 μm than those of a sulfate-removed surface of Orgueil (fig. S10, B and C). Thus, the true CF of unmodified CI chondrites is likely masked by the presence of sulfates (Fig. 4C). Because of terrestrial alteration, an accurate spectral comparison of Ryugu grains to unheated CIs is not practical. However, heating of the CIs to minimize effects of hydrous sulfates reveals several spectral changes. First, the RB peak position of preheated Orgueil and Orgueil heated at 300°C for 50 and 100 hours and at 400°C for 50 hours indicates the phyllosilicate-rich composition for those samples, which is in good agreement with those of Ryugu samples (Fig. 4D). In addition, the CFs of preheated Orgueil are located at 8.7 to 8.8 μm, whereas those of Orgueil heated at 300° to 500°C are located at ~9 μm. This is consistent with sulfate dehydration by heating, and the latter are in better agreement with the Ryugu CF position (Fig. 4, A, B, and D). In the spectra of 500°C-heated samples, a broad peak appears near ~12.5 μm, which evolves to olivine-like spectral features seen in the sample heated to 600°C (Fig. 4D). The RB due to saponite-like phyllosilicates survives in 600°C-heated samples but disappears in the 700°C-heated samples, which is consistent with the mineralogy from XRD patterns. The MIR spectra of 700°C-heated and 900°C-heated Orgueil samples are dominated by the spectral features of olivine, the latter with a small contribution from pyroxene (*59*). MIR reflectance spectra indicate that Ryugu experienced CI-like aqueous alteration, and the mineral composition of Ryugu samples is reproduced by heating Orgueil CI samples gently to dehydrate terrestrial sulfates.

### UV spectra of Ryugu coarse grains and experimentally heated CI powder samples

UV reflectance spectra (0.2 to 0.4 μm) of Ryugu grains and the unheated and heated Orgueil powder samples were measured under ambient conditions by a UV spectrometer at incidence angle of 30°, emergence angle of 0°, and phase angle of 30° in the principal plane as described in the Supplementary Text. Ryugu samples A0055, C0040, and C0046 exhibit UV reflectance values of ~3% at 0.2 μm, whereas A0064 exhibits a higher reflectance (fig. S11, A and B). Fourier transform IR (FTIR) spectra of some Ryugu samples exhibit a concave-up feature toward UV wavelengths that is obviously different from the shapes observed in the visible region (Figs. 1 and 2), but these concave-up features could be an artifact or due to an anomalous reflection component. UV spectra of experimentally heated Orgueil powder samples show changes in reflectance values at 0.2 μm in the order of 900°C-heated >> 700°C-heated >> unheated > 400°C-heated > 500°C-heated > 600°C-heated (fig. S11C). This trend is consistent with that at visible wavelengths, where the samples heated at 300° to 600°C have darker spectra than the samples preheated or heated at 700° and 900°C (Fig. 2C). The samples exhibit little variation in spectral slope (fig. S11, C and F), and normalized UV spectra of preheated and heated Orgueil samples are similar to those of Ryugu samples regarding spectral redness (fig. S11, D to F). UV spectra of heated Orgueil samples show no marked changes other than in UV reflectance value, which is severely affected by the sample form and/or orientation; thus, we do not attempt to interpret degree of heating on Ryugu based on UV wavelengths.

### Spectral comparison of coarse and fine Ryugu and 300°C-heated Orgueil

Vis-NIR reflectance spectral properties are known to vary with sample grain size (fig. S12; see also the Supplementary Text) (*14*, *60*); thus, the Orgueil samples heated at 300°C for 50 hours were measured in two different forms, powder (<155 μm in size) and aggregates of millimeter-sized grains. This allows for a better comparison with Ryugu powder and coarse aggregate samples to account for effects related to grain size. We also investigated a powder sample heated at 300°C for 100 hours and a coarse aggregate sample heated at 300°C for 500 hours.

**Fig. 5.**
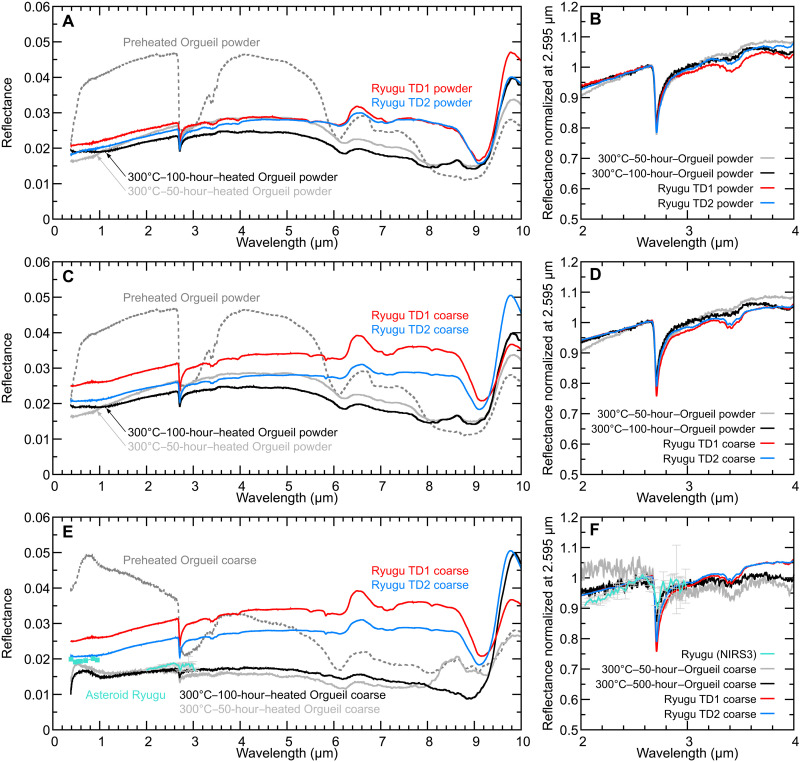
Spectral comparison between Ryugu and Orgueil. (**A** and **B**) Powder samples of Ryugu (*13*) and 300°C-heated Orgueil, (**C** and **D**) coarse aggregates of Ryugu (*13*) and 300°C-heated Orgueil powder, and (**E** and **F**) coarse aggregates of Ryugu (*13*) and 300°C-heated Orgueil. Ryugu TD1 and TD2 coarse and powder samples exhibit reflectance spectra similar to those of 300°C-heated Orgueil powder samples. Coarse aggregates of 300°C-heated Orgueil exhibit lower reflectance than Ryugu coarse aggregates, which is consistent with asteroid Ryugu spectra obtained by ONC-T/NIRS3 (*7*, *8*).

As described above, the major spectral features of TD1 and TD2 powder samples are low visible reflectance values of ~2%, slightly red-sloped Vis-NIR spectra, a metal-OH absorption strength of ~20% at 2.71 μm, and Si-O band at 9.8 μm. All these spectral features are reproduced by the Orgueil powder heated at 300°C (Fig. 5, A and B). Aside from the Vis-NIR spectral slope, there is little difference in Orgueil between 50 and 100 hours of heating at 300°C (Fig. 5A). The spectral similarities between Ryugu samples and Orgueil heated at 300°C suggest that they may have similar compositions.

When compared with the powder Orgueil sample, the aggregate samples of millimeter-sized Orgueil grains heated at 300°C exhibit a darker and flatter Vis-NIR spectrum and a shallower 2.7-μm band depth (Fig. 5E), which is consistent with previous measurements of carbonaceous chondrites of various grain sizes (*14*). In contrast, Ryugu sample spectra have only slight differences in v-band reflectance, Vis-NIR spectral slope, and the 2.7-μm band depth, depending on sample form (coarse aggregates and powder) (*13*). For instance, the 2.7-μm band depths of Ryugu powder and coarse samples are ~20% (Fig. 3A). Possible reasons why the band depth of Ryugu coarse and fine grains show little variation can be explained by the following: (i) grain size of Ryugu TD1 and TD2 powder samples (~1 mm of maximum grain size) is not fine enough to behave optically as fine powder [see figure S2 of (*13*)] compared to finer particle forms of other meteorite spectra; (ii) Ryugu coarse grains are coated by fine powder that makes reflectance spectra of millimeter-sized grains similar to those of fine powder (*14*); and /or (iii) Ryugu grains have low density [~1.8 g/cm^3^; (*13*)], and the associated highly porous surfaces exhibit spectral behavior similar to fine-grained powder samples. These explanations are in line with the fact that visible spectra of Tagish Lake powder, which is known to be fragile and with low density [1.66 ± 0.08 g/cm^3^; (*61*)], also do not exhibit large variations as a function of grain size (fig. S13).

The spectral features of TD1 and TD2 coarse samples are also well reproduced by Orgueil powder samples heated at 300°C (Fig. 5, C and D) rather than those of the coarse grains (Fig. 5, E and F). Again, this may be explained by the high porosity or the presence of fine particles coating coarse Ryugu grains. The TD1 and TD2 coarse samples were prepared without shaking off fine particles after sample distribution from JAXA. Fragmentation and friction of Ryugu samples during the sample collection and travel to the Earth may have contributed to crushing of Ryugu particles. In addition, the coarse aggregates of the heated Orgueil have Vis-NIR spectra and the 2.7-μm band depth (~10%) similar to those of Ryugu ONC-T/NIRS3 spectra (Fig. 5, E and F) (*7*, *8*). This would suggest that the undisturbed materials at the optical surface of asteroid Ryugu might have fewer fine particles and/or higher bulk porosity than the Ryugu samples that we analyzed. Needless to say, space weathering effects should be considered to discuss spectral differences between ONC-T/NIRS spectra and those of the returned samples (*14*).

### Atmospheric alteration effects on reflectance spectra of Ryugu powder sample

**Fig. 6.**
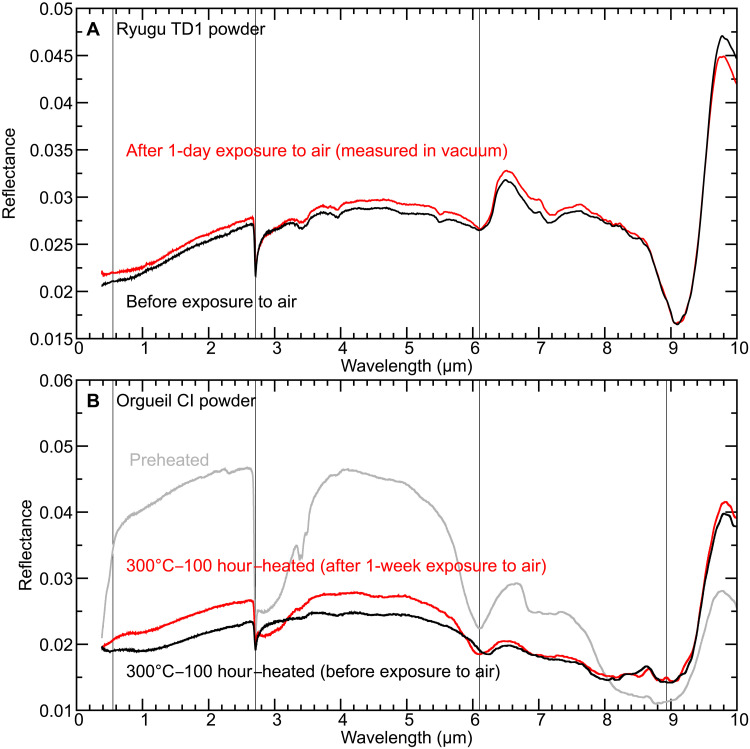
Spectral changes by atmospheric exposure. (**A**) Reflectance spectra of TD1 powder before (black) and after (red) exposure to air (*13*). The vertical gray lines indicate 0.55, 2.71, and 6.10 μm. The spectrum measured after exposure to air is brighter than the spectrum of the unexposed sample. The absorption features at ~3 and ~6 μm due to molecular water are more obvious in the spectrum of the exposed sample. (**B**) Reflectance spectra of Orgueil powder sample heated at 300°C for 100 hours before exposure to air (black), after exposure to air for a week (red), and the preheated Orgueil powder (gray; chip B). The vertical gray lines indicate 0.55 (v-band), 2.71, 6.10, and 8.93 μm.

The spectral similarity between Orgueil heated under reducing conditions and pristine Ryugu samples suggests that spectra of unmodified samples of the former are dominated by alteration due to exposure in the terrestrial environment, and it also suggests that spectra and composition of Ryugu samples may be susceptible to similar issues if exposed to ambient conditions. To explore these issues further, reflectance spectra of Ryugu TD1 powder sample (sample ID: A0106) were remeasured in vacuum (~3 hPa) after being left in air for 24 hours (*13*). Any observed spectral changes can be interpreted as the result of atmospheric alteration of the samples because the sample surface was not modified between the measurements, and the same area on the sample was analyzed. Even atmospheric exposure for a few tens of hours is expected to oxidize Fe^2+^ in saponite to Fe^3+^, although there should be no substantial rearrangement of the crystal structure (*62*). Exposure to ambient terrestrial conditions should also result in adsorption of H_2_O to grain surface, in pores, and possibly interlayer regions of phyllosilicates (i.e., rehydration) (*63*). The reflectance spectrum of the exposed Ryugu powder sample is brighter than that of the unexposed sample by 0.1% at v-band (Fig. 6A), possibly due to oxidation, and H_2_O absorption bands at 3 μm are slightly stronger, even when measured in vacuum. This is consistent with adsorption of atmospheric H_2_O and indicates even short-term exposure to terrestrial atmosphere likely results in irreversible water gain for Ryugu samples and, by extension, for similar carbonaceous chondrites.

### Atmospheric alteration effects on reflectance spectra of experimentally heated Orgueil

Potential spectral changes by exposure to terrestrial air were also evaluated by examining spectra of the heated (300°C) Orgueil powder sample in vacuum after it had been exposed to ambient conditions for 1 week (Fig. 6B). The sample surface was not modified between the measurements. The 3-μm absorption band is obviously stronger and broader after exposure, indicating rehydration by atmospheric water. The band center of the 6-μm band, which is affected by molecular water, carbonates, and organics (carbonyls), shifted to 6.1 μm, suggesting that H_2_O is dominating this spectral feature after exposure. Visible reflectance and NIR spectral slope are brighter and bluer, respectively, after exposure (Fig. 6B). The exposed sample exhibits a small peak at 8.9 μm probably due to S-O bending in sulfates (*49*), likely hydrated ones (fig. S10C). The spectrum of 300°C-heated Orgueil samples measured after air exposure has “intermediate” spectral features between those of the preheated and heated samples without air-exposure in terms of reflectance and hydration features (~3 and ~6 μm in wavelength), and, in general, the overall spectral shape of the reexposed sample appears to be reverting back to that of the preheated sample.

We exposed the Ryugu sample to the atmosphere for a shorter time than the heated CI sample because of the tight schedule of Ryugu sample analysis. Long-term atmospheric exposure of Ryugu samples should result in spectral changes in a similar manner to the spectral changes in the 300°C-heated Orgueil sample, but at a slower rate, considering the sample mineralogy before exposure. For instance, Ryugu samples do not initially contain sulfates (*13*), whereas the heated CI sample includes dehydrated sulfates that will rapidly become rehydrated by exposure to air.

### Vis-NIR spectral diversity among Ryugu coarse grains due to sample orientation

Most of Ryugu individual grains have rugged surfaces, but several grains have a few flat surfaces (*13*) that are expected to produce specular reflection under certain viewing geometry. The visible reflectance spectra of Ryugu grains show that a sample with high reflectance tends to have a redder-sloped spectrum (Fig. 1A). The samples with a flat surface such as grains of A0064 and A0067 show different v-band reflectance values, spectral slope, and the 2.7-μm band depth depending on the orientation of flat surfaces (Figs. 1A and 3A and fig. S1A). By comparing spectral properties with orientation of sample surfaces as simulated by x-ray computed tomography (XCT) data (fig. S14; see also the Supplementary Text), we find that the v-band reflectance and the b-x spectral slope increase and that the 2.7-μm band depth decreases when the specular reflection of a flat surface points close to the detector (i.e., local emergence angle of 0°) (fig. S15, A to C). Similar relationships between specular reflection direction, NIR reflectance, and NIR spectral slope were reported in a study of a flat surface of pressed meteorite powder samples with varying observation geometry (*64*). In addition, the concave-up feature from visible to UV wavelengths is also more apparent when the specular reflection direction is close to 0° (fig. S15D). For Ryugu grains, reflectance spectra that are substantially affected by specular reflection components are not common, but such spectra should be avoided when comparing Vis-NIR spectral properties of Ryugu samples to those of meteorites and asteroids, where diffuse spectral components dominate. In this context, the spectral data whose specular reflection direction range from 0° to 20° (A0064_settingA, A0067_settingA, and A0094) are excluded in the discussion hereafter. The average v-band reflectance, b-x slope, 2.7-μm band depth of the various remaining Ryugu sample groups in relation to sample forms are summarized in table S1.

### Heating experiments of Ryugu small grains

Heating experiments of Ryugu small grains were performed to constrain the maximum heating temperature that Ryugu samples have experienced. Ryugu small grains were heated stepwise from 100° to 500°C by 100°C for duration of 1 hour before being analyzed by synchrotron XRD (S-XRD) after each heating step. Unheated Ryugu grains consist of serpentine, saponite, pyrrhotite, magnetite, pentlandite, and dolomite (fig. S16). Diffraction patterns of Ryugu grains heated to 400°C were not considerably modified, but grains heated to 500°C exhibit weaker diffraction peaks associated with partial decomposition of phyllosilicates. This suggests that Ryugu samples did not experience heating at 500°C or above for 1 hour or longer.

### Mineralogical changes of Orgueil upon experimental heating

**Fig. 7.**
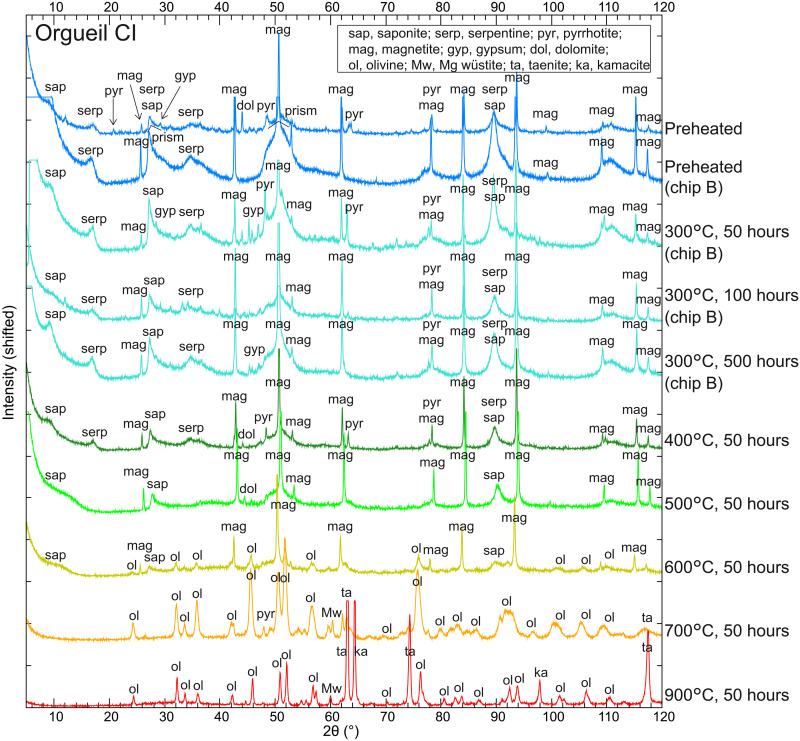
S-XRD patterns of matrices in experimentally heated Orgueil. Preheated Orgueil contains phyllosilicates, magnetite, carbonates, and gypsum. Orgueil samples heated at 300°C for 50, 100, and 500 hours consist of serpentine, saponite, magnetite, and carbonates. Orgueil heated at 400°C for 50 hours contains serpentine, saponite, magnetite, and carbonates. Orgueil heated at 500°C for 50 hours contains saponite, magnetite, and carbonates. Orgueil heated at 600°C for 50 hours consists of saponite magnetite, and secondary formed anhydrous minerals such as olivine. Orgueil heated at 700°C for 50 hours consists of olivine and Mg wüstite. Orgueil heated at 900°C for 50 hours consists of olivine, taenite, and kamacite. Orgueil powder samples heated at 300°C are originated from Orgueil chip B, whereas the other samples are originated from Orgueil chip A.

To characterize the mineralogy and associated compositional changes of the heated Orgueil samples, we performed S-XRD analysis of fine powder picked out of the matrices of the heated and air-exposed samples (Fig. 7 and table S2). Unheated Orgueil contains serpentine, saponite, magnetite, carbonates, pyrrhotite, and gypsum (hydrated Ca sulfate), and patterns exhibit prism reflections indicative of phyllosilicates. This is consistent with previous studies (*65*) and indicates that Orgueil samples used for the heating experiments have a typical composition of CI chondrites. Orgueil samples heated at 300°C for 50, 100, and 500 hours show diffraction peaks due to phyllosilicates, magnetite, carbonates, pyrrhotite, and gypsum. No obvious mineralogical changes were observed in the samples heated at 300°C for 50, 100, and 500 hours. Note that IR spectral peaks due to hydrated sulfates in the heated and unexposed samples nearly disappear by heating at 300°C (Fig. 4D); however, the fine particles analyzed by XRD were exposed to air after heating to have hydrated sulfates again. Orgueil heated at 400°C for 50 hours contains phyllosilicates, magnetite, carbonates, and pyrrhotite. However, the serpentine peaks are weaker than the preheated sample, and gypsum disappears, suggesting structural modification of serpentine and decomposition of gypsum. Heating at 500°C for 50 hours results in decomposition of serpentine, inferred from the disappearance of the (001) peak, but peaks due to saponite, magnetite, carbonates, and pyrrhotite remain. A basal spacing of saponite became slightly narrower along with heating however the observed change was not as large as expected for loss of interlayer water due to heating, being consistent with rehydration. Orgueil heated at 600°C for 50 hours consists of saponite, magnetite, and olivine, which suggests the coexistence of remnant phyllosilicates with secondary-formed anhydrous minerals. Orgueil heated at 700°C for 50 hours does not show any peaks attributable to hydrated minerals and consists of olivine and Mg wüstite. Orgueil heated at 900°C for 50 hours contains olivine, taenite, and kamacite. The peaks due to olivine and taenite in the 900°C-heated sample became more intense and sharper than those in the 700°C-heated sample, suggesting advanced crystallization. In summary, heating at 400°C for 50 hours caused partial decomposition of hydrated minerals, and heating to 600°C for 50 hours resulted in formation of secondary anhydrous silicates, whereas major mineral composition did not change by heating at 300°C for 50, 100, or 500 hours.

We examined a single CI chondrite; however, the observed mineralogical changes due to heating should be applicable to CI chondrites because their mineralogical heterogeneity is relatively small as a result of extensive aqueous alteration (*66*). Ungrouped C1 or CM1 samples may result in different behaviors according to their initial mineralogy. In comparison with Murchison CM2 samples, Orgueil CI samples seem more resistant to heating at lower temperatures (i.e., 300° to 600°C). Major hydrated phases in Murchison have lower decomposition temperatures than those in Orgueil; tochilinite and Fe-rich serpentine in Murchison decompose at 300° and 500°C (*63*), respectively, whereas saponite in Orgueil decomposes at 700°C (Fig. 7).

### Iron valence in phyllosilicates of unheated and heated Orgueil

**Fig. 8.**
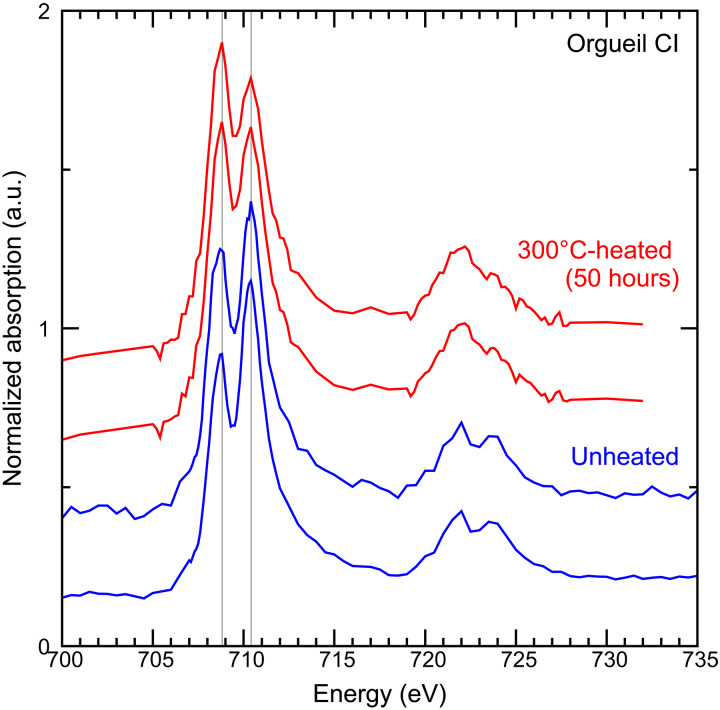
Normalized XANES spectra around Fe L-edge collected for phyllosilicates in unheated Orgueil (blue) and Orgueil heated at 300°C for 50 hours under a reducing condition (red). The XANES spectra of heated Orgueil were measured without exposing the sample to air. Unheated Orgueil shows a dominant absorption feature at 710.4 eV and an absorption feature at 708.8 eV, indicating that ferric iron is dominant in unheated Orgueil phyllosilicates (*13*, *62*). In the spectra of 300°C-heated Orgueil, an absorption feature at 708.8 eV is stronger than that at 710.4 eV, suggesting that the reduction of iron in phyllosilicates occurred by heating at low oxygen fugacity. a.u., arbitrary units.

To investigate typical valence state of iron in phyllosilicates of unheated Orgueil and Orgueil heated at 300°C for 50 hours under a reducing condition, x-ray absorption near edge structure (XANES) spectra around the Fe L-edge of the heated sample were collected without exposing the sample to air. Unheated Orgueil shows a dominant absorption feature at 710.4 eV and an absorption feature at 708.8 eV, whereas the latter is stronger than the former in the heated Orgueil (Fig. 8). This indicates that ferric iron is dominant in unheated Orgueil phyllosilicates (*62*), some of this iron is reduced by the heating under a reducing condition, and then ferrous iron becomes the dominant valence state for phyllosilicates in the heated sample. The ratio Fe^2+^/Fe_total_ in phyllosilicates of a Ryugu sample (ID: C0025) was examined before and after air exposure by micro–x-ray fluorescence spectroscopy (μ-XRF) with μ-XRF–XANES. This analysis revealed that Fe^2+^/Fe_total_ values were mostly >0.6 for Ryugu samples not exposed to air and values decreased to ~0.4 to 0.6 by exposure to air for 14 hours [figure S27 of (*13*)]. This demonstrates that atmospheric oxidation of ferrous iron in Ryugu phyllosilicates occurs quickly, and the Orgueil meteorite, which has been altered in terrestrial environments since it fell to the Earth, has been subject to similar processes. The experimental heating at 300°C for 50 hours under a reducing condition replicated the high Fe^2+^/Fe_total_ ratios in phyllosilicates of Orgueil without decomposing phyllosilicates, which replicates the composition of smectite in Ryugu samples.

### Carbon contents of Ryugu, unheated Orgueil, and heated Orgueil

There is a trend that chondrite samples with lower carbon contents exhibit higher Vis-NIR reflectance values among CI, CM, CR, CV, CY, and Tagish Lake samples (*29*). Thus, carbon contents are expected to affect Vis-NIR reflectance of carbonaceous chondrites. Within CM and CY chondrites, only a weak correlation between carbon content and v-band reflectance is reported, which can be interpreted as that the reflectance variability is also affected by other factors such as textural change due to intense heating of CY chondrites (*67*). Organic elemental analysis using FLASH 2000 (Thermo Fisher Scientific) revealed that carbon contents of unheated Orgueil and Orgueil heated at 500°C under vacuum conditions for 50 hours were 5.5 ± 0.1 wt % and 5.5 ± 0.0 wt % (±1σ; *n* = 3), respectively, which means that all or nearly all carbon remained even after heating at 500°C for 50 hours. The 3.4-μm absorption band due to aliphatic compounds (and carbonates) became weaker by heating to 500°C (Fig. 3D), suggesting structural changes in organics without substantial carbon loss. The diffraction peaks due to carbonates were still observed in the 500°C-heated Orgueil sample (Fig. 7). Despite the similarity in carbon content between the unheated and heated samples, spectra of the latter have a much lower v-band reflectance, indicating that factors other than carbon content influences this spectral property.

## DISCUSSION

### Spectral variations of Ryugu samples

Ryugu’s IR spectral properties are dominated by Mg-rich phyllosilicates (serpentine and saponite) and carbonates (Fig. 3). This suggests that the parent body of rubble pile Ryugu formed far from the Sun, incorporated ices of water and carbon dioxide, and that subsequent increases in temperature caused intensive aqueous alteration to convert primary silicate phases to Mg-rich phyllosilicates and carbonates. Other phases formed by the alteration include pyrrhotite and magnetite, but they were not detected in the spectra due to the lack of diagnostic absorption bands in the wavelength range probed (*68*, *69*). Small Vis-NIR spectral variations among Ryugu samples can be explained by variations in sample surface texture and orientation (fig. S15).

**Fig. 9.**
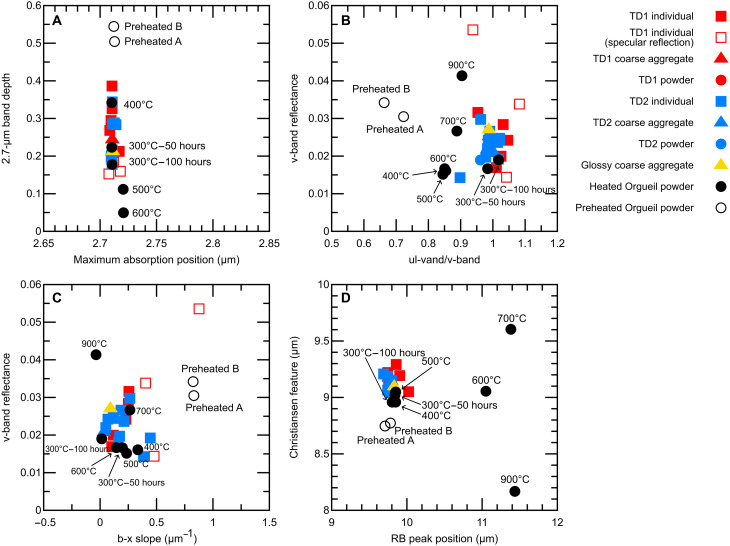
Spectral parameters of Ryugu samples compared to experimentally heated Orgueil powder samples. (**A**) The band depth and the maximum absorption position of the 2.7-μm absorption band. (**B**) The v-band reflectance and the reflectance ratio of ul-band and v-band. (**C**) The v-band reflectance and the spectral slope from b-band to x-band. (**D**) The peak position of CF and RB of Si-O stretching.

TD1 samples and TD2 samples were collected from different areas on asteroid Ryugu, the latter possibly containing subsurface material excavated by SCI operation (*36*). An onboard camera revealed that the general appearance of the first and the second touchdown sites was similar (*11*), and the spectral properties of TD1 and TD2 samples are not notably different (Fig. 9). This indicates a lack of heterogeneity at millimeter scale. Less aqueously altered fragments (several hundreds of micrometers in size) with olivine and pyroxene have been reported in Ryugu samples (*13*, *70*). However, spectral features associated with anhydrous silicates were not observed at ~1 μm or MIR wavelengths, consistent with their overall low abundance (Figs. 1 and 4). This is consistent with most Ryugu samples being derived from fragments that experienced severe aqueous alteration.

A few Ryugu grains display features indicative of space weathering, including amorphous layers due to solar wind irradiation and melt layers due to micrometeoroid bombardment (*15*). The NIR spectral survey on Ryugu millimeter-sized grains at JAXA suggests that a small but substantial fraction of TD1 samples show signs of space weathering (*71*), but space-weathered grains were not a major component of the Ryugu samples probed in this study. Exceptions include the grains A0067 and A0094, which have a thin amorphous layer covering the uppermost part of phyllosilicates, an expected product of the earliest stage of surface modification due to space weathering (*15*). Such weathering processes probably result in decreases in the 2.7-μm band depth (*15*). EMG fitting analysis showed that the maximum absorption position of the 2.7-μm band of A0067 is shifted toward a longer wavelength by ~6 to 8 nm compared to typical Ryugu grains (fig. S17), with a wavelength resolution of ~3 nm at 2.71 μm (table S3). The RB peak positions (~10 μm) of A0067 and A0094 are shifted toward longer wavelengths than those of typical Ryugu samples (fig. S18). Similar wavelength shifts of the 2.7-μm band maximum absorption and RB peak positions were produced by helium ion irradiation experiments on Alais CI chondrite that simulated solar wind irradiation, probably due to selective sputtering of Mg in phyllosilicates and amorphization (*54*). Vis-NIR reflectance spectral changes due to heavier surface modification of simulated meteorites have been reported (*54*, *72*); however, we did not identify notable differences in Vis-NIR spectra of space-weathered grains (A0067 and A0094) likely because the sample orientation and the surface texture are more affective on Vis-NIR spectral measurements of Ryugu samples (fig. S15). Heavily space-weathered Ryugu grains, for instance, grains with melt layers or melt splashes (*15*), may have distinct Vis-NIR reflectance spectra.

Collectively, Ryugu sample spectra are mostly homogenous at millimeter scale, which indicates extensive aqueous alteration in the parent body. Ryugu grains that experienced space weathering or less intense aqueous alteration are minor in our sample suite but can be detected by the IR reflectance properties.

### Comparison of Ryugu samples and unheated CI chondrites

Mineralogical and chemical analyses revealed that Ryugu samples are similar to CI chondrites and did not experience heating that was intense enough and sufficient duration to decompose phyllosilicates (*13*, *16*). Nevertheless, the reflectance spectra of Ryugu samples do not clearly match those of natural CI chondrites (Fig. 9) (*4*). As demonstrated above, this is primarily because the reflectance spectra of natural, unheated CI chondrites are severely influenced by the presence of phases formed during prolonged terrestrial weathering. In addition to adsorption of atmospheric H_2_O, such weathering has led in particular to the formation of secondary sulfates (*35*, *56*, *57*) and ferrihydrites (*66*, *73*), rehydration (*27*, *74*), and oxidation of Fe^2+^ in phyllosilicates (*13*). We now discuss each of these points and the corresponding spectral effects.

The matrices of Ryugu grains are rich in small pyrrhotite grains (*13*), which is distinct from those of Orgueil (*75*). Abundant opaque minerals may work as a darkening agent for Ryugu samples, considering that previous studies have shown that the addition of Fe sulfide (troilite) can make the reflectance spectrum of silicate (forsterite) darker and blue-sloped (*69*, *76*). Abundant tiny sulfides can be a precursor of sulfate veins found in CI chondrites (*13*, *56*, *57*), and Ca sulfates might have been derived from alteration of Ca carbonates and sulfides in Orgueil (*13*). The formation of Mg sulfate [epsomite (MgSO_4_ (H_2_O)_7_] was observed on a thin section of Ivuna CI chondrite within 6 years (*57*). Sulfates commonly exhibit high v-band reflectance values {e.g., 0.9 and 0.4 for powder samples (<45 μm in size) of gypsum [CaSO_4_ (H_2_O)_2_] and kieserite [MgSO_4_ (H_2_O)], respectively; fig. S10A} (*49*) and peaks at ~9 μm due to SO_4_ bending, resulting in the CF shift of CI chondrites toward a shorter wavelength (fig. S10C) (*49*, *58*). A more recent study demonstrated that even low abundances of highly hydrated sulfates can have substantial effects on spectra of CM chondrites, particularly for bulk spectral measurements (*35*).

Molecular water gives rise to strong absorption features at ~3 and ~6 μm (*32*–*34*). The lack of a strong and broad absorption feature at ~3 μm in Ryugu grains indicates little water, including interlayer H_2_O in smectite, is present in the samples (Fig. 3, A and B). The water released from Ryugu samples at a temperature lower than 300°C is much less than that from Ivuna CI chondrite (*16*). Water released from CI chondrites at <300°C is of terrestrial origin (*74*), which means that Orgueil CI chondrite has been interacting with atmospheric water since its fall to the Earth in 1864 to obtain abundant terrestrial water such as adsorbed water, interlayer water in phyllosilicates, and water/hydroxyl in ferrihydrite and sulfates [e.g., (*27*)]. After heating at 250°C under vacuum conditions, spectra of CR1 and CI1 chondrites show sharper 2.7-μm absorption features and lower visible reflectance than before heating [figure 1 of (*30*)]. Heating at 250°C is not enough to completely decompose many hydrous minerals (Fig. 7), but it is strong enough to remove loosely bound or adsorbed molecular water and to decompose ferrihydrite (*27*), which may cause a decrease in the visible reflectance of carbonaceous chondrites.

The Fe^2+^/Fe_total_ ratio in phyllosilicates of unheated Orgueil is determined as ~0.1 to 0.2, which is much lower than that of Ryugu (~0.5 to 0.7) (*13*) and the heated Orgueil samples (Fig. 8). By oxidation of Fe^2+^ [0.76 to 0.03 in Fe^2+^/Fe_total_, comparable to the oxidation states of Ryugu sample and unheated Orgueil, respectively; (*13*)] in Mg-rich smectite, visible reflectance and Vis-NIR spectral redness increased [figure 6D of (*77*)].

Those oxidation and hydration reactions that occurred to Orgueil can account for the majority of spectral differences between Ryugu samples and unheated Orgueil. The spectral changes from the 300°C-heated Orgueil sample before air exposure to that after air exposure clearly demonstrate some of the spectral influence of terrestrial weathering and exposure imparts on CI meteorites and demonstrate that some processes induced by heating under vacuum conditions are reversible (Fig. 6B).

### Spectral similarities between Ryugu samples and weakly heated CI chondrites

We conducted the heating experiments of Orgueil under vacuum and reducing conditions. The CI sample heated at 300°C for 100 hours shows the best spectral match with Ryugu samples, reflecting the mineralogical and chemical similarities between Ryugu samples and the Orgueil samples heated at 300°C, rather than unheated Orgueil sample. Despite strong spectral similarities between Ryugu samples and Orgueil samples heated at 300°C under vacuum and reducing conditions, this does not imply heating at 300°C of the asteroid Ryugu. Heating at 300°C caused reduction of iron in phyllosilicates and dehydration of sulfates in the Orgueil samples, whereas Fe^2+^-rich phyllosilicate compositions and no sulfates are expected to result from the aqueous alteration environments of Ryugu (*13*). Although abundant interlayer water would occur when saponite formed in the aqueous alteration of Ryugu’s parent body with relatively high water-rock ratio (*13*), interlayer water in phyllosilicates of the Ryugu surface materials [~tens of centimeters in depth; (*7*, *78*)] can be removed by repeated diurnal heating [~80°C at TD1 region, ~60°C at TD2 region; (*10*, *79*)] at the current orbit [~1.2 astronomical units from the Sun; (*5*)]. The mild heating at <100°C is not enough to decompose phyllosilicates in Ryugu (fig. S16, A and B) but can be effective to remove most interlayer water of phyllosilicates in CI chondrites (*32*).

To summarize, the experimental heating of CIs under reducing conditions caused removal of terrestrial water, reduction of iron in phyllosilicates, and dehydration of terrestrial weathering products that are now inherent to CIs, producing a sample whose spectrum is presumably more similar to fresh CI material and which is much more consistent with spectra of fresh Ryugu samples.

### Reassigning CI parent bodies considering terrestrial and space weathering

The Orgueil CI chondrite, Ryugu samples, and the surface materials of asteroid Ryugu have major mineral compositions that are consistent with the origin from similar aqueous alteration conditions (*13*). However, the reflectance spectra of these three materials are notably different (Fig. 5E) (*13*, *14*). Spectral differences between returned Ryugu samples and CI chondrites are much larger than those between spacecraft observations of asteroid Ryugu and lab spectra of Ryugu samples [Fig. 5; figure 2 of (*13*)]. The reflectance spectra of Ryugu samples have a deeper absorption feature at 2.7 μm than those of asteroid Ryugu, suggesting that the uppermost and very thin surface layer of asteroid Ryugu has been modified because of space weathering (*8*, *13*–*15*). The Ryugu samples analyzed in laboratories are interpreted to be fresher than the actual surface materials of the asteroid, possibly due to crushing and fragmentation during sample collection and the journey to the Earth (*13*–*15*). Whereas space weathering of asteroidal materials occurred to the very surface materials under reducing conditions (*15*), meteorites from aqueously altered parent bodies have experienced terrestrial alteration in oxidative environments (*3*, *56*, *57*). In particular, CI chondrites have fine-grained matrix and high porosity (*56*, *75*), which allows pervasive interaction of atmospheric oxygen and water with the meteorites. This implies terrestrial weathering of CI chondrites has more severely modified the reflectance spectra of the actual materials of CI parent bodies than space weathering.

C-complex asteroids, particularly those with strong drops in reflectance at UV wavelengths (UV drop-off features), have been considered as parent bodies of CI chondrites based on the spectral similarities (*80*), but reevaluation may be in order due to spectra of CI chondrites being severely influenced by terrestrial weathering products. This study implies that some of C-complex asteroids without UV drop-off features and X-complex asteroids are likely to be CI parent bodies. CI parent bodies in near-Earth orbit may have narrow 2.7-μm absorption features due to diurnal heating, which is nearly undetectable by ground-based observations due to telluric water. The presence of asteroids that experienced aqueous alteration followed by thermal alteration is suggested on the basis of the Vis-NIR spectral similarities between heated CM/CI chondrites and C-complex main-belt asteroids (*21*, *22*); however, those spectral similarities cannot be direct evidence of the thermal metamorphism of the asteroids as discussed above. Therefore, this study suggests that there may be more objects that are volatile-rich distributed in the main asteroid belt and near-Earth orbit than estimated previously. These results highlight how simplistic spectral comparisons between carbonaceous chondrites and asteroids may yield an incomplete and/or inaccurate estimation of the compositional distribution in the solar system.

## MATERIALS AND METHODS

### Reflectance spectroscopy of Ryugu samples

Reflectance spectra of six coarse grains (A0026, A0055, A0063, A0064, A0067, and A0094) collected from the first touchdown site (TD1) and seven coarse grains (C0002, C0023, C0025, C0040, C0046, C0076, and C0103) collected from the second touchdown site (TD2) were measured one by one as “individual grains.” Sample ID, size, and density are summarized in table S4. After the journey from the JAXA/ISAS curation facility to Tohoku University without exposure to air, these coarse grains were introduced into a pure N_2_-purged chamber in which the dew point was under −55°C and oxygen concentration was under 10 parts per million. Coarse grains were individually placed on a black sheet (Fineshut-Kiwami; KOYO Orient Japan Co. Ltd.), which has a 0.8% reflectivity at Vis-IR wavelength (fig. S19), set on a stainless-steel sample dish. Almost no reflection from a Kiwami sheet was expected. This dish is hereafter referred to as a dark dish. Reflectance spectra of four coarse Ryugu grains (A0064, A0067, C0040, and C0055) were measured twice in different placements to investigate variable composition on different surfaces and the effects of sample orientation on spectral properties. We report each of the spectra.

The aggregates of seven coarse grains from chamber A (A0026, A0055, A0058, A0063, A0064, A0067, and A0094) and seven coarse grains from chamber C (C0023, C0025, C0033, C0040, C0061, C0076, and C0103) were put onto a dark dish and analyzed as TD1 coarse and TD2 coarse, respectively. Powder samples (sample IDs: A0106 and C0107) were provided from the JAXA curation facility each in a sapphire dish (8 mm in size). A0106 (38.4 mg in weight) mainly consists of 1-mm-sized particles collected by TD1, and C0107 (38.8 mg in weight) consists of both several 1-mm-sized particles and fine particles collected by TD2. They were analyzed as TD1 powder and TD2 powder, respectively. Powder samples were left in the sapphire dish even during measurements. Reflectance spectra of TD1 coarse, TD2 coarse, TD1 powder, and TD2 powder are reported in (*13*). Observation of grain surfaces with a binocular revealed that eight coarse grains (A0064, A0067, A0094, C0025, C0033, C0061, C0076, and C0103) have a glossy and smooth surface (fig. S2). They were placed onto a dark dish and spectroscopically analyzed as glossy coarse. C0002, TD1 coarse, TD2 coarse, and glossy coarse samples were measured at three different azimuth conditions (0°, 120°, and 240° in rotation angles) to average specular reflection and other anomalous effects of surfaces. We report averaged spectrum.

A dark or sapphire dish was put into an air-tight cell with reflectance standards, Spectralon and Infragold (Labsphere). The cell was sealed either by a CaF_2_ window and a ZnSe window for the measurements from 0.38 to ~8 μm and from ~8 to ~18 μm, respectively, taken out from the N_2_-purged chamber, and then put into a Bruker FTIR VERTEX 70v. The sample had never been exposed to the air through curation at JAXA, transportation from JAXA, the sample preparation, and measurements to avoid effects of terrestrial alteration such as hydration and oxidation by the atmosphere.

Reflectance spectroscopy of Ryugu samples was performed using a Bruker VERTEX 70v FTIR spectrometer at Tohoku University in five wavelength ranges with the different settings, including spectral resolutions, as shown in table S3. The outside of the sealed cell was vacuumed until ~3 hPa, whereas the inside was filled with nitrogen (~1000 hPa) during the measurements. A beam size was modified from ~1 to 5 mm in diameter depending on samples to stick out from sample surfaces as little as possible. Reflectance spectra were measured at incidence, emergence, and phase angles of 30°, 0°, and 30° in the principal plane, respectively.

Obtained reflectance was corrected using reflectance values of Spectralon provided from Labsphere. Sample orientation and consequent specular reflection or other anomalous reflection from a surface affect near-UV spectral shape; therefore, near-UV spectra are not used for further analysis. The 2.7-μm band depth was calculated using the EMG profile (*42*) after normalization by a linear continuum between the two outermost ends of the entire 2.7-μm absorption feature. The details are described in the Supplementary Text.

Meteorite chip samples such as Orgueil CI, Ivuna CI, Tagish Lake C2–ungrouped, Tarda C2–ungrouped, LAP 04721 CR, Murchison CM, ALH 85013 CM, and MAC 88100 CM/CY were analyzed using the same FTIR system at Tohoku University. The details are summarized in table S5.

### Heating experiments of Orgueil CI chondrite

We performed heating experiments of Orgueil CI chondrite to investigate mineral and spectral changes with heating. Several chips of Orgueil (CI) chondrite (~5 mm in diameter) were used in this study. Before heating, small white particles of sulfates, such as epsomite (MgSO_4_·7H_2_O) and gypsum (CaSO_4_·2H_2_O) in the Orgueil sample, were removed by hand using an edged tool because it is thought to be a terrestrial weathering product (*56*, *57*). Then, some chips of Orgueil (~500 mg in total weight) were ground and sieved into <2000, <512, and < 155 μm in grain size by step for reflectance spectroscopy. We prepared Orgueil powder sample (<155 μm in size and ~60 mg in weight) and a few coarse grains (~1 mm in size) for each experimental heating temperature: 400°, 500°, 600°, 700°, and 900°C. Samples were experimentally heated for 50 hours under vacuum conditions (~10^–6^ torr) at Iron-Wüstite buffer using iron powder (~400 mg) beside the heated samples. “Preheated sample” was also prepared, and, before spectral and water analysis, it was preheated at 150°C for 3 hours to remove adsorbed water. The released water of Orgueil by heating at a temperature below 300°C should be of terrestrial origin, which displays Δ^17^O values close to that of the terrestrial fractionation line (*74*). Other specimens of Orgueil were used for the heating experiments at 300°C with various durations. We measured reflectance spectra of the powder samples heated at 300°C for 50 and 100 hours and those of the aggregates of coarse grains (~1 mm in size) heated at 300°C for 50 and 500 hours.

Heated and preheated Orgueil powder samples (~40 mg) were placed in a stainless-steel dish (8 mm in diameter) in an N_2_-purged glove box whose humidity was <1% and O_2_ fugacity was <0.1%. The aggregates of coarse grains were placed on a dark substrate in a similar way to Ryugu samples [this study and (*13*)]. A sample was packed into an air-tight cell with Spectralon and Infragold (Labsphere). The cell was sealed with a CaF_2_ window for FTIR measurements. Spectral analyses of heated and preheated Orgueil were performed in a similar way to Ryugu samples. The heated Orgueil samples were characterized by multiple ways and the details are described in table S6.

## Supplementary Materials

### This PDF file includes:

Supplementary Text Figs. S1 to S19 Tables S1 to S7 References
