# Supplementary material for: Reassigning CI chondrite parent bodies based on reflectance spectroscopy of samples from carbonaceous asteroid Ryugu and meteorites
Source: Sci Adv. 2023 Dec 6;9(49):eadi3789. doi: 10.1126/sciadv.adi3789 (PMC10699774; doi:10.1126/sciadv.adi3789)
Supplement: Supplementary file 1 — Supplementary Text Figs. S1 to S19 Tables S1 to S7 References [file sciadv.adi3789_sm.pdf]

Science Advances
AAAS

Supplementary Materials for

# Reassigning CI chondrite parent bodies based on reflectance spectroscopy of samples from carbonaceous asteroid Ryugu and meteorites

Kana Amano *et al.*

Corresponding author: Kana Amano, amakana@tohoku.ac.jp

*Sci. Adv.* **9**, eadi3789 (2023)
DOI: 10.1126/sciadv.adi3789

**This PDF file includes:**

Supplementary Text
Figs S1 to S19
Tables S1 to S7
References

**Supplementary Text**

The spectral parameters to characterize visible reflectance spectra of samples

To discuss visible reflectance spectral properties, reflectance values at several narrow wavelength bands were calculated referring to the seven color band-pass filters, or ul-, b-, v-, Na-, w-, x-, and p-band, included in ONC-T onboard the spacecraft Hayabusa2 (*6*). The characteristics of the wavelength bands are summarized in Table S7. Each band reflectance was calculated by averaging the reflectance values within the band. Spectral slope from b-band to x-band, referred to as "b-x (spectral) slope", was calculated as follows:

$$slope_{b-x}\ (\mu m^{-1}) = \frac{R(x) - R(b)}{R(v) \cdot (x - b)}$$

where $slope_{b-x}$ is the b-x slope, $R(x)$, $R(b)$, $R(v)$, are the x-, b-, and v-band reflectance, respectively, and $x$ and $b$ are the center wavelengths of the x- and b-band, respectively.

Spectral deconvolution using Exponentially Modified Gaussian (EMG) models

The spectral deconvolution of the 2.7-μm absorption band was performed using Exponentially Modified Gaussian (EMG) profiles with the HADeS (Hydration Absorption band Deconvolution Software) algorithm as described in (*42*). This algorithm first normalizes the data by a linear continuum between two wavelengths located at each side of the band on the outside of the absorption. The software performs a spectral fit of the normalized reflectance following the χ2 minimization of the Levembert-Marquardt algorithm. The band parameters are calculated on the complete band and each component, using the following statements. The position of the band or component is considered as the wavelength at which the minimum of reflectance within the band is found. The band depth, or amplitude, is calculated using the following equation:

$$Band\ Depth = 1 - \frac{R_{\lambda c}^{measurement}}{R_{\lambda c}^{continuum}}$$

With λc is the position of the band or component, and $R_{\lambda c}^{measurement}$ and $R_{\lambda c}^{continuum}$ are the reflectance of the continuum and the measured reflectance at the wavelength λc, respectively. Finally, the width of the band or component is considered as the Full Width at Half-Maximum (FWHM).

The number of components and the wavelengths at the points of contact with the continuum were adjusted for each spectrum. The quality of the fitting model was evaluated by the difference between the modeled and measured spectra in the wavelength range considered, i.e. the residue. The fitting model with the lowest residue was considered the best model. No constraints were applied to the position and amplitude of the components.

The errors on the fit model and the calculated band parameters are determined using the bootstrap statistical method. This necessitates the fit and calculation of the parameters a large number of times after addition of a small fluctuation on the data. Here 500 iterations of the bootstrap are performed. This leads to a list of 500 models, thus 500 series of band and component parameters. The distributions of these parameters allow the consideration of their center as the best fit, and their FWHM as the corresponding error. The fluctuations added to the data for the bootstrap method should correspond to the measurement error. In this case, we considered the measurement error as the standard deviation of the residue.

EMG fitting was performed without all information on the measurements and the samples in order to remove bias regarding possible links between spectra of the same sample or environmental conditions.

Ultraviolet reflectance spectroscopy

Ultraviolet (UV) reflectance spectra (0.20–0.40 µm in wavelength) of heated and unheated Orgueil powder samples were measured using a UV spectrometer equipped with a deuterium light source (H2D2 L11799, Hamamatsu Photonics K.K.) and a Maya2000 PRO spectrometer (Ocean Optics, Inc.) at Tohoku University. The measurements were performed in ambient conditions and with an incidence angle of 30° and an emission angle of 0°. Samples were placed in a stainless-steel dish and covered by a dark aperture made of black flock paper to avoid effects of stray light. A quartz powder was used as a standard material and the reflectance ratio of a sample to the quartz powder was calculated in the following way:

$$R'_{sample} = \frac{I_{Sample(30°,0°)}}{I_{Quartz(30°,0°)}} \times \frac{I_{Quartz(15°,15°)}}{I_{VUVmirror(15°,15°)}} \times coef_{400nm}$$

where $R'_{Sample}$ is the reflectance of a sample to the quartz powder, $I_{Sample(30°,0°)}$ and $I_{Quartz(30°,0°)}$ are the measured intensities of a sample and quartz powder at (i, e)=(30°, 0°), respectively, $I_{Quartz(15°,15°)}$ and $I_{VUVmirror(15°,15°)}$ are the measured intensities of the quartz powder and Vacuum UV (VUV) mirror (Edmund Optics) at (i, e)=(15°, 15°), respectively, and $coef_{400nm}$ is the reflectance ratio at 400 nm of the quartz powder measured by FTIR at (i, e)=(30°, 30°) to that measured by the UV spectrometer (i, e)=(15°, 15°). Note that VUV mirror exhibits >85% and >80% reflectance at >220 and 170–220 nm, respectively.

The reflectance values of a sample were calculated using a wavelength calibration standard (Labsphere, ID: CSTM-WCS-MC, a whitish disk made of the oxide of a rare earth element) and 99% Spectralon (Labsphere, ID: HL-TXH-025-A, RELAB) in the following way:

$$R_{sample} = R'_{sample} \times \frac{I_{Quartz(30°,0°)}}{I_{CSTM-WCS-MC(30°,0°)}} \times \frac{I_{CSTM-WCS-MC(30°,0°)}}{I_{99\%Spectralon(30°,0°)}}$$

where $R_{sample}$ is the reflectance of a sample and $I_{Quartz(30°,0°)}$, $I_{CSTM-WCS-MC(30°,0°)}$, and $I_{99\%Spectralon(30°,0°)}$ are the measured intensities of the quartz powder, the wavelength calibration standard, and the 99% Spectralon, respectively. The absolute reflectance of 99% Spectralon from 200 to 400 nm was assumed as 1 because the absolute values provided by Labsphere only covers longer wavelengths than 250 nm. A blackout curtain covered the system during measurements to avoid stray lights. UV spectra were normalized by the average reflectance at 390–400 nm.

Grain size effects on reflectance spectra of Orgueil CI chondrites

Reflectance spectra of Orgueil samples sieved to different size fractions were measured to investigate grain size effects on the spectral features. The Vis-NIR reflectance spectra of Orgueil with a maximum grain size of 2000, 512, and 155 µm and the chip sample are characterized by an intense UV drop-off feature, a small shoulder at ~0.5 µm attributed to Fe oxides/hydroxides, an absorption band at 1.95 µm due to hydrated minerals, including gypsum, and an absorption band at ~2.3 µm due to Mg-OH (*39*). The chip sample shows the brightest and bluest spectrum at visible wavelengths, whereas the powder samples with a maximum grain size of 2000 µm have the lowest reflectance and blue-sloped spectra (Fig. S12A and B). The samples with a grain size of <512 and <155 µm show redder and brighter spectra than those with a grain size of <2000 µm.

The metal-OH absorption band depth at 2.71 µm due to Mg-rich phyllosilicates, which is a distinct spectral feature of Orgueil, increases, relative to the adjacent $H_2O$ absorptions, with decreasing grain size (Fig. S12C). The 2.71 µm OH band remains distinct from the $H_2O$ bands at ~2.8–3.1 µm, allowing its strength to be accurately determined. The MIR region exhibits a

reflectance peak due to phyllosilicates (~9.8 μm) (*28*) and small peaks due to sulfates (8.65 and 8.90 μm) (*49*, *58*), none of which exhibit significant changes due to grain size when they are scaled (Fig. S12D).

Surface shape model based on X-ray computing tomography

Synchrotron X-ray computed tomography (SR-CT) data of grains was obtained at BL20XU Spring-8 after spectroscopic measurements at Tohoku University, and was used to investigate how a surface was oriented with respect to incident and emittance angles for different spectral measurements. The details of SR-CT analytical procedures are described in supplementary materials of (*13*). A microscopic shape model of the surfaces from which reflectance spectra were measured was produced using CT data based on a picture of each coarse grain taken before spectral measurement to clarify the sample orientation and the footprint of incident light on the sample surface. CT data using binning of 8 and 4 voxels of original CT data with 0.85 micron/voxel of spatial resolution for C0002 and the other coarse grains, respectively.

A normal vector at each sample surface gridded by micron-scale was calculated to evaluate the relationship (or correlation) between sample surface tilt or orientation and the phase angle used for reflectance spectral measurements. Figure S14 shows sample surfaces irradiated by incident light as a projection of a point in the direction perpendicular to the plane of the incident and emittance angle (y-z plane). Black and blue arrows indicate the direction of the incident angle (30°), the mean value of the normal vector to each small area (micrometer-scale) on the surface of the sample, respectively. Red arrows indicate the specular reflection directions calculated from the angles of incidence and the normal vector of the sample assuming that the sample has a specular surface. The red arrows of A0064_settingA (Fig. S14B) and A0067_settingA (Fig. S14D) point toward the detector, suggesting that anomalous effects by specular reflection are intense when the irradiated surfaces are flat enough to produce a mirror-like surface. For instance, the red arrow in A0067_settingA (Fig. S14D) points closer to the direction of the detector (0°) than that in A0067_setting B (Fig. S14E), which suggests that reflectance spectra measured from A0067_settingA (Fig. S14D) should be affected more strongly by specular reflection from the surface than that from A0067_setting B (Fig. S14E).

Synchrotron-based X-ray diffraction (S-XRD)

Fine particles (<155 μm in size) picked up from matrix grains of heated and unheated Orgueil samples were analyzed to identify their mineral phases by Synchrotron X-ray diffraction analysis using a Gandolfi camera at undulator beam line 3A in the Photon Factory, High Energy Accelerator Research Organization, Japan. For this experiment, each particle was glued using glycol phthalate to the top of carbon fiber that stands on a thin glass tube. X-rays at a wavelength of 2.17 Å were applied to the samples, and the exposure time was 13–20 minutes depending on the sample size.

Scanning transmission X-ray microscopy (STXM) and μ-XRF X-ray absorption near edge structure (XANES) analysis

To investigate average $Fe^{2+}/Fe_{total}$ in typical phyllosilicates in a sample, scanning transmission X-ray microscopy and μ-XRF X-ray absorption near edge structure (XANES) analysis at Fe K-edge using a 1.0 μm × 1.0 μm X-ray beam was performed in air-tight conditions for a particle of Orgueil (~150 μm in size) heated at 300 °C for 50 hours in a reduced condition. A particle of the

unheated Orgueil was also analyzed in the same way for comparison. Detailed analytical procedures are described in (*13*).

Elemental analysis for carbon contents

Carbon contents of unheated and 500 °C-heated Orgueil were measured using an Elemental Analyzer (EA), FLASH 2000 (Thermo Scientific) at Tohoku University. Powder samples (~2 mg in weight) were analyzed three times.

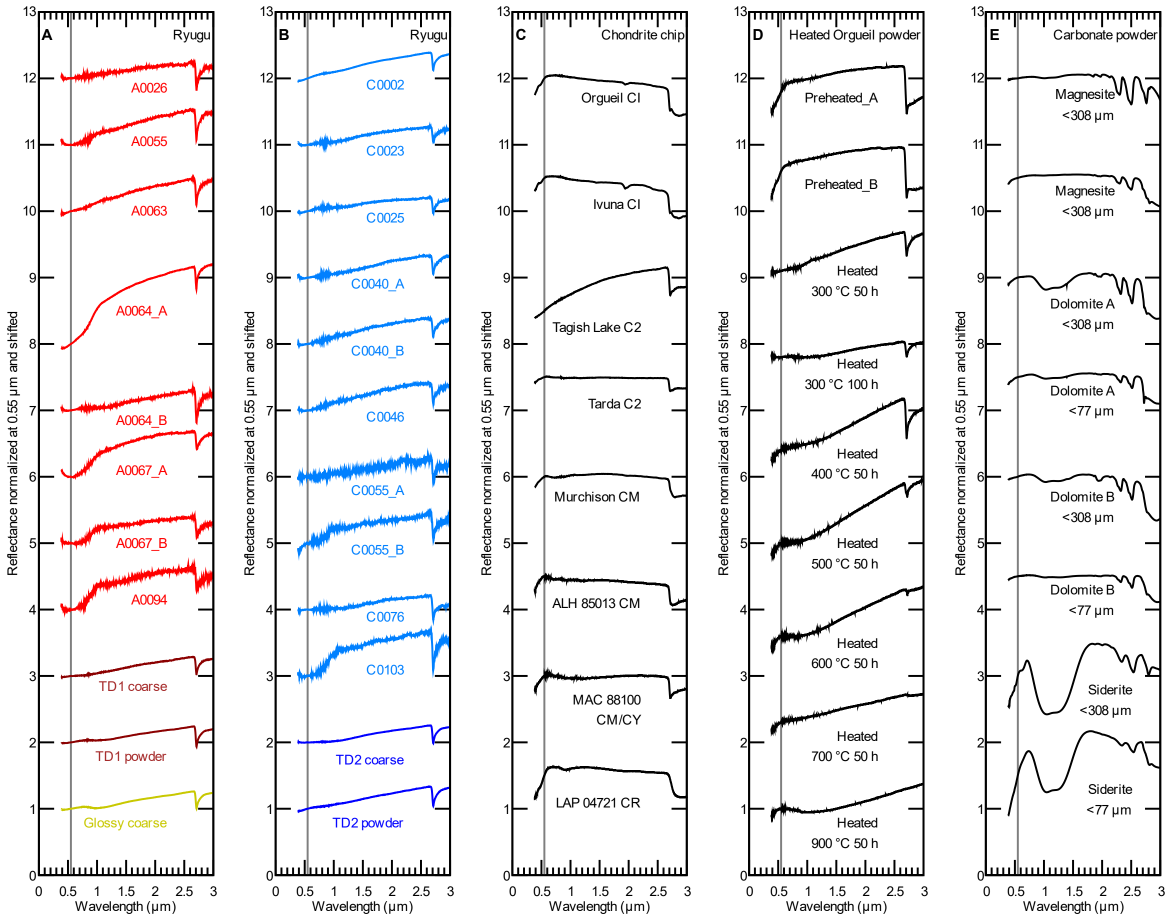


**Fig. S1. Vis-NIR reflectance spectra (0.4–3 µm).** (A and B) Ryugu samples, (C) carbonaceous chondrite chip samples, (D) experimentally-heated Orgueil powder samples, and (E) carbonate powder samples. All the spectra are normalized at 0.55 µm (v-band) and shifted arbitrarily. Gray lines indicate 0.55 µm. Some spectra exhibit a concave-up toward UV wavelengths probably due to specular reflection from a sample surface, whose intensities may depend on the sample orientation. Some Ryugu samples show a "shoulder" at 1 µm, or the turning point that connects the rapid reflectance increase from ~0.5 to 1 µm and the slight reflectance increase to NIR wavelengths. The presence of the 1-µm shoulder may also depend on the sample orientation because Ryugu grains A0064 and A0067 show 1-µm shoulder features with different intensities. Low signal levels at ~0.38–0.5 µm in wavelength may affect the spectral data, particularly resulting in an abrupt change in slope towards the UV region.

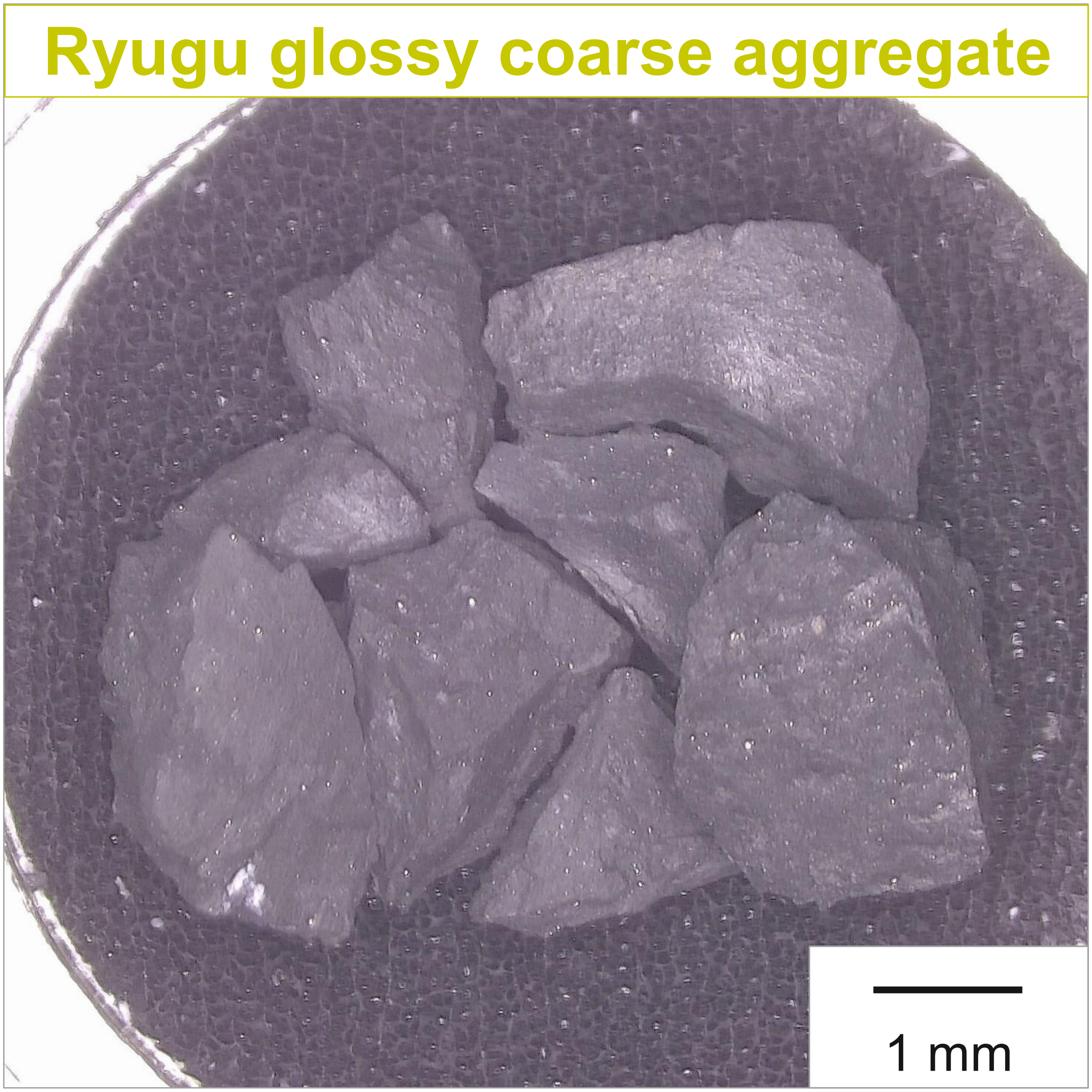


**Fig. S2. Microscopic images of Ryugu samples with a glossy and smooth surface.** The coarse aggregate includes A0064, A0067, A0094, C0025, C0033, C0061, C0076, and C0103, and is referred to as "Glossy coarse" in the main text.

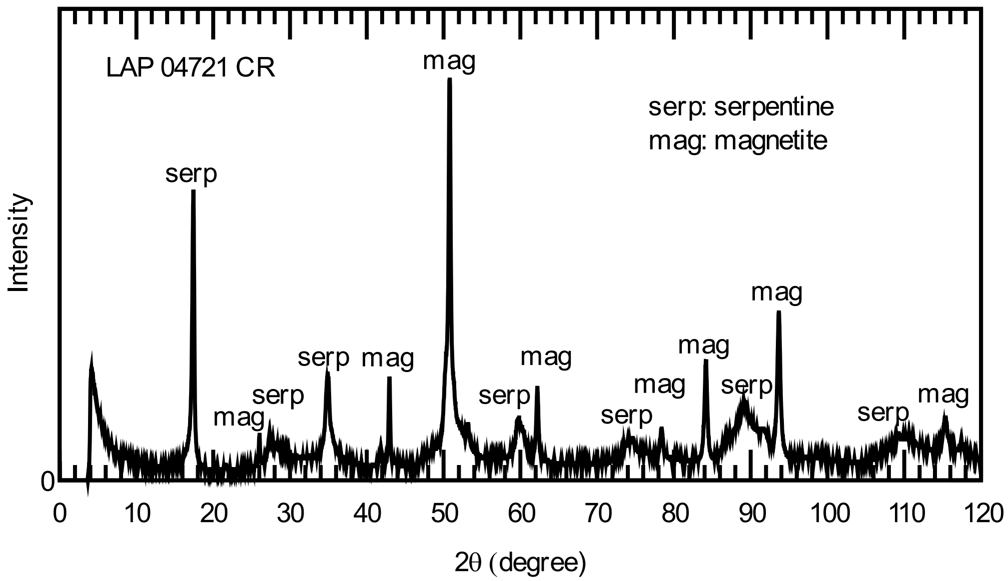


**Fig. S3. A synchrotron X-ray diffraction pattern of a matrix grain from LAP 04721 CR2 chondrite.** The matrix grain consists mainly of phyllosilicate (serpentine) and magnetite.

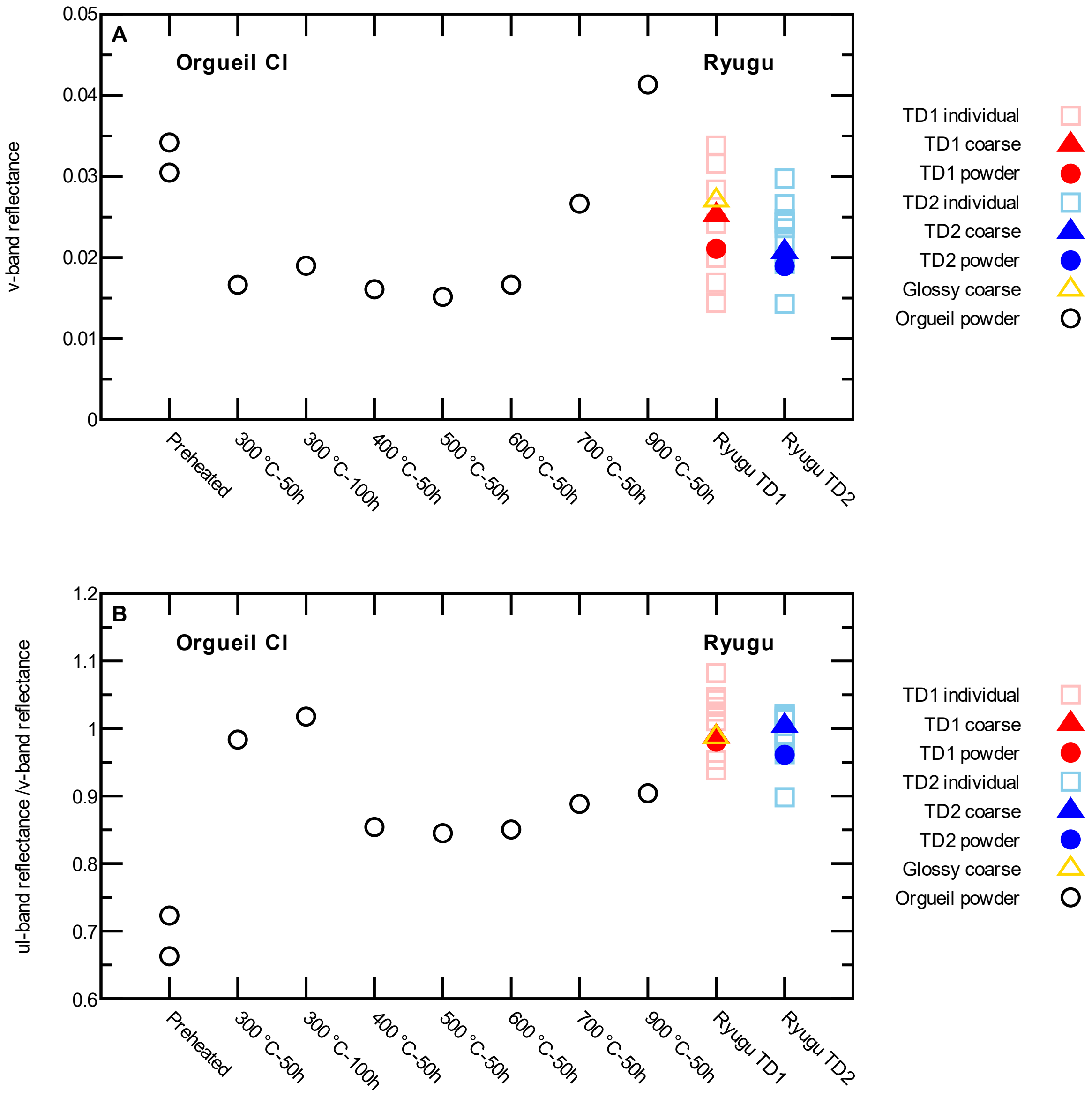


**Fig. S4. (A) The v-band reflectance and (B) the reflectance ratio of preheated and heated Orgueil powder and Ryugu TD1 and TD2 samples.** A low reflectance ratio of the ul-band (0.40 μm) reflectance to the v-band (0.48 μm) reflectance indicates a deep UV drop-off feature.

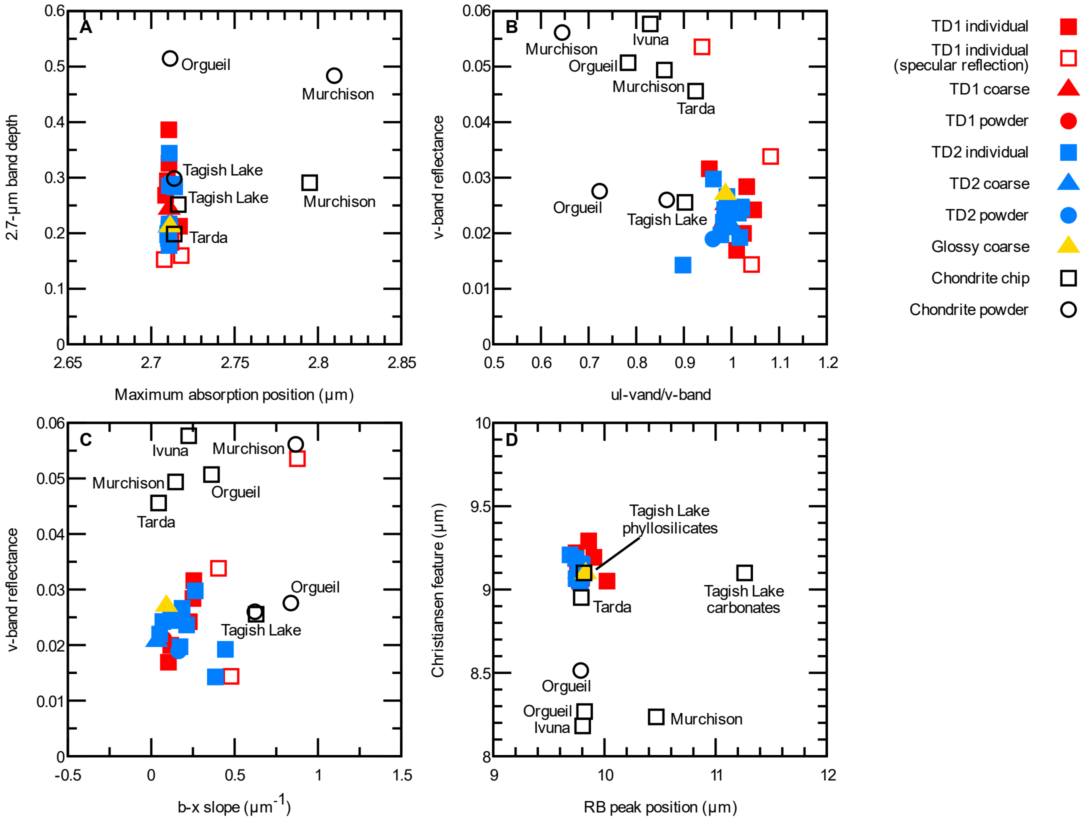


**Fig. S5. Spectral parameters of Ryugu samples compared to hydrated carbonaceous chondrites**. **(A)** The band depth and the maximum absorption position of the 2.7-µm absorption band. **(B)** The v-band reflectance and the reflectance ratio of ul-band and v-band. **(C)** The v-band reflectance and the spectral slope from b-band to x-band. **(D)** The peak position of Christiansen Feature and Reststrahlen Band of Si-O stretching.

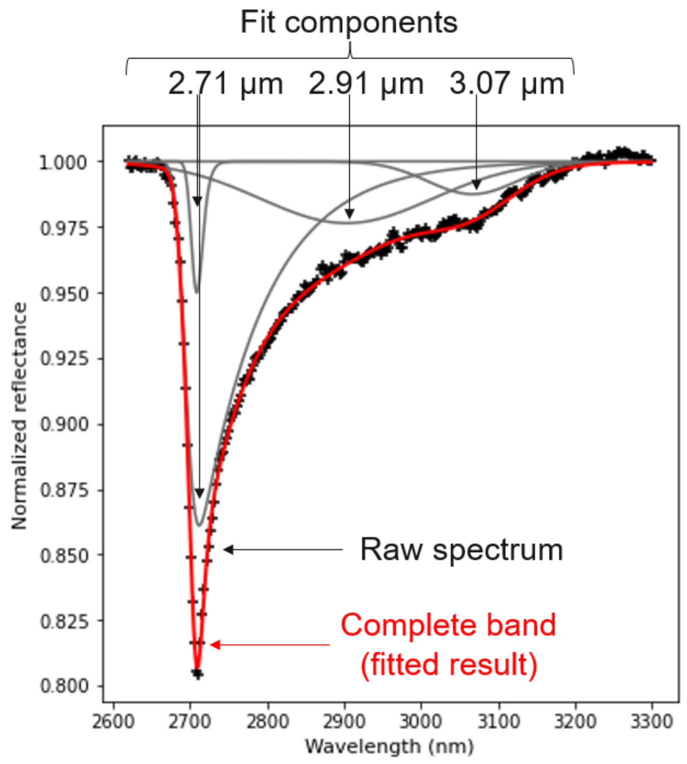


**Fig. S6**. **An EMG fitted result of the 2.7-µm absorption band of the Ryugu grain C0002.** Four components are assigned and the maximum absorption positions of the components are located at 2.71, 2.91, and 3.07 µm.

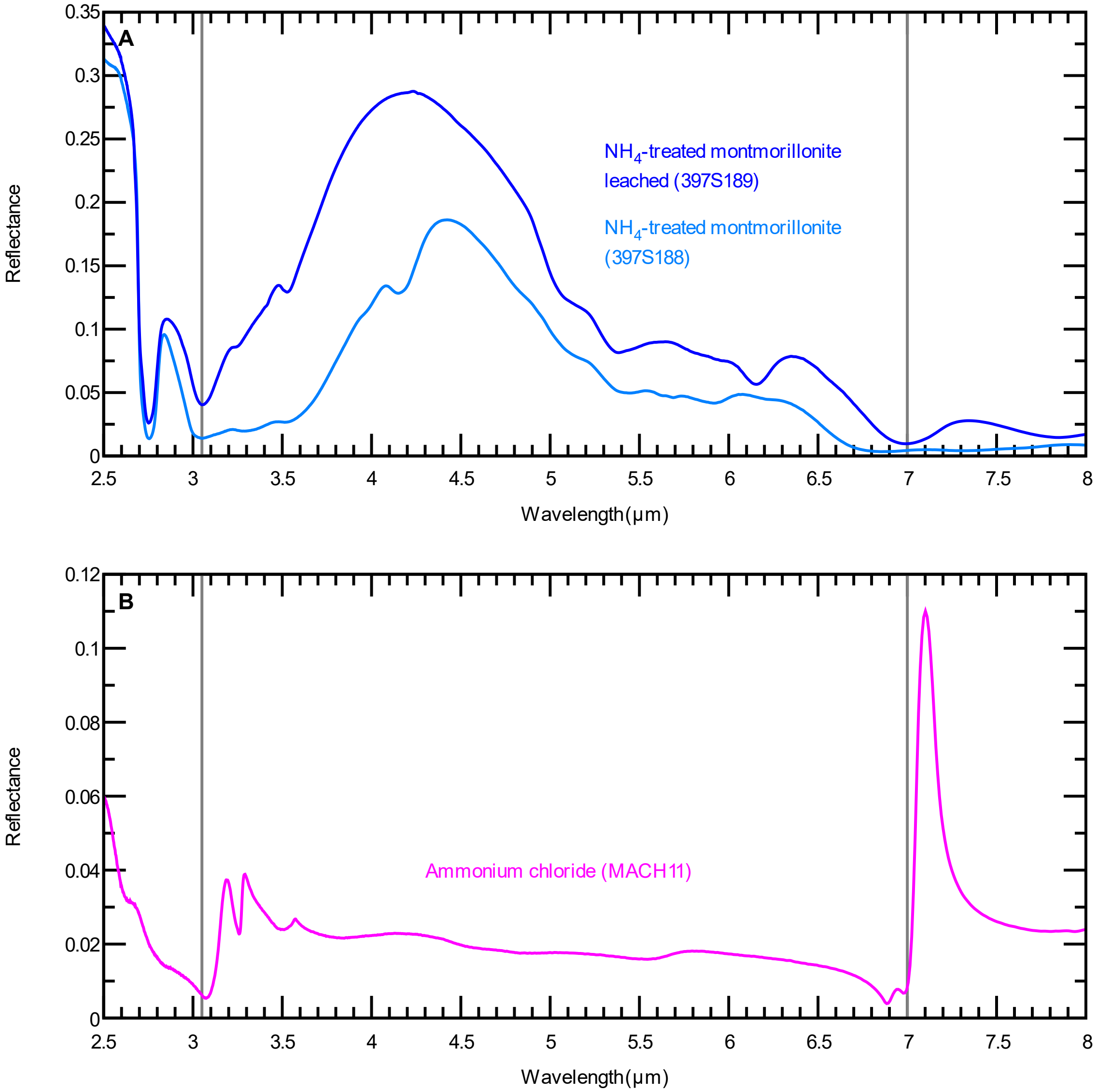


**Fig. S7**. **Infrared reflectance spectra of ammonium-bearing species.** (A) Ammonium-bearing phyllosilicates, and (B) ammonium chloride (*46*). The gray lines indicate 3.05 and 7.0 μm.

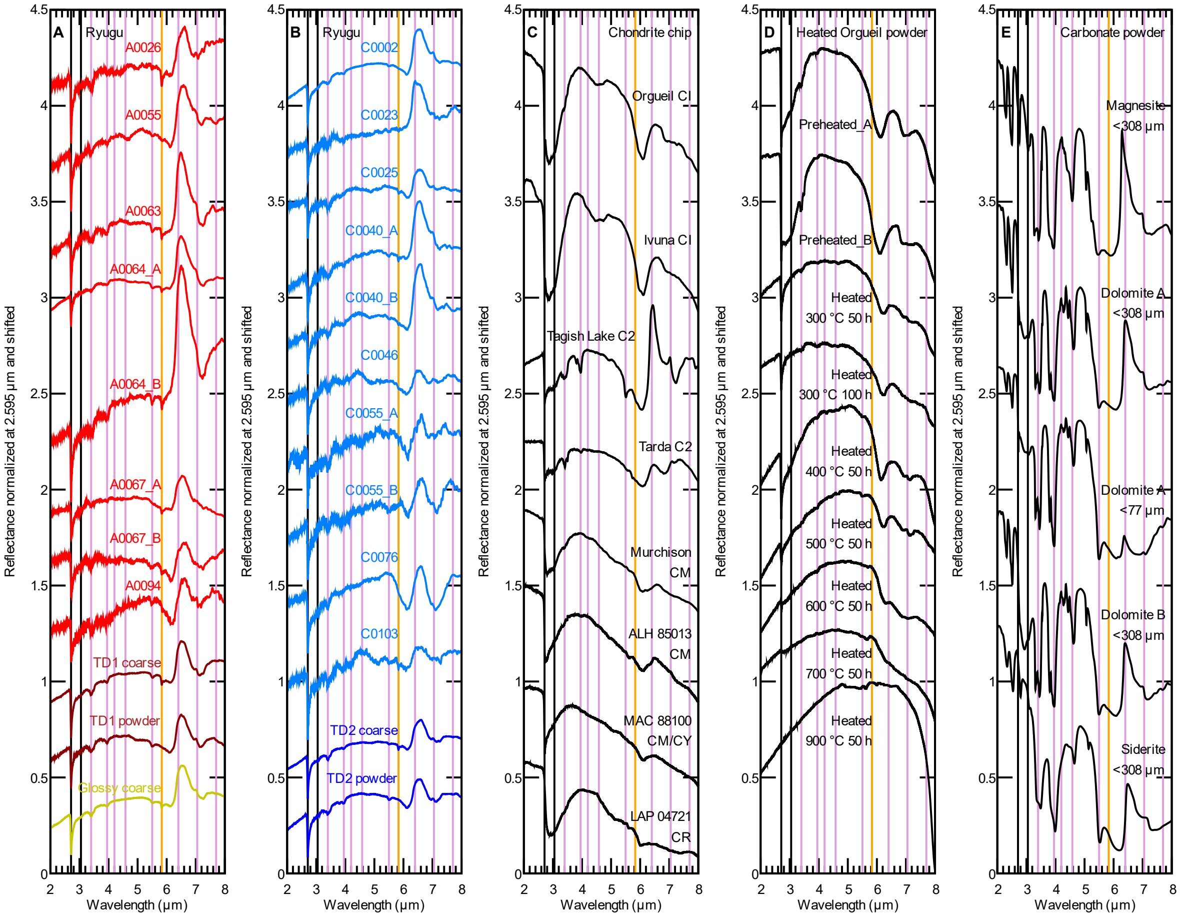


**Fig. S8. Infrared reflectance spectra (2–8 μm).** (A and B) Ryugu samples, (C) carbonaceous chondrite chip samples, (D) experimentally-heated Orgueil powder samples, and (E) carbonate powder samples. All the spectra are normalized at 2.595 μm and shifted arbitrarily. Gray lines indicate 2.71 and 3.05 μm. Purple lines indicate 3.40, 3.95, 4.20, 4.58, 5.50, 6.40, and 7.05 μm for carbonate features. Orange lines indicate 5.83 μm for the carbonyl peak.

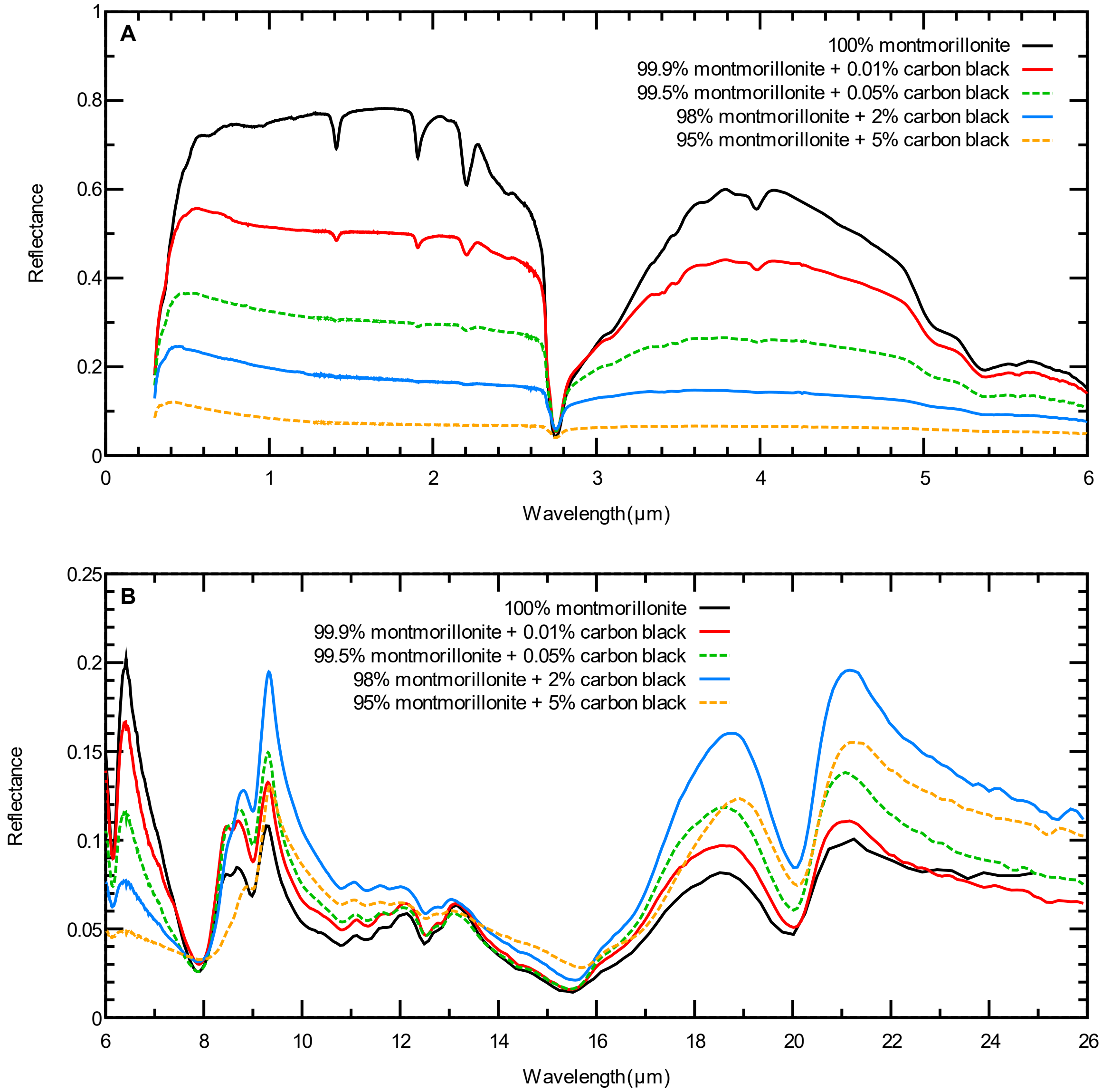


**Fig. S9. Reflectance spectra of the mixture of montmorillonite and carbon lamp black (A) at Vis-IR wavelengths and (B) at MIR wavelengths.** Reflectance and absorption features of montmorillonite become low and faint, respectively, by adding even less than a few percent of carbon black. Masking effects due to carbon black are more intense at Vis-NIR wavelengths than at MIR wavelengths. The spectral data can be found in Reflectance Experiment Laboratory (RELAB) database with IDs summarized in Table S5.

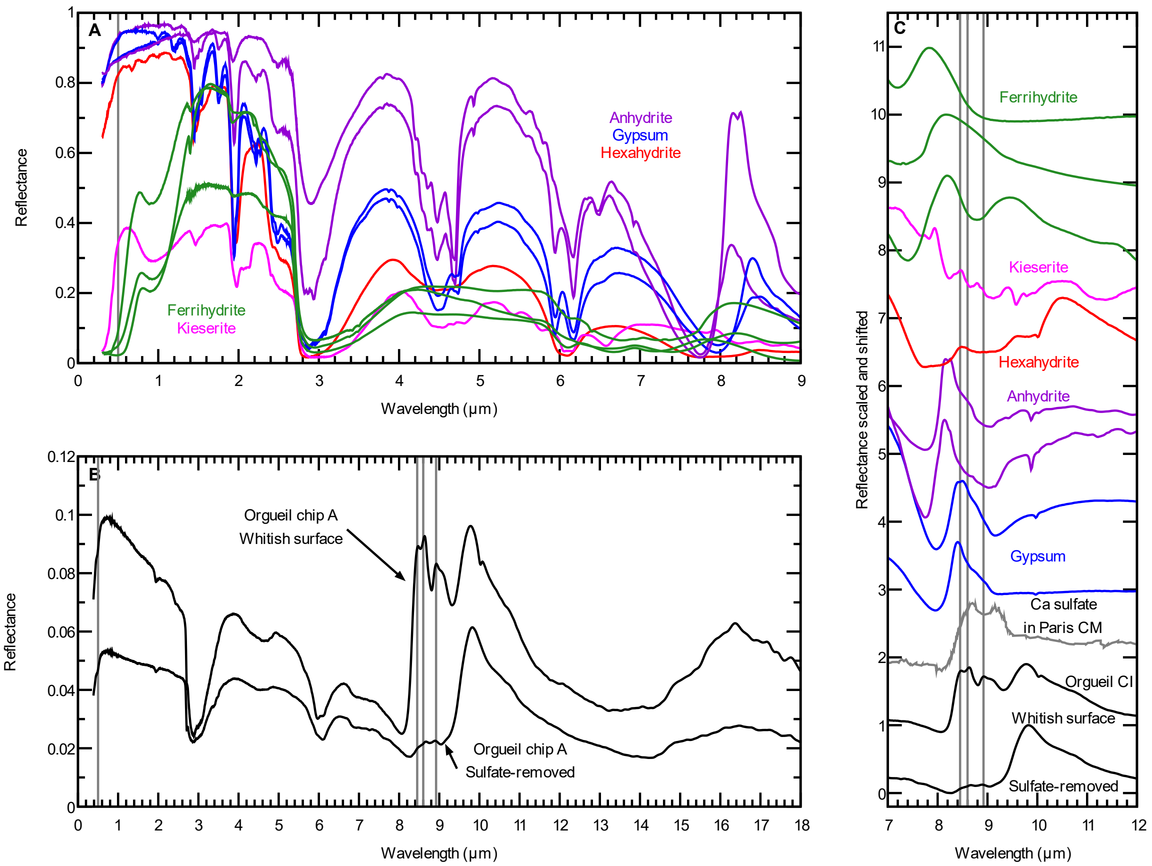


**Fig. S10. Reflectance spectra of sulfates, ferrihydrite, and Orgueil samples.** Spectral data of terrestrial sulfates and ferrihydrite were acquired for powder samples and cited from RELAB. (A) Vis-IR spectra of powder samples of ferrihydrite, Mg sulfates (kieserite and hexahydrite), and Ca sulfates (anhydrite and gypsum) (*49*). The grey line indicates 0.5 µm for a ferrihydrite feature. (B) Orgueil Vis-IR spectra measured from a whitish surface and a surface after the removal of whitish particles. Gray lines indicate 0.5 µm for a ferrihydrite feature and 8.45, 8.60, and 8.92 µm for sulfate peaks. (C) MIR spectra of ferrihydrite, Mg sulfates (kieserite and hexahydrite), Ca sulfates (anhydrite and gypsum), Ca sulfate in Paris (CM) (*58*), and a whitish surface and sulfate-removed surface of Orgueil (CI). Gray lines indicate 8.45, 8.60, and 8.92 µm for peaks of sulfates in Orgueil.

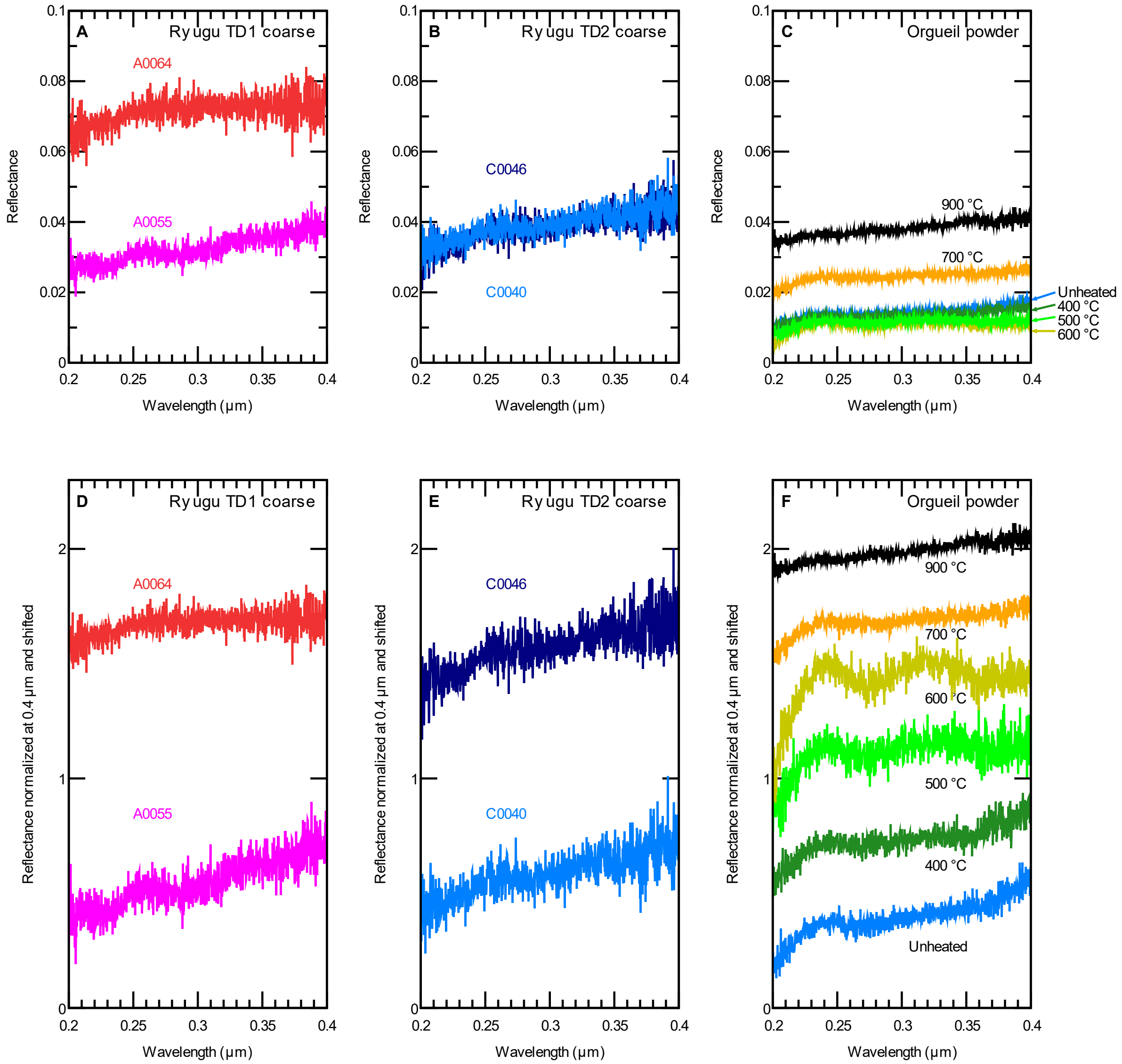


**Fig. S11. UV reflectance spectra of Ryugu samples and Orgueil CI powder samples heated experimentally**. (A) TD1 and (B) TD2 Ryugu coarse grains, and (C) experimentally-heated Orgueil CI powder samples. The spectra normalized at 0.39–0.40 μm of (D) TD1 and (E) TD2 Ryugu coarse grains and (F) experimentally-heated Orgueil CI powder samples. All spectra were measured after exposure to air.

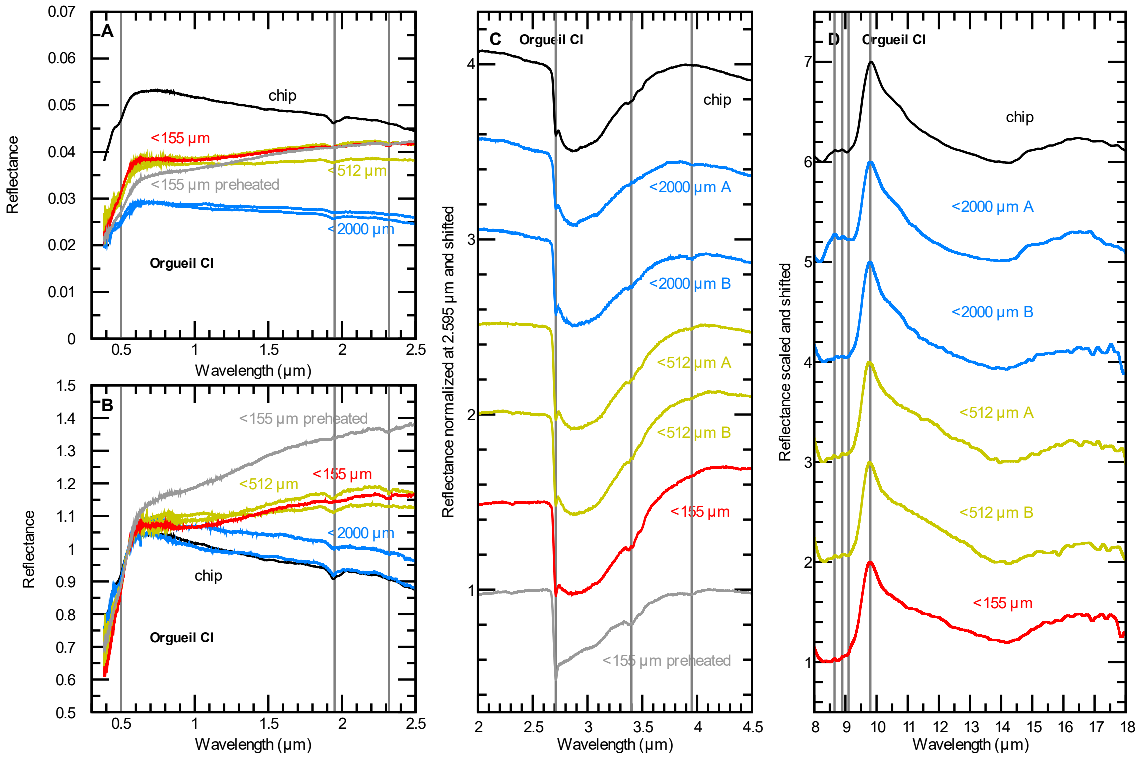


**Fig. S12. Reflectance spectra of Orgueil with various grain sizes.** (A) Vis-NIR reflectance spectra, (B) Vis-NIR reflectance spectra normalized at 0.55 μm, (C) Infrared (2–4 μm) reflectance spectra normalized at 2.595 μm, and (D) Mid-infrared (8–18 μm) spectra scaled. The chip sample exhibits the brightest and bluest spectrum at Vis-NIR wavelengths. Fine powder samples exhibit brighter and redder spectra at Vis-NIR wavelengths and deeper absorption features at 2.7 μm than coarse powder samples. Gray lines indicate 0.50 μm for Fe oxides/hydroxides (*39*), 1.95, 2.32, 8.65, and 8.90 μm for gypsum (*49*), 2.71μm for Mg-rich phyllosilicates (*33*), 3.40 μm for carbonates (*47*) and organics (*48*), 3.95 μm for carbonates (*47*), 9.10 μm for CF, and 9.80 μm for RB.

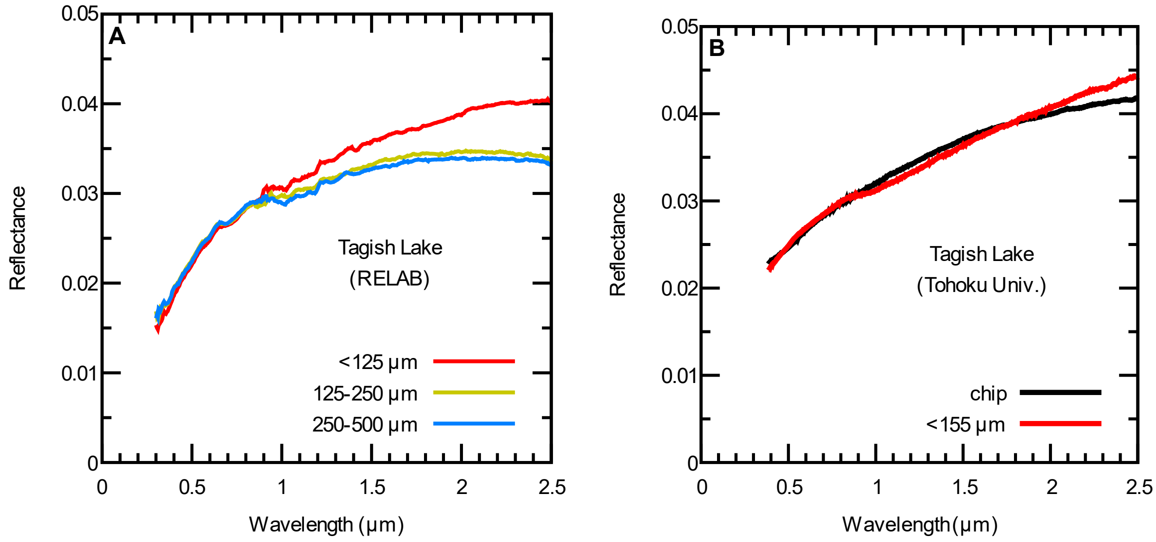


**Fig. S13. Vis-NIR reflectance spectra of Tagish Lake C2 chondrite with various grain sizes obtained (A) at RELAB and (B) Tohoku University.** No large Vis-NIR spectral variations are observed in the plots depending on the sample forms of Tagish Lake meteorite. The spectral data shown in (A) can be found in Reflectance Experiment Laboratory (RELAB) database with IDs summarized in Table S5.

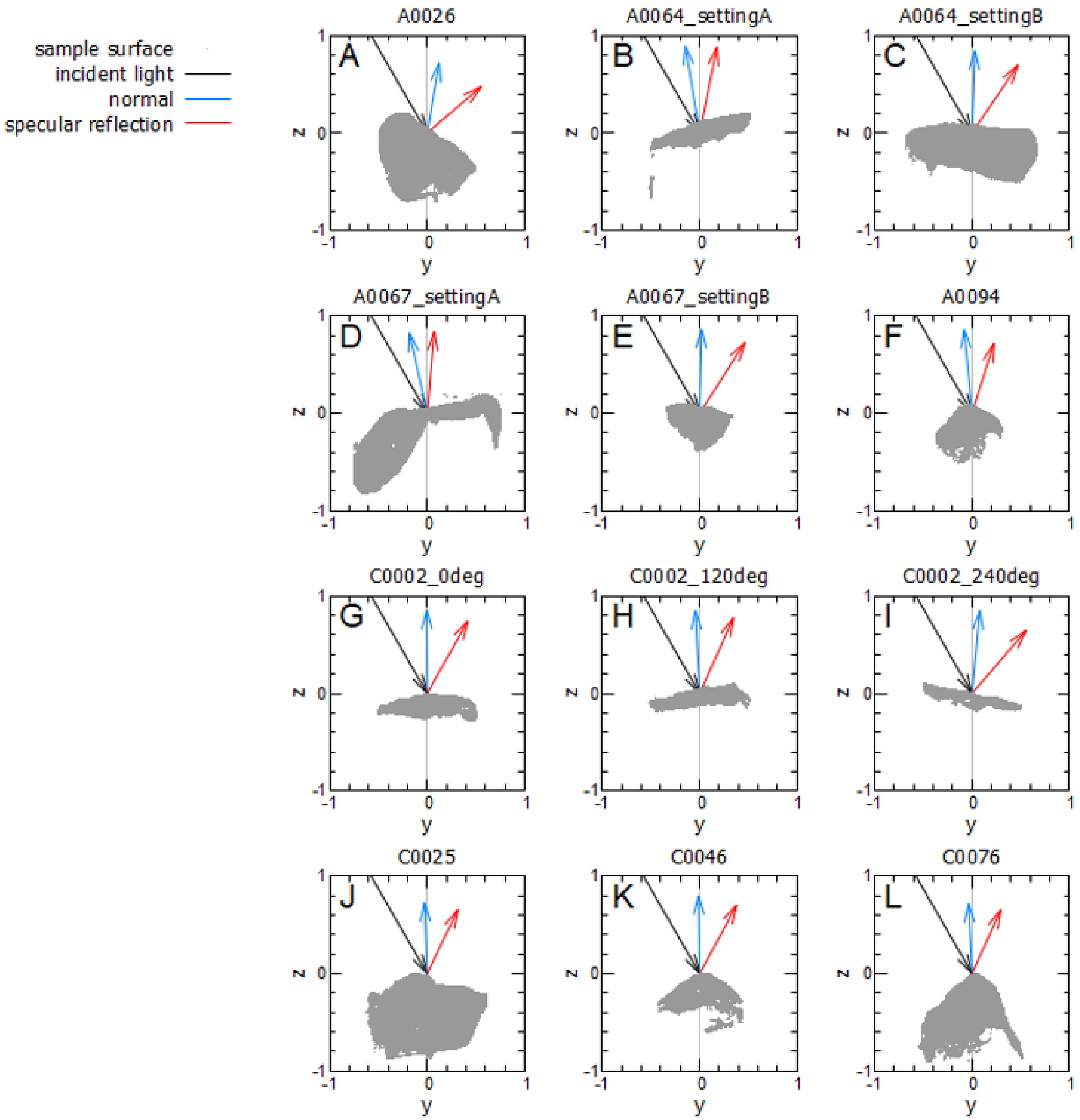


**Fig. S14. A cross-sectional view of each coarse sample during spectral measurements.** A plane parallel to the plane formed by the incidence and emergence angles is shown, and values perpendicular to this plane are projected onto this plane. A sample top surface is indicated by gray dots. The black, blue, and red arrows show the direction of the incident light, the mean value of the normal vector to each small area on the surface of the sample, and the specular reflection direction that is expected from the angle of incidence and the normal vector of the sample assuming the sample were a specular surface. The angle size of a specular reflection direction (a red arrow) is determined by the angle between the incidence angle (a black arrow) and the normal vector (a blue arrow). Each panel indicates an individual Ryugu grain set for a measurement. Sample sizes shown in the panels are scaled to fit in each panel. (A) A0026 with a rugged surface, (B) A0064 with a flat surface, (C) the same grain as (B) but in a different setting,

(D) A0067 with a flat surface, (E) the same grain as (D) but in a different setting, (F) A0094 with a flat surface, (G) C0002 with a rugged surface, (H) the same grain as (G) but rotated 120 degrees to the right about the z-axis, (I) the same grain as (G) but rotated 240 degrees to the right about the z-axis, (J) C0025 with a rugged surface, (K) C0046 with a rugged surface, and (L) C0076 with a rugged surface.

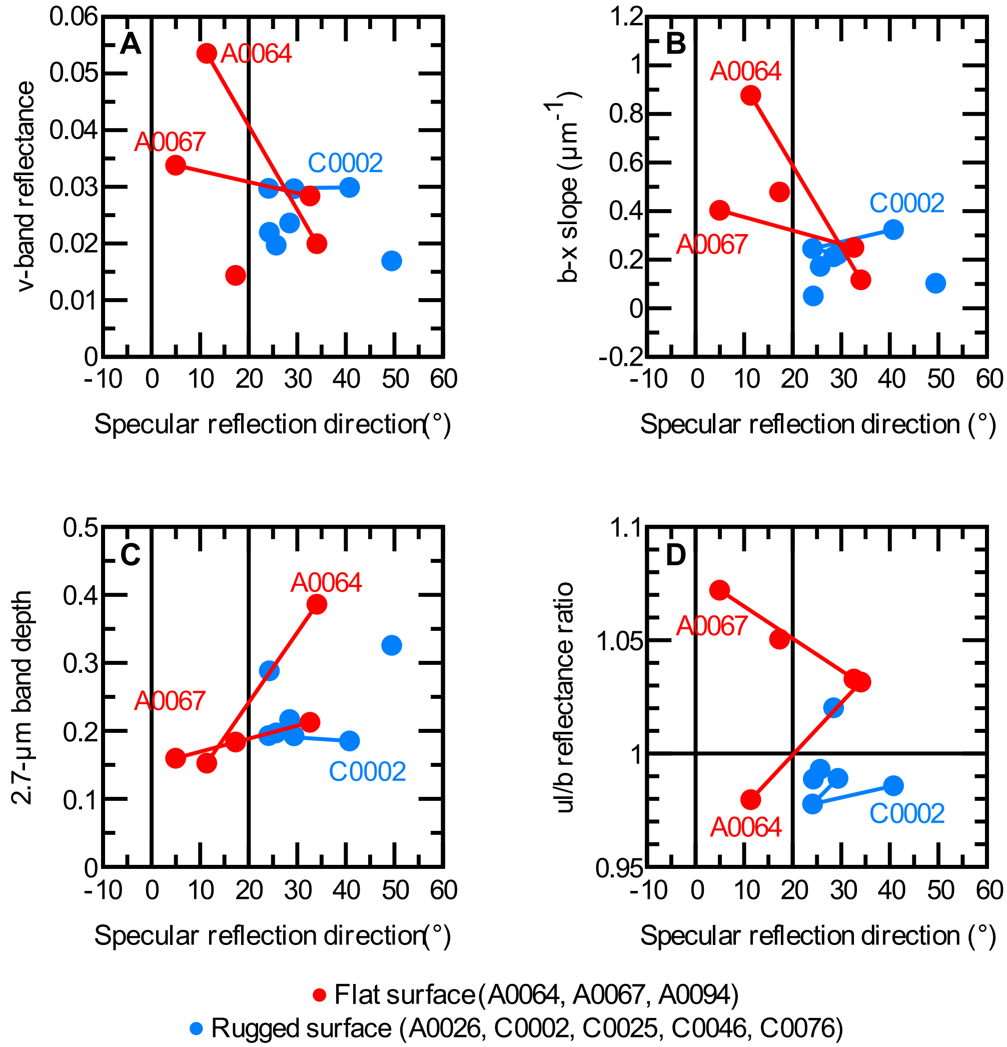


**Fig. S15. Spectral variations in relation to specular reflection direction.** (A) v-band reflectance, (B) b-x slope, (C) 2.7-µm band depth, and (D) the ul-band/b-band reflectance ratio as a function of expected angles of specular reflection direction based on the investigation as shown in Fig. S14. Red and blue dots indicate the spectral data obtained from a flat surface (A0064, A0067, and A0094) and a rugged surface (A0026, C0002, C0025, C0046, and C0076), respectively. The dots of the same grains measured with different orientations twice (i.e., A0064 and A0067) or three times (i.e., C0002) are connected by lines with each other.

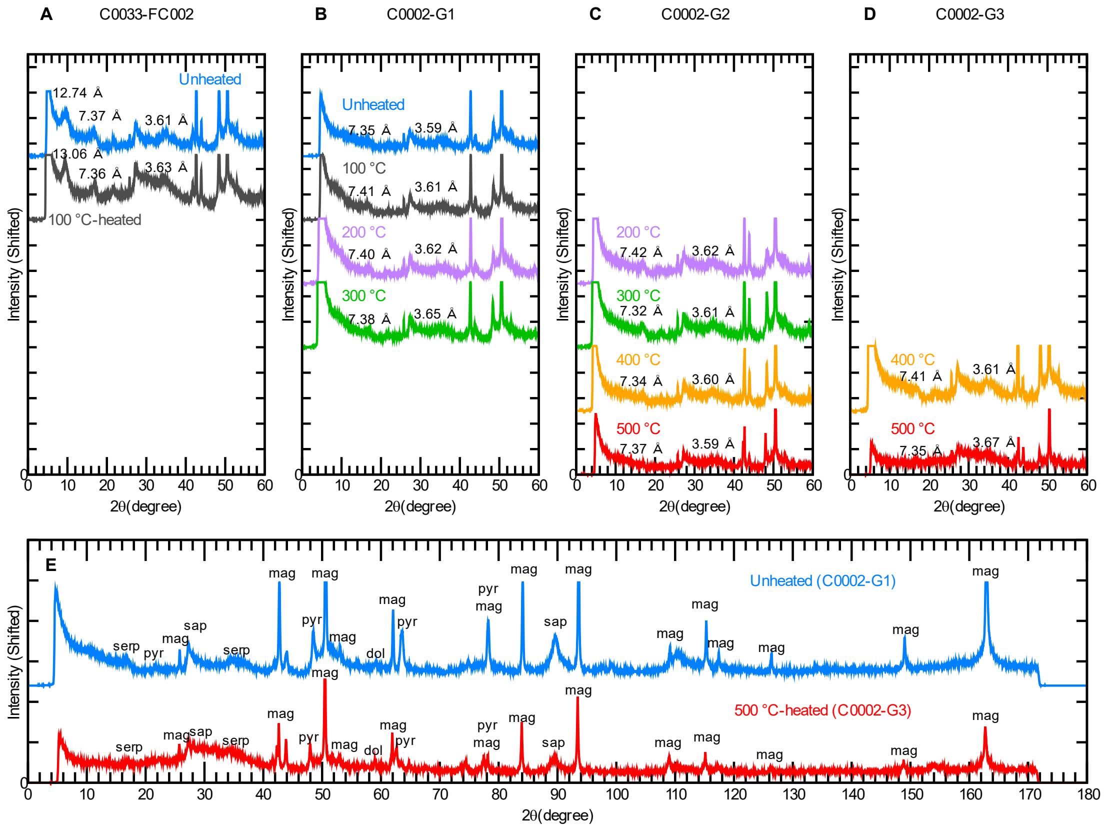


**Fig. S16. XRD patterns of experimentally-heated Ryugu small grains.** The lattice spacings of saponite and serpentine are indicated in the figures. Each mineral is abbreviated as: sap: saponite, serp: serpentine, mag: magnetite, pyr: pyrrhotite, dol: dolomite. After 500 °C-heating, diffraction peaks from phyllosilicates became weak, however, no other changes were observed. The sample IDs of the analyzed grains are (A) C0033-FC0002, (B) C0002-G1, (C) C0002-G2, and (D) C0002-G3. (E) XRD patterns of Ryugu samples unheated (blue) and step-wisely heated to 500 °C.

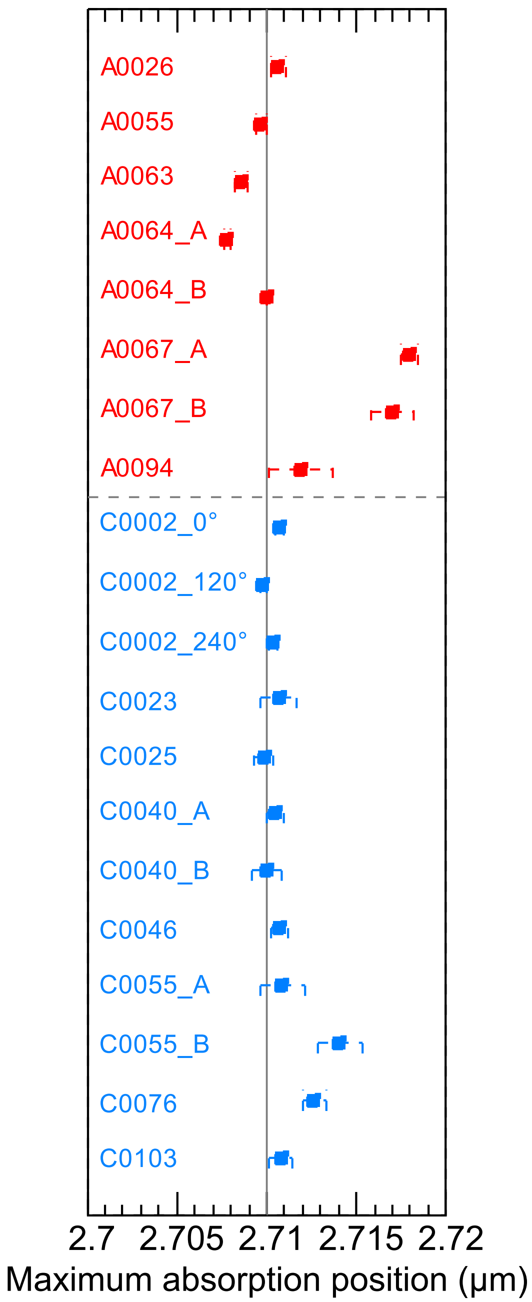


**Fig. S17. The maximum absorption positions of the 2.7-μm band of Ryugu samples determined by Exponentially Modified Gaussian fitting** (*42*)**.** The gray line indicates 2.710 μm. The Ryugu coarse grain A0067 has an absorption band at a longer wavelength than the other samples.

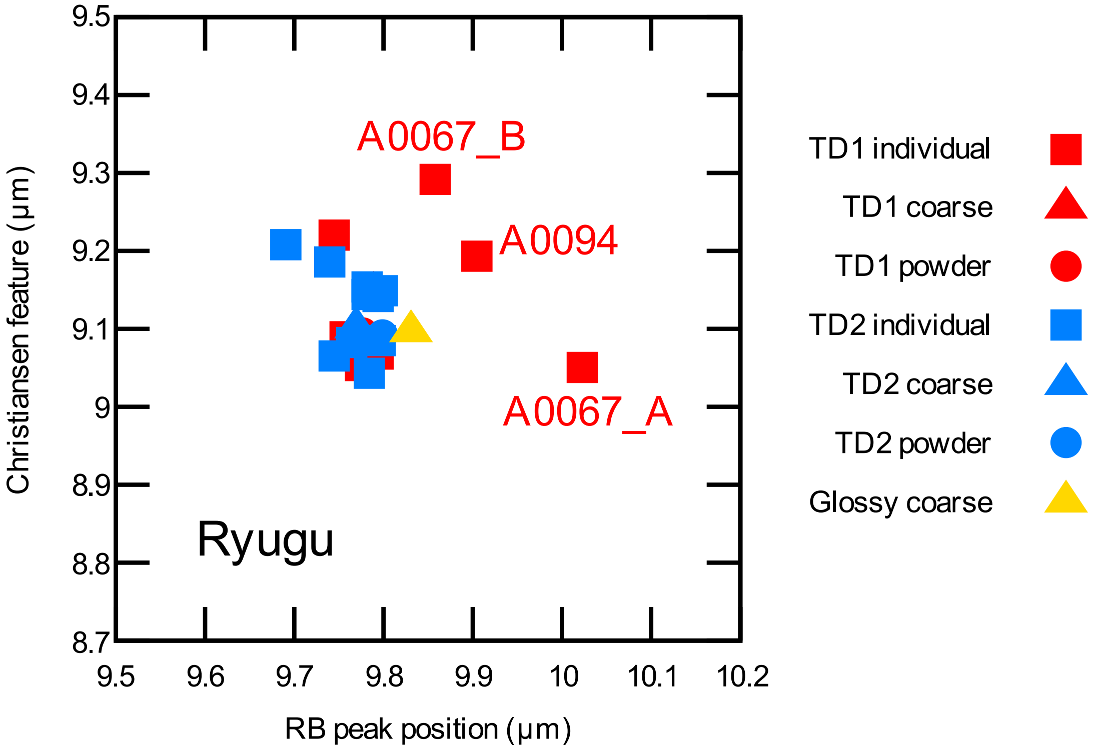


**Fig. S18. Positions of Christiansen feature and Reststrahlen Band of Ryugu samples.** TD1 and TD2 Ryugu samples are marked in red and blue, respectively. A magnified view of Fig. 9D. The Ryugu grains A0067 and A0094 have a thin amorphous layer covering the uppermost part of phyllosilicates, indicating space weathering (*15*). The shift of RB peak position towards longer wavelengths is consistent with the results of helium ion irradiation experiments on CI chondrites that simulated space weathering *(54*). Ryugu TD1 and TD2 samples were collected from the undisturbed area and the area containing excavated materials on the asteroid, respectively (*10*, *11*, *36*).

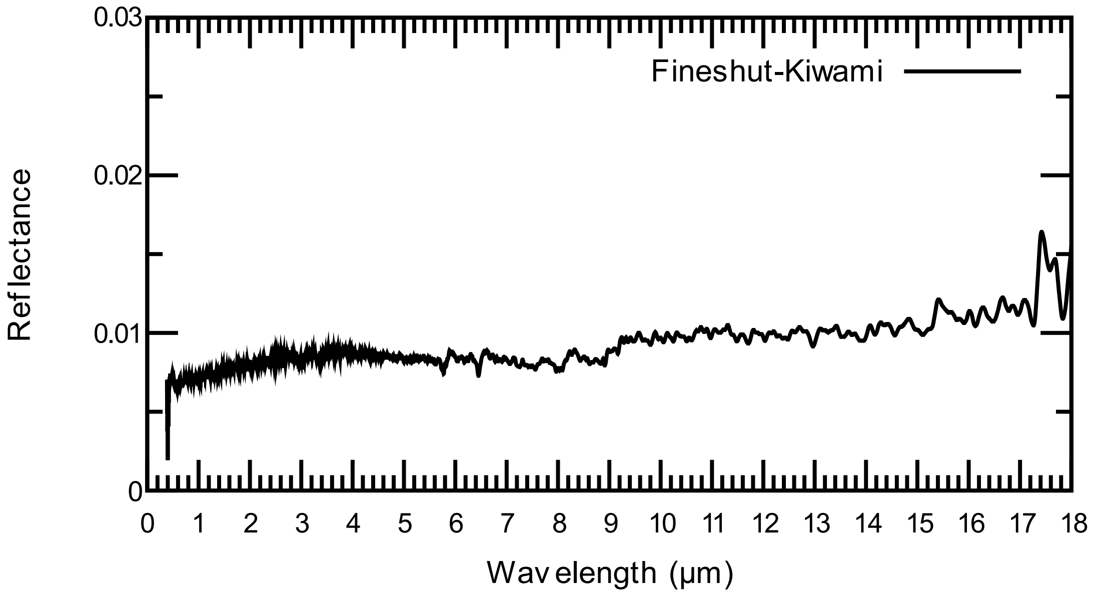


**Fig. S19. Reflectance spectra of a black sheet (Fineshut-Kiwami).** The spectrum was measured at incidence, emergence, and phase angles of 30°, 0°, and 30° in the principal plane, respectively by a bruker FTIR at Tohoku University.

**Table S1. The average spectral features of Ryugu grains**

| | All Ryugu samples | All Ryugu individual grains | The individual grains without intense specular reflection* | TD1 individual grains without intense specular reflection* | TD2 individual grains | Powder and coarse aggregates |
|---|---|---|---|---|---|---|
| v-band reflectance (%) | 2.5 ± 0.8 | 2.5 ± 0.9 | 2.3 ± 0.5 | 2.4 ± 0.5 | 2.3 ± 0.4 | 2.3 ± 0.4 |
| b-x slope ($\mu m^{-1}$) | 0.20 ± 0.18 | 0.26 ± 0.19 | 0.20 ± 0.11 | 0.19 ± 0.07 | 0.20 ± 0.12 | 0.09 ± 0.06 |
| 2.7-µm band depth (%) | 23 ± 6 | 24 ± 7 | 26 ± 6 | 30 ± 6 | 24 ± 5 | 22 ± 3 |

* Excluding A0064_settingA, A0067_settingA, and A0094.

The errors are given as one sigma.

**Table S2. Matrix mineralogy of experimentally-heated Orgueil**

| | Minerals* | | | | | | | | | | Heat stage† |
|---|---|---|---|---|---|---|---|---|---|---|---|
| | serp | sap | gyp | mag | pyr | dol | ol | wü | tae | ka | |
| Unheated | ✓ | ✓ | ✓ | ✓ | ✓ | ✓ | | | | | I |
| 300 °C 50 h | ✓ | ✓ | ✓‡ | ✓ | ✓ | ✓ | | | | | I |
| 300 °C 100 h | ✓ | ✓ | ✓‡ | ✓ | ✓ | ✓ | | | | | I |
| 300 °C 500 h | ✓ | ✓ | ✓‡ | ✓ | ✓ | ✓ | | | | | I |
| 400 °C 50 h | ✓ | ✓ | | ✓ | ✓ | ✓ | | | | | I or II |
| 500 °C 50 h | | ✓ | | ✓ | ✓§ | ✓ | | | | | II |
| 600 °C 50 h | | ✓ | | ✓ | ✓§ | | ✓ | | | | III |
| 700 °C 50 h | | | | | ✓ | | ✓ | ✓ | ✓ | | III or IV |
| 900 °C 50 h | | | | | | | ✓ | ✓ | ✓ | ✓ | IV |
| | serp | sap | gyp | mag | pyr | dol | ol | wu | tae | ka | |

*The presence of minerals is expressed as "✓". Each mineral is abbreviated as: serp: serpentine, sap: saponite, gyp: gypsum, mag: magnetite, pyr: pyrrhotite, dol: dolomite, ol: olivine, wü: wüstite, tae: taenite, ka: kamacite, and px: pyroxene.

†The heat stage is assigned based on the nomenclature for XRD patterns of naturally-heated carbonaceous chondrites proposed by (*23*).

‡All the data were obtained from a small particle after exposure to air, and gypsum in the 300 °C-heated samples are expected to have been hydrated by atmospheric interaction after heating and FT-IR measurements.

§With small peaks.

**Table S3. Configuration of the FT-IR system and measurements**

| Wavelength (μm) | ~0.38–0.6 | ~0.6–~1.1 | ~1.1–~2.6 | ~2.6–~8 | ~8–25 |
|---|---|---|---|---|---|
| Light source | Xenon | Halogen | Halogen | Globar | Globar |
| Detector | Si diode | Si diode | Mercury Cadmium Telluride | Mercury Cadmium Telluride | Mercury Cadmium Telluride |
| Spectral resolution ($cm^{-1}$) | 32 | 16 | 8 | 4 | 4 |
| Reflectance standard | Spectralon (99%, Labsphere) | Spectralon (99%, Labsphere) | Infragold (Labsphere) | Infragold (Labsphere) | Infragold (Labsphere) |
| Optical window for air-tight measurements | $CaF_2$ | $CaF_2$ | $CaF_2$ | $CaF_2$ | ZnSe |

**Table S4. Ryugu sample summary**

| Sample | Sample ID | Major axis length (mm) (*13*) | Sample weight (mg) (*13*) | Error (*13*) | The presence of a flat surface | References for spectral data |
|---|---|---|---|---|---|---|
| TD1 Individual | A0026 | 3.21 | 5.7 | 0.14 | | |
| | A0055 | 2.41 | 6.21 | <0.01 | | |
| | A0063 | 2.65 | 3.76 | 0.12 | | |
| | A0064_settingA and B | 2.74 | 6.55 | <0.01 | Yes | |
| | A0067_setting A and B | 3.14 | 3.46 | <0.01 | Yes | |
| | A0094 | 2.03 | 1.6 | <0.01 | Yes | |
| TD1 powder | A0106 powder | | 38.4 | <0.01 | | (*13*) |
| TD1 coarse (7 grains) | A0026, A0055, A0058, A0063, A0064, A0067, and A0094 | | | | | (*13*) |
| TD2 Individual | C0002_rotated A, B, and C | 8.39 | 94.1 | <0.01 | | |
| | C0023 | 2.86 | 5.03 | <0.01 | | |
| | C0025 | 2.93 | 5.77 | <0.01 | | |
| | C0040_setting A and B | 3.30 | 4.42 | <0.01 | | |
| | C0046 | 1.93 | 2.14 | <0.01 | | |
| | C0055 | 2.09 | 0.84 | <0.01 | | |
| | C0076 | 2.55 | 4.85 | <0.01 | | |
| | C0103 | 1.91 | 1.45 | <0.01 | | |
| TD2 powder | C0107 | | 38.8 | <0.01 | | (*13*) |
| TD2 coarse (7 grains) | C0023, C0025, C0033, C0040, C0061, C0076, and C0103 | | | | | (*13*) |
| Glossy coarse (8 grains) | A0064, A0067, A0094, C0025, C0033, C0061, C0076 and C0103 | | | | | |

**Table S5. Description of meteorite and mineral samples included in this study**

| Type | Sample | Grain size | Note and References |
|---|---|---|---|
| CI | Orgueil (Sulfate-removed) | Chip<br><2000 µm<br><512 µm<br><155 µm | Orgueil Chip A |
| | Orgueil (Whitish surface) | Chip | Orgueil Chip A |
| CI | Ivuna | Chip | |
| C2-ungrouped | Tagish Lake | Chip | |
| | Tagish Lake | <155 µm | preheated (*13*) |
| | Tagish Lake | <125 µm | RELAB Spectral Database. Sample ID: MT-S1S-237-A, spectrum ID: C1MT237A. |
| | Tagish Lake | 125–250 µm | RELAB Spectral Database. Sample ID: MT-S1S-237-B, spectrum ID: C1MT237B. |
| | Tagish Lake | 250–500 µm | RELAB Spectral Database. Sample ID: MT-S1S-237-C, spectrum ID: C1MT237C. |
| C2-ungrouped | Tarda | Chip | |
| CM2 | Murchison | Chip | |
| | Murchison | <155 µm | preheated, (*13*) |
| CM2 | ALH 85013 | Chip | |
| CM/CY | MAC 88100 | Chip | |
| CR2 | LAP 04721 | Chip | |
| | LAP 04721 | <155 µm | |
| CV | Allende | Chip | (*40*) |
| CO | Moss | Chip | (*40*) |
| Experimentally-heated CI | Orgueil preheated A | <155 µm | Orgueil chip A, (*13*) |
| | Orgueil preheated B | <155 µm | Orgueil chip B. |
| | Orgueil 300 ◦C for 50 hours | <155 µm<br>Coarse aggregate | Orgueil chip B. |
| | Orgueil 300 ◦C for 100 hours | <155 µm | Orgueil chip B. |
| | Orgueil 300 ◦C for 500 hours | Coarse aggregate | Orgueil chip B. |
| | Orgueil 400 ◦C for 50 hours | <155 µm | Orgueil chip A. |
| | Orgueil 500 ◦C for 50 hours | <155 µm | Orgueil chip A. |
| | Orgueil 600 ◦C for 50 hours | <155 µm | Orgueil chip A. |

(continued)

| **Type** | **Sample** | **Grain size** | **References** |
|---|---|---|---|
| | Orgueil 700 ◦C for 50 hours | <155 μm | Orgueil chip A. |
| | Orgueil 900 ◦C for 50 hours | <155 μm | Orgueil chip A. |
| Carbonates | Magnesite | <308 μm<br><77 μm | Natural |
| | Dolomite A | <308 μm<br><77 μm | From Ibaraki, Japan, green-colored crystal. |
| | Dolomite B | <308 μm<br><77 μm | From Morocco, red-colored crystal. |
| | Siderite | <308 μm<br><77 μm | From Portugal. |
| Ammonium-bearing | $NH_4$-treated montmorillonite (SWy-1 + $NH_4$) | Particulate | RELAB Spectral Database. Sample ID: JB-JLB-188, spectrum ID: 397S188. |
| | $NH_4$-treated montmorillonite leached (Wy-1 + $NH_4$) | Particulate | RELAB Spectral Database. Sample ID: JB-JLB-189, spectrum ID: 397S189. |
| | Ammonium chloride | Particulate | RELAB Spectral Database. Sample ID: CH-JLB-011, spectrum ID: MACH11. |
| Mixture | SWy-1 montmorillonite | <125 μm | RELAB Spectral Database. Sample ID: JB-CMP-013, spectrum ID: 1290F13. |
| | 99.9% SWy, 0.1% Carbon | <125 μm | RELAB Spectral Database. Sample ID: JB-CMP-021, spectrum ID: 1191F21. |
| | 99.5% SWy, 0.5% Carbon | <125 μm | RELAB Spectral Database. Sample ID: JB-CMP-022, spectrum ID: 1191F22. |
| | 98% SWy, 2% Carbon | <125 μm | RELAB Spectral Database. Sample ID: JB-CMP-023, spectrum ID: 1191F23. |
| | 95% SWy, 5% Carbon | <125 μm | RELAB Spectral Database Sample ID: JB-CMP-024, spectrum ID: 1191F24. |
| Fe hydroxides | Ferrihydrite | <125 μm | RELAB Spectral Database Sample ID: JB-JLB-499-A, spectrum ID: BKR1JB499A. |
| | Ferrihydrite | Particulate | RELAB Spectral Database Sample ID: JB-JLB-045-A, spectrum ID: BKR1JB045A. |

(continued)

| Type | Sample | Grain size | References |
|---|---|---|---|
| | Ferrihydrite | <45 µm | RELAB Spectral Database Sample ID: JB-CMP-046, spectrum ID: BKR1JB046. |
| Sulfates | Kieserite | <45 µm | RELAB Spectral Database Sample ID: SF-EAC-055-A, spectrum ID: LASF55A. (*49*) |
| | Hexahydrite | <45 µm | RELAB Spectral Database Sample ID: SF-EAC-057-A, spectrum ID: LASF57A. (*49*) |
| | Anhydrite | <45 µm | RELAB Spectral Database Sample ID: SF-EAC-042-A, spectrum ID: LASF42A. (*49*) |
| | Anhydrite | <45 µm | RELAB Spectral Database Sample ID: EC-EAC-002, spectrum ID: LAEC02. (*49*) |
| | Gypsum | <45 µm | RELAB Spectral Database Sample ID: SF-EAC-041-A, spectrum ID: LASF41A. (*49*) |
| | Gypsum | <45 µm | RELAB Spectral Database Sample ID: EC-EAC-005, spectrum ID: LAEC05. (*49*) |
| | Ca sulfate in Paris CM | | (*58*) |

**Table S6. Summary of the experimentally-heated Orgueil samples investigated**

| Original specimens | Sample | FT-IR* Powder† | FT-IR Coarse aggregate | FT-IR Chip | UV Powder† | S-XRD | STXM/ XANES | Carbon contents |
|---|---|---|---|---|---|---|---|---|
| Chip A | Unheated | ✓‡ | ✓ | ✓§ | ✓ | ✓ | | ✓ |
| | Preheated | ✓ | | | | | | |
| | 400 °C 50 h | ✓ | | | ✓ | ✓ | | |
| | 500 °C 50 h | ✓ | | | ✓ | ✓ | | ✓ |
| | 600 °C 50 h | ✓ | | | ✓ | ✓ | | |
| | 700 °C 50 h | ✓ | | | ✓ | ✓ | | |
| | 900 °C 50 h | ✓ | | | ✓ | ✓ | | |
| Chip B | Unheated | | | | | ✓ | ✓ | |
| | Preheated | ✓ | ✓ | | | | | |
| | 300 °C 50 h | ✓ | ✓ | | | ✓ | ✓ | |
| | 300 °C 100 h | ✓ | | | | ✓ | | |
| | 300 °C 500 h | | ✓ | | | ✓ | | |

Analyses performed are indicated by "✓". Blank cells indicate not analyzed.

* The FT-IR measurements of reflectance spectra.

† The powder samples were sieved to a grain size of <155 μm.

‡ The grain size of <2000 and <512 μm were investigated.

§ The two surfaces were characterized: a surface of Orgueil chip A after removing white particles by hand and a sulfate-rich surface of Orgueil chip A.

**Table S7. Wavelength bands used for Visible spectral characterization**

| | ul | b | v | Na | w | x | p |
|---|---|---|---|---|---|---|---|
| Wavelength of the band center* (nm) | 397.5 | 479.8 | 548.9 | 589.9 | 700.1 | 857.3 | 945.1 |
| Effective band width† (nm) | 36.0 | 26.6 | 30.6 | 11.8 | 29.2 | 41.7 | 56.0 |
| Start point of the band (nm) | 384.9‡ | 466.2 | 532.6 | 581.9 | 685.3 | 835.4 | 916.7 |
| End point of the band | 414.5 | 490.8 | 562.2 | 594.2 | 712.3 | 877.3 | 970.8 |

* Effective wavelength of the ONC-T band filters with respect to solar spectrum (*6*)

† Effective band width of the ONC-T band filters (*6*)

‡ The starting wavelength of the ul-band defined in this study is at a longer wavelength than that of the ONC-T band filters due to the wavelength limits of the FT-IR measurement.

## REFERENCES AND NOTES

1. A. S. Rivkin, E. S. Howell, F. Vilas, L. A. Lebofsky, Hydrated minerals on asteroids: The astronomical record. *Asteroids* **III**, 235–253 (2002).

2. F. E. DeMeo, B. Carry, Solar system evolution from compositional mapping of the asteroid belt. *Nature* **505**, 629–634 (2014).

3. P. A. Bland, M. E. Zolensky, G. K. Benedix, M. A. Sephton, Weathering of chondritic meteorites. *Meteorites and the Early Solar System* **II**, 853-867 (2006).

4. T. Yada, M. Abe, T. Okada, A. Nakato, K. Yogata, A. Miyazaki, K. Hatakeda, K. Kumagai, M. Nishimura, Y. Hitomi, H. Soejima, M. Yoshitake, A. Iwamae, S. Furuya, M. Uesugi, Y. Karouji, T. Usui, T. Hayashi, D. Yamamoto, R. Fukai, S. Sugita, Y. Cho, K. Yumoto, Y. Yabe, J. P. Bibring, C. Pilorget, V. Hamm, R. Brunetto, L. Riu, L. Lourit, D. Loizeau, G. Lequertier, A. Moussi-Soffys, S. Tachibana, H. Sawada, R. Okazaki, Y. Takano, K. Sakamoto, Y. N. Miura, H. Yano, T. R. Ireland, T. Yamada, M. Fujimoto, K. Kitazato, N. Namiki, M. Arakawa, N. Hirata, H. Yurimoto, T. Nakamura, T. Noguchi, H. Yabuta, H. Naraoka, M. Ito, E. Nakamura, K. Uesugi, K. Kobayashi, T. Michikami, H. Kikuchi, N. Hirata, Y. Ishihara, K. Matsumoto, H. Noda, R. Noguchi, Y. Shimaki, K. Shirai, K. Ogawa, K. Wada, H. Senshu, Y. Yamamoto, T. Morota, R. Honda, C. Honda, Y. Yokota, M. Matsuoka, N. Sakatani, E. Tatsumi, A. Miura, M. Yamada, A. Fujii, C. Hirose, S. Hosoda, H. Ikeda, T. Iwata, S. Kikuchi, Y. Mimasu, O. Mori, N. Ogawa, G. Ono, T. Shimada, S. Soldini, T. Takahashi, Y. Takei, H. Takeuchi, R. Tsukizaki, K. Yoshikawa, F. Terui, S. Nakazawa, S. Tanaka, T. Saiki, M. Yoshikawa, S. Watanabe, Y. Tsuda, Preliminary analysis of the Hayabusa2 samples returned from C-type asteroid Ryugu. *Nature Astronomy* **6**, 214–220 (2022).

5. S. Watanabe, M. Hirabayashi, N. Hirata, N. Hirata, R. Noguchi, Y. Shimaki, H. Ikeda, E. Tatsumi, M. Yoshikawa, S. Kikuchi, H. Yabuta, T. Nakamura, S. Tachibana, Y. Ishihara, T. Morota, K. Kitazato, N. Sakatani, K. Matsumoto, K. Wada, H. Senshu, C. Honda, T. Michikami, H. Takeuchi, T. Kouyama, R. Honda, S. Kameda, T. Fuse, H. Miyamoto, G. Komatsu, S. Sugita, T. Okada, N. Namiki, M. Arakawa, M. Ishiguro, M. Abe, R. Gaskell, E. Palmer, O. S. Barnouin, P. Michel, A. S. French, J. W. McMahon, D. J. Scheeres, P. A. Abell, Y. Yamamoto, S. Tanaka, K. Shirai, M. Matsuoka, M. Yamada, Y. Yokota,

H. Suzuki, K. Yoshioka, Y. Cho, S. Tanaka, N. Nishikawa, T. Sugiyama, H. Kikuchi, R. Hemmi, T. Yamaguchi, N. Ogawa, G. Ono, Y. Mimasu, K. Yoshikawa, T. Takahashi, Y. Takei, A. Fujii, C. Hirose, T. Iwata, M. Hayakawa, S. Hosoda, O. Mori, H. Sawada, T. Shimada, S. Soldini, H. Yano, R. Tsukizaki, M. Ozaki, Y. Iijima, K. Ogawa, M. Fujimoto, T.-M. Ho, A. Moussi, R. Jaumann, J.-P. Bibring, C. Krause, F. Terui, T. Saiki, S. Nakazawa, Y. Tsuda, Hayabusa2 arrives at the carbonaceous asteroid 162173 Ryugu—A spinning top–shaped rubble pile. *Science*. **364**, 268–272 (2019).

6. E. Tatsumi, T. Kouyama, H. Suzuki, M. Yamada, N. Sakatani, S. Kameda, Y. Yokota, R. Honda, T. Morota, K. Moroi, N. Tanabe, H. Kamiyoshihara, M. Ishida, K. Yoshioka, H. Sato, C. Honda, M. Hayakawa, K. Kitazato, H. Sawada, S. Sugita, Updated inflight calibration of Hayabusa2's optical navigation camera (ONC) for scientific observations during the cruise phase. *Icarus*. **325**, 153–195 (2019).

7. S. Sugita, R. Honda, T. Morota, S. Kameda, H. Sawada, E. Tatsumi, M. Yamada, C. Honda, Y. Yokota, T. Kouyama, N. Sakatani, K. Ogawa, H. Suzuki, T. Okada, N. Namiki, S. Tanaka, Y. Iijima, K. Yoshioka, M. Hayakawa, Y. Cho, M. Matsuoka, N. Hirata, N. Hirata, H. Miyamoto, D. Domingue, M. Hirabayashi, T. Nakamura, T. Hiroi, T. Michikami, P. Michel, R. L. Ballouz, O. S. Barnouin, C. M. Ernst, S. E. Schröder, H. Kikuchi, R. Hemmi, G. Komatsu, T. Fukuhara, M. Taguchi, T. Arai, H. Senshu, H. Demura, Y. Ogawa, Y. Shimaki, T. Sekiguchi, T. G. Müller, A. Hagermann, T. Mizuno, H. Noda, K. Matsumoto, R. Yamada, Y. Ishihara, H. Ikeda, H. Araki, K. Yamamoto, S. Abe, F. Yoshida, A. Higuchi, S. Sasaki, S. Oshigami, S. Tsuruta, K. Asari, S. Tazawa, M. Shizugami, J. Kimura, T. Otsubo, H. Yabuta, S. Hasegawa, M. Ishiguro, S. Tachibana, E. Palmer, R. Gaskell, L. Le Corre, R. Jaumann, K. Otto, N. Schmitz, P. A. Abell, M. A. Barucci, M. E. Zolensky, F. Vilas, F. Thuillet, C. Sugimoto, N. Takaki, Y. Suzuki, H. Kamiyoshihara, M. Okada, K. Nagata, M. Fujimoto, M. Yoshikawa, Y. Yamamoto, K. Shirai, R. Noguchi, N. Ogawa, F. Terui, S. Kikuchi, T. Yamaguchi, Y. Oki, Y. Takao, H. Takeuchi, G. Ono, Y. Mimasu, K. Yoshikawa, T. Takahashi, Y. Takei, A. Fujii, C. Hirose, S. Nakazawa, S. Hosoda, O. Mori, T. Shimada, S. Soldini, T. Iwata, M. Abe, H. Yano, R. Tsukizaki, M. Ozaki, K. Nishiyama, T. Saiki, S. Watanabe, Y. Tsuda, The geomorphology, color, and thermal properties of Ryugu: Implications for parent-body processes. *Science* **364**, 252 (2019).

8. K. Kitazato, R. E. Milliken, T. Iwata, M. Abe, M. Ohtake, S. Matsuura, T. Arai, Y. Nakauchi, T. Nakamura, M. Matsuoka, H. Senshu, N. Hirata, T. Hiroi, C. Pilorget, R. Brunetto, F. Poulet, L. Riu, J. P.

Bibring, D. Takir, D. L. Domingue, F. Vilas, M. A. Barucci, D. Perna, E. Palomba, A. Galiano, K. Tsumura, T. Osawa, M. Komatsu, A. Nakato, T. Arai, N. Takato, T. Matsunaga, Y. Takagi, K. Matsumoto, T. Kouyama, Y. Yokota, E. Tatsumi, N. Sakatani, Y. Yamamoto, T. Okada, S. Sugita, R. Honda, T. Morota, S. Kameda, H. Sawada, C. Honda, M. Yamada, H. Suzuki, K. Yoshioka, M. Hayakawa, K. Ogawa, Y. Cho, K. Shirai, Y. Shimaki, N. Hirata, A. Yamaguchi, N. Ogawa, F. Terui, T. Yamaguchi, Y. Takei, T. Saiki, S. Nakazawa, S. Tanaka, M. Yoshikawa, S. Watanabe, Y. Tsuda, The surface composition of asteroid 162173 Ryugu from Hayabusa2 near-infrared spectroscopy. *Science* **364**, 272–275 (2019).

9. T. Iwata, K. Kitazato, M. Abe, M. Ohtake, T. Arai, T. Arai, N. Hirata, T. Hiroi, C. Honda, N. Imae, M. Komatsu, T. Matsunaga, M. Matsuoka, S. Matsuura, T. Nakamura, A. Nakato, Y. Nakauchi, T. Osawa, H. Senshu, Y. Takagi, K. Tsumura, N. Takato, S.-i. Watanabe, M. A. Barucci, E. Palomba, M. Ozaki, NIRS3: The near infrared spectrometer on Hayabusa2. *Space Sci Rev.* **208**, 317–337 (2017).

10. T. Morota, S. Sugita, Y. Cho, M. Kanamaru, E. Tatsumi, N. Sakatani, R. Honda, N. Hirata, H. Kikuchi, M. Yamada, Y. Yokota, S. Kameda, M. Matsuoka, H. Sawada, C. Honda, T. Kouyama, K. Ogawa, H. Suzuki, K. Yoshioka, M. Hayakawa, N. Hirata, M. Hirabayashi, H. Miyamoto, T. Michikami, T. Hiroi, R. Hemmi, O. S. Barnouin, C. M. Ernst, K. Kitazato, T. Nakamura, L. Riu, H. Senshu, H. Kobayashi, S. Sasaki, G. Komatsu, N. Tanabe, Y. Fujii, T. Irie, M. Suemitsu, N. Takaki, C. Sugimoto, K. Yumoto, M. Ishida, H. Kato, K. Moroi, D. Domingue, P. Michel, C. Pilorget, T. Iwata, M. Abe, M. Ohtake, Y. Nakauchi, K. Tsumura, H. Yabuta, Y. Ishihara, R. Noguchi, K. Matsumoto, A. Miura, N. Namiki, S. Tachibana, M. Arakawa, H. Ikeda, K. Wada, T. Mizuno, C. Hirose, S. Hosoda, O. Mori, T. Shimada, S. Soldini, R. Tsukizaki, H. Yano, M. Ozaki, H. Takeuchi, Y. Yamamoto, T. Okada, Y. Shimaki, K. Shirai, Y. Iijima, H. Noda, S. Kikuchi, T. Yamaguchi, N. Ogawa, G. Ono, Y. Mimasu, K. Yoshikawa, T. Takahashi, Y. Takei, A. Fujii, S. Nakazawa, F. Terui, S. Tanaka, M. Yoshikawa, T. Saiki, S. Watanabe, Y. Tsuda, Sample collection from asteroid (162173) Ryugu by Hayabusa2: Implications for surface evolution. *Science* **368**, 654–659 (2020).

11. S. Tachibana, H. Sawada, R. Okazaki, Y. Takano, K. Sakamoto, Y. N. Miura, C. Okamoto, H. Yano, S. Yamanouchi, P. Michel, Y. Zhang, S. Schwartz, F. Thuillet, H. Yurimoto, T. Nakamura, T. Noguchi, H. Yabuta, H. Naraoka, A. Tsuchiyama, N. Imae, K. Kurosawa, A. M. Nakamura, K. Ogawa, S. Sugita, T. Morota, R. Honda, S. Kameda, E. Tatsumi, Y. Cho, K. Yoshioka, Y. Yokota, M. Hayakawa, M.

Matsuoka, N. Sakatani, M. Yamada, T. Kouyama, H. Suzuki, C. Honda, T. Yoshimitsu, T. Kubota, H. Demura, T. Yada, M. Nishimura, K. Yogata, A. Nakato, M. Yoshitake, A. I. Suzuki, S. Furuya, K. Hatakeda, A. Miyazaki, K. Kumagai, T. Okada, M. Abe, T. Usui, T. R. Ireland, M. Fujimoto, T. Yamada, M. Arakawa, H. C. Connolly, A. Fujii, S. Hasegawa, N. Hirata, N. Hirata, C. Hirose, S. Hosoda, Y. Iijima, H. Ikeda, M. Ishiguro, Y. Ishihara, T. Iwata, S. Kikuchi, K. Kitazato, D. S. Lauretta, G. Libourel, B. Marty, K. Matsumoto, T. Michikami, Y. Mimasu, A. Miura, O. Mori, K. Nakamura-Messenger, N. Namiki, A. N. Nguyen, L. R. Nittler, H. Noda, R. Noguchi, N. Ogawa, G. Ono, M. Ozaki, H. Senshu, T. Shimada, Y. Shimaki, K. Shirai, S. Soldini, T. Takahashi, Y. Takei, H. Takeuchi, R. Tsukizaki, K. Wada, Y. Yamamoto, K. Yoshikawa, K. Yumoto, M. E. Zolensky, S. Nakazawa, F. Terui, S. Tanaka, T. Saiki, M. Yoshikawa, S. Watanabe, Y. Tsuda, Pebbles and sand on asteroid (162173) Ryugu: In situ observation and particles returned to Earth. *Science* **375**, 1011–1016 (2022).

12. C. Pilorget, T. Okada, V. Hamm, R. Brunetto, T. Yada, D. Loizeau, L. Riu, T. Usui, A. Moussi-Soffys, K. Hatakeda, A. Nakato, K. Yogata, M. Abe, A. Aléon-Toppani, J. Carter, M. Chaigneau, B. Crane, B. Gondet, K. Kumagai, Y. Langevin, C. Lantz, T. Le Pivert-Jolivet, G. Lequertier, L. Lourit, A. Miyazaki, M. Nishimura, F. Poulet, M. Arakawa, N. Hirata, K. Kitazato, S. Nakazawa, N. Namiki, T. Saiki, S. Sugita, S. Tachibana, S. Tanaka, M. Yoshikawa, Y. Tsuda, S. Watanabe, J.-P. Bibring, First compositional analysis of Ryugu samples by the MicrOmega hyperspectral microscope. *Nat. Astron.* **6**, 221–225 (2022).

13. T. Nakamura, M. Matsumoto, K. Amano, Y. Enokido, M. E. Zolensky, T. Mikouchi, H. Genda, S. Tanaka, M. Y. Zolotov, K. Kurosawa, S. Wakita, R. Hyodo, H. Nagano, D. Nakashima, Y. Takahashi, Y. Fujioka, M. Kikuiri, E. Kagawa, M. Matsuoka, A. J. Brearley, A. Tsuchiyama, M. Uesugi, J. Matsuno, Y. Kimura, M. Sato, R. E. Milliken, E. Tatsumi, S. Sugita, T. Hiroi, K. Kitazato, D. Brownlee, D. J. Joswiak, M. Takahashi, K. Ninomiya, T. Takahashi, T. Osawa, K. Terada, F. E. Brenker, B. J. Tkalcec, L. Vincze, R. Brunetto, A. Aléon-Toppani, Q. H. S. Chan, M. Roskosz, J.-C. Viennet, P. Beck, E. E. Alp, T. Michikami, Y. Nagaashi, T. Tsuji, Y. Ino, J. Martinez, J. Han, A. Dolocan, R. J. Bodnar, M. Tanaka, H. Yoshida, K. Sugiyama, A. J. King, K. Fukushi, H. Suga, S. Yamashita, T. Kawai, K. Inoue, A. Nakato, T. Noguchi, F. Vilas, A. R. Hendrix, C. Jaramillo-Correa, D. L. Domingue, G. Dominguez, Z. Gainsforth, C. Engrand, J. Duprat, S. S. Russell, E. Bonato, C. Ma, T. Kawamoto, T. Wada, S. Watanabe, R. Endo, S. Enju, L. Riu, S. Rubino, P. Tack, S. Takeshita, Y. Takeichi, A. Takeuchi, A. Takigawa, D. Takir, T. Tanigaki, A. Taniguchi, K. Tsukamoto, T. Yagi, S. Yamada, K.

Yamamoto, Y. Yamashita, M. Yasutake, K. Uesugi, I. Umegaki, I. Chiu, T. Ishizaki, S. Okumura, E. Palomba, C. Pilorget, S. M. Potin, A. Alasli, S. Anada, Y. Araki, N. Sakatani, C. Schultz, O. Sekizawa, S. D. Sitzman, K. Sugiura, M. Sun, E. Dartois, E. De Pauw, Z. Dionnet, Z. Djouadi, G. Falkenberg, R. Fujita, T. Fukuma, I. R. Gearba, K. Hagiya, M. Y. Hu, T. Kato, T. Kawamura, M. Kimura, M. K. Kubo, F. Langenhorst, C. Lantz, B. Lavina, M. Lindner, J. Zhao, B. Vekemans, D. Baklouti, B. Bazi, F. Borondics, S. Nagasawa, G. Nishiyama, K. Nitta, J. Mathurin, T. Matsumoto, I. Mitsukawa, H. Miura, A. Miyake, Y. Miyake, H. Yurimoto, R. Okazaki, H. Yabuta, H. Naraoka, K. Sakamoto, S. Tachibana, H. C. Connolly Jr., D. S. Lauretta, M. Yoshitake, M. Yoshikawa, K. Yoshikawa, K. Yoshihara, Y. Yokota, K. Yogata, H. Yano, Y. Yamamoto, D. Yamamoto, M. Yamada, T. Yamada, T. Yada, K. Wada, T. Usui, R. Tsukizaki, F. Terui, H. Takeuchi, Y. Takei, A. Iwamae, H. Soejima, K. Shirai, Y. Shimaki, H. Senshu, H. Sawada, T. Saiki, M. Ozaki, G. Ono, T. Okada, N. Ogawa, K. Ogawa, R. Noguchi, H. Noda, M. Nishimura, N. Namiki, S. Nakazawa, T. Morota, A. Miyazaki, A. Miura, Y. Mimasu, K. Matsumoto, K. Kumagai, T. Kouyama, S. Kikuchi, K. Kawahara, S. Kameda, T. Iwata, Y. Ishihara, M. Ishiguro, H. Ikeda, S. Hosoda, R. Honda, C. Honda, Y. Hitomi, N. Hirata, N. Hirata, T. Hayashi, M. Hayakawa, K. Hatakeda, S. Furuya, R. Fukai, A. Fujii, Y. Cho, M. Arakawa, M. Abe, S. Watanabe, Y. Tsuda, Formation and evolution of carbonaceous asteroid Ryugu: Direct evidence from returned samples. *Science* **379**, 6634 (2022).

14. M. Matsuoka, E. Kagawa, K. Amano, T. Nakamura, E. Tatsumi, T. Osawa, T. Hiroi, R. Milliken, D. Domingue, D. Takir, R. Brunetto, A. Barucci, K. Kitazato, S. Sugita, Y. Fujioka, O. Sasaki, S. Kobayashi, T. Iwata, T. Morota, Y. Yokota, T. Kouyama, R. Honda, S. Kameda, Y. Cho, K. Yoshioka, H. Sawada, M. Hayakawa, N. Sakatani, M. Yamada, H. Suzuki, C. Honda, K. Ogawa, K. Shirai, C. Lantz, S. Rubino, H. Yurimoto, T. Noguchi, R. Okazaki, H. Yabuta, H. Naraoka, K. Sakamoto, S. Tachibana, T. Yada, M. Nishimura, A. Nakato, A. Miyazaki, K. Yogata, M. Abe, T. Okada, T. Usui, M. Yoshikawa, T. Saiki, S. Tanaka, F. Terui, S. Nakazawa, S. Watanabe, Y. Tsuda, Space weathering acts strongly on the uppermost surface of Ryugu. *Communications Earth & Environment.* **4,** 335 (2023).

15. T. Noguchi, T. Matsumoto, A. Miyake, Y. Igami, M. Haruta, H. Saito, S. Hata, Y. Seto, M. Miyahara, N. Tomioka, H. A. Ishii, J. P. Bradley, K. K. Ohtaki, E. Dobrică, H. Leroux, C. Le Guillou, D. Jacob, F. de la Peña, S. Laforet, M. Marinova, F. Langenhorst, D. Harries, P. Beck, T. H. V Phan, R. Rebois, N. M. Abreu, J. Gray, T. Zega, P.-M. Zanetta, M. S. Thompson, R. Stroud, K. Burgess, B. A. Cymes, J. C. Bridges, L. Hicks, M. R. Lee, L. Daly, P. A. Bland, M. E. Zolensky, D. R. Frank, J. Martinez, A.

Tsuchiyama, M. Yasutake, J. Matsuno, S. Okumura, I. Mitsukawa, K. Uesugi, M. Uesugi, A. Takeuchi, M. Sun, S. Enju, A. Takigawa, T. Michikami, T. Nakamura, M. Matsumoto, Y. Nakauchi, M. Abe, M. Arakawa, A. Fujii, M. Hayakawa, N. Hirata, N. Hirata, R. Honda, C. Honda, S. Hosoda, Y. Iijima, H. Ikeda, M. Ishiguro, Y. Ishihara, T. Iwata, K. Kawahara, S. Kikuchi, K. Kitazato, K. Matsumoto, M. Matsuoka, Y. Mimasu, A. Miura, T. Morota, S. Nakazawa, N. Namiki, H. Noda, R. Noguchi, N. Ogawa, K. Ogawa, T. Okada, C. Okamoto, G. Ono, M. Ozaki, T. Saiki, N. Sakatani, H. Sawada, H. Senshu, Y. Shimaki, K. Shirai, S. Sugita, Y. Takei, H. Takeuchi, S. Tanaka, E. Tatsumi, F. Terui, R. Tsukizaki, K. Wada, M. Yamada, T. Yamada, Y. Yamamoto, H. Yano, Y. Yokota, K. Yoshihara, M. Yoshikawa, K. Yoshikawa, R. Fukai, S. Furuya, K. Hatakeda, T. Hayashi, Y. Hitomi, K. Kumagai, A. Miyazaki, A. Nakato, M. Nishimura, H. Soejima, A. I. Suzuki, T. Usui, T. Yada, D. Yamamoto, K. Yogata, M. Yoshitake, H. C. Connolly, D. S. Lauretta, H. Yurimoto, K. Nagashima, N. Kawasaki, N. Sakamoto, R. Okazaki, H. Yabuta, H. Naraoka, K. Sakamoto, S. Tachibana, S. Watanabe, Y. Tsuda, A dehydrated space-weathered skin cloaking the hydrated interior of Ryugu. *Nat Astron.* **7**, 170–181 (2023).

16. T. Yokoyama, K. Nagashima, I. Nakai, E. D. Young, Y. Abe, J. Aléon, C. M. O. Alexander, S. Amari, Y. Amelin, K. Bajo, M. Bizzarro, A. Bouvier, R. W. Carlson, M. Chaussidon, B.-G. Choi, N. Dauphas, A. M. Davis, T. Di Rocco, W. Fujiya, R. Fukai, I. Gautam, M. K. Haba, Y. Hibiya, H. Hidaka, H. Homma, P. Hoppe, G. R. Huss, K. Ichida, T. Iizuka, T. R. Ireland, A. Ishikawa, M. Ito, S. Itoh, N. Kawasaki, N. T. Kita, K. Kitajima, T. Kleine, S. Komatani, A. N. Krot, M.-C. Liu, Y. Masuda, K. D. McKeegan, M. Morita, K. Motomura, F. Moynier, A. Nguyen, L. Nittler, M. Onose, A. Pack, C. Park, L. Piani, L. Qin, S. S. Russell, N. Sakamoto, M. Schönbächler, L. Tafla, H. Tang, K. Terada, Y. Terada, T. Usui, S. Wada, M. Wadhwa, R. J. Walker, K. Yamashita, Q.-Z. Yin, S. Yoneda, H. Yui, A.-C. Zhang, H. C. Connolly Jr., D. S. Lauretta, T. Nakamura, H. Naraoka, T. Noguchi, R. Okazaki, K. Sakamoto, H. Yabuta, M. Abe, M. Arakawa, A. Fujii, M. Hayakawa, N. Hirata, N. Hirata, R. Honda, C. Honda, S. Hosoda, Y. Iijima, H. Ikeda, M. Ishiguro, Y. Ishihara, T. Iwata, K. Kawahara, S. Kikuchi, K. Kitazato, K. Matsumoto, M. Matsuoka, T. Michikami, Y. Mimasu, A. Miura, T. Morota, S. Nakazawa, N. Namiki, H. Noda, R. Noguchi, N. Ogawa, K. Ogawa, T. Okada, C. Okamoto, G. Ono, M. Ozaki, T. Saiki, N. Sakatani, H. Sawada, H. Senshu, Y. Shimaki, K. Shirai, S. Sugita, Y. Takei, H. Takeuchi, S. Tanaka, E. Tatsumi, F. Terui, Y. Tsuda, R. Tsukizaki, K. Wada, S. Watanabe, M. Yamada, T. Yamada, Y. Yamamoto, H. Yano, Y. Yokota, K. Yoshihara, M. Yoshikawa, K. Yoshikawa, S. Furuya, K. Hatakeda, T. Hayashi, Y. Hitomi, K. Kumagai, A. Miyazaki, A. Nakato, M. Nishimura, H. Soejima, A.

Suzuki, T. Yada, D. Yamamoto, K. Yogata, M. Yoshitake, S. Tachibana, H. Yurimoto, Samples returned from the asteroid Ryugu are similar to Ivuna-type carbonaceous meteorites. *Science* **379**, eabn7850 (2022).

17. J. Akai, T-T-T diagram of serpentine and saponite, and estimation of metamorphic heating degree of Antarctic carbonaceous chondrites. *National institute of Polar Research.* **5**, 120–135 (1992).

18. W. Nozaki, T. Nakamura, T. Noguchi, Bulk mineralogical changes of hydrous micrometeorites during heating in the upper atmosphere at temperatures below 1000 °C. *Meteorit. Planet. Sci.* **41**, 1095–1114 (2006).

19. A. Nakato, T. Nakamura, F. Kitajima, T. Noguchi, Evaluation of dehydration mechanism during heating of hydrous asteroids based on mineralogical and chemical analysis of naturally and experimentally heated CM chondrites. *Earth, Planets and Space* **60**, 855–864 (2008).

20. L. Remusat, J. Y. Bonnet, S. Bernard, A. Buch, E. Quirico, Molecular and isotopic behavior of insoluble organic matter of the Orgueil meteorite upon heating. *Geochim. Cosmochim. Acta* **263**, 235–247 (2019).

21. T. Hiroi, C. M. Pieters, M. E. Zolensky, M. E. Lipschutz, Evidence of thermal metamorphism on the C, G, B, and F Asteroids. *Science* **261**, 1016–1018 (1993).

22. T. Hiroi, M. E. Zolensky, C. M. Pieters, M. E. Lipschutz, Thermal metamorphism of the C, G, B, and F asteroids seen from the 0.7 μm, 3 μm, and UV absorption strengths in comparison with carbonaceous chondrites. *Meteorit. Planet. Sci.* **31**, 321–327 (1996).

23. T. Nakamura, Post-hydration thermal metamorphism of carbonaceous chondrites. *J. Mineral. Petrol. Sci.* **100**, 260–272 (2005).

24. A. J. King, H. C. Bates, D. Krietsch, H. Busemann, P. L. Clay, P. F. Schofield, S. S. Russell, The Yamato-type (CY) carbonaceous chondrite group: Analogues for the surface of asteroid Ryugu? *Geochemistry.* **79**, 125531 (2019).

25. A. J. King, P. F. Schofield, S. S. Russell, Thermal alteration of CM carbonaceous chondrites: Mineralogical changes and metamorphic temperatures. *Geochim. Cosmochim. Acta* **298**, 167–190 (2021).

26. E. Quirico, L. Bonal, P. Beck, C. M. O. D. Alexander, H. Yabuta, T. Nakamura, A. Nakato, L. Flandinet, G. Montagnac, P. Schmitt-Kopplin, C. D. K. Herd, Prevalence and nature of heating processes in CM and C2-ungrouped chondrites as revealed by insoluble organic matter. *Geochim. Cosmochim. Acta* **241**, 17–37 (2018).

27. A. Garenne, P. Beck, G. Montes-Hernandez, R. Chiriac, F. Toche, E. Quirico, L. Bonal, B. Schmitt, The abundance and stability of "water" in type 1 and 2 carbonaceous chondrites (CI, CM and CR). *Geochim. Cosmochim. Acta* **137**, 93–112 (2014).

28. P. Beck, A. Garenne, E. Quirico, L. Bonal, G. Montes-Hernandez, F. Moynier, B. Schmitt, Transmission infrared spectra (2–25 μm) of carbonaceous chondrites (CI, CM, CV–CK, CR, C2 ungrouped): Mineralogy, water, and asteroidal processes. *Icarus.* **229**, 263–277 (2014).

29. A. Garenne, P. Beck, G. Montes-Hernandez, O. Brissaud, B. Schmitt, E. Quirico, L. Bonal, C. Beck, K. T. Howard, Bidirectional reflectance spectroscopy of carbonaceous chondrites: Implications for water quantification and primary composition. *Icarus.* **264**, 172–183 (2016).

30. S. Potin, P. Beck, F. Usui, L. Bonal, P. Vernazza, B. Schmitt, Style and intensity of hydration among C-complex asteroids: A comparison to desiccated carbonaceous chondrites. *Icarus.* **348**, 113826 (2020).

31. M. Matsuoka, T. Nakamura, N. Miyajima, T. Hiroi, N. Imae, A. Yamaguchi, Spectral and mineralogical alteration process of naturally-heated CM and CY chondrites. *Geochim. Cosmochim. Acta* **316**, 150–167 (2022).

32. D. Takir, J. P. Emery, H. Y. Mcsween, C. A. Hibbitts, R. N. Clark, N. Pearson, A. Wang, Nature and degree of aqueous alteration in CM and CI carbonaceous chondrites. *Meteorit. Planet. Sci.* **48**, 1618–1637 (2013).

33. P. Beck, E. Quirico, G. Montes-Hernandez, L. Bonal, J. Bollard, F. R. Orthous-Daunay, K. T. Howard, B. Schmitt, O. Brissaud, F. Deschamps, B. Wunder, S. Guillot, Hydrous mineralogy of CM and CI

chondrites from infrared spectroscopy and their relationship with low albedo asteroids. *Geochim. Cosmochim. Acta* **74**, 4881–4892 (2010).

34. D. Takir, K. R. Stockstill-Cahill, C. A. Hibbitts, Y. Nakauchi, 3-μm reflectance spectroscopy of carbonaceous chondrites under asteroid-like conditions. *Icarus.* **333**, 243–251 (2019).

35. C. Schultz, B. A. Anzures, R. E. Milliken, T. Hiroi, K. Robertson, Assessing the spatial variability of the ~3 μm OH/H2O absorption feature in CM2 carbonaceous chondrites. *Meteorit. Planet. Sci.* **58**, 170–194 (2023).

36. M. Arakawa, T. Saiki, K. Wada, K. Ogawa, T. Kadono, K. Shirai, H. Sawada, K. Ishibashi, R. Honda, N. Sakatani, Y. Iijima, C. Okamoto, H. Yano, Y. Takagi, M. Hayakawa, P. Michel, M. Jutzi, Y. Shimaki, S. Kimura, Y. Mimasu, T. Toda, H. Imamura, S. Nakazawa, H. Hayakawa, S. Sugita, T. Morota, S. Kameda, E. Tatsumi, Y. Cho, K. Yoshioka, Y. Yokota, M. Matsuoka, M. Yamada, T. Kouyama, C. Honda, Y. Tsuda, S. Watanabe, M. Yoshikawa, S. Tanaka, F. Terui, S. Kikuchi, T. Yamaguchi, N. Ogawa, G. Ono, K. Yoshikawa, T. Takahashi, Y. Takei, A. Fujii, H. Takeuchi, Y. Yamamoto, T. Okada, C. Hirose, S. Hosoda, O. Mori, T. Shimada, S. Soldini, R. Tsukizaki, T. Iwata, M. Ozaki, M. Abe, N. Namiki, K. Kitazato, S. Tachibana, H. Ikeda, N. Hirata, N. Hirata, R. Noguchi, A. Miura, An artificial impact on the asteroid (162173) Ryugu formed a crater in the gravity-dominated regime. *Science* **368**, 18 (2020).

37. E. A. Cloutis, P. Hudon, T. Hiroi, M. J. Gaffey, P. Mann, Spectral reflectance properties of carbonaceous chondrites: 2. CM chondrites. *Icarus*. **216**, 309–346 (2011).

38. A. R. Hendrix, F. Vilas, C-Complex Asteroids: UV-Visible Spectral Characteristics and Implications for Space Weathering Effects. *Geophys. Res. Lett.* **46**, 14307–14317 (2019).

39. E. A. Cloutis, T. Hiroi, M. J. Gaffey, C. M. O. D. Alexander, P. Mann, Spectral reflectance properties of carbonaceous chondrites: 1. CI chondrites. *Icarus*. **212**, 180–209 (2011).

40. C. B. Kiddell, E. A. Cloutis, B. R. Dagdick, J. M. Stromberg, D. M. Applin, J. P. Mann, Spectral Reflectance of Powder Coatings on Carbonaceous Chondrite Slabs: Implications for Asteroid Regolith Observations. *J Geophys Res Planets.* **123**, 2803–2840 (2018).

41. E. A. Cloutis, P. Hudon, T. Hiroi, M. J. Gaffey, Spectral reflectance properties of carbonaceous chondrites: 3. CR chondrites. *Icarus*. **217**, 389–407 (2012).

42. S. Potin, S. Manigand, P. Beck, C. Wolters, B. Schmitt, A model of the 3-μm hydration band with Exponentially Modified Gaussian (EMG) profiles: Application to hydrated chondrites and asteroids. *Icarus*. **343**, 113686 (2020).

43. K. M. Davis, M. Tomozawa, An infrared spectroscopic study of water-related species in silica glasses. *J. Non Cryst. Solids* **201**, 177–198 (1996).

44. M. C. DeSanctis, E. Ammannito, A. Raponi, S. Marchi, T. B. McCord, H. Y. McSween, F. Capaccioni, M. T. Capria, F. G. Carrozzo, M. Ciarniello, A. Longobardo, F. Tosi, S. Fonte, M. Formisano, A. Frigeri, M. Giardino, G. Magni, E. Palomba, D. Turrini, F. Zambon, J. P. Combe, W. Feldman, R. Jaumann, L. A. McFadden, C. M. Pieters, T. Prettyman, M. Toplis, C. A. Raymond, C. T. Russell, Ammoniated phyllosilicates with a likely outer Solar System origin on (1) Ceres. *Nature* **528**, 241–244 (2015).

45. O. Poch, I. Istiqomah, E. Quirico, P. Beck, B. Schmitt, P. Theulé, A. Faure, P. Hily-Blant, L. Bonal, A. Raponi, M. Ciarniello, B. Rousseau, S. Potin, O. Brissaud, L. Flandinet, G. Filacchione, A. Pommerol, N. Thomas, D. Kappel, V. Mennella, L. Moroz, V. Vinogradoff, G. Arnold, S. Erard, D. Bockelée-Morvan, C. Leyrat, F. Capaccioni, M. C. De Sanctis, A. Longobardo, F. Mancarella, E. Palomba, F. Tosi, Ammonium salts are a reservoir of nitrogen on a cometary nucleus and possibly on some asteroids. *Science* **367**, 332–340 (2020).

46. B. L. Berg, E. A. Cloutis, P. Beck, P. Vernazza, J. L. Bishop, D. Takir, V. Reddy, D. Applin, P. Mann, Reflectance spectroscopy (0.35–8 μm) of ammonium-bearing minerals and qualitative comparison to Ceres-like asteroids. *Icarus*. **265**, 218–237 (2016).

47. J. L. Bishop, S. J. King, M. D. Lane, A. J. Brown, B. Lafuente, T. Hiroi, R. Roberts, G. A. Swayze, J. F. Lin, M. Sánchez Román, Spectral properties of anhydrous carbonates and nitrates. *Earth and Space Science* **8**, e2021EA001844 (2021).

48. H. H. Kaplan, R. E. Milliken, C. M. O. D. Alexander, C. D. K. Herd, Reflectance spectroscopy of insoluble organic matter (IOM) and carbonaceous meteorites. *Meteorit. Planet. Sci.* **54**, 1051–1068 (2019).

49. E. A. Cloutis, F. C. Hawthorne, S. A. Mertzman, K. Krenn, M. A. Craig, D. Marcino, M. Methot, J. Strong, J. F. Mustard, D. L. Blaney, J. F. Bell, F. Vilas, Detection and discrimination of sulfate minerals using reflectance spectroscopy. *Icarus.* **184**, 121–157 (2006).

50. A. S. Wexler, Integrated Intensities of Absorption Bands in Infrared Spectroscopy. *Appl Spectrosc Rev.* **1**, 29–98 (1967).

51. G. Matrajt, G. M. M. Caro, E. Dartois, L. D'Hendecourt, D. Deboffle, J. Borg, FTIR analysis of the organics in IDPs: Comparison with the IR spectra of the diffuse interstellar medium. *Astron Astrophys.* **433**, 979–995 (2005).

52. H. Yabuta, G. D. Cody, C. Engrand, Y. Kebukawa, B. De Gregorio, L. Bonal, L. Remusat, R. Stroud, E. Quirico, L. Nittler, M. Hashiguchi, M. Komatsu, T. Okumura, J. Mathurin, E. Dartois, J. Duprat, Y. Takahashi, Y. Takeichi, D. Kilcoyne, S. Yamashita, A. Dazzi, A. Deniset-Besseau, S. Sandford, Z. Martins, Y. Tamenori, T. Ohigashi, H. Suga, D. Wakabayashi, M. Verdier-Paoletti, S. Mostefaoui, G. Montagnac, J. Barosch, K. Kamide, M. Shigenaka, L. Bejach, M. Matsumoto, Y. Enokido, T. Noguchi, H. Yurimoto, T. Nakamura, R. Okazaki, H. Naraoka, K. Sakamoto, H. C. Connolly, D. S. Lauretta, M. Abe, T. Okada, T. Yada, M. Nishimura, K. Yogata, A. Nakato, M. Yoshitake, A. Iwamae, S. Furuya, K. Hatakeda, A. Miyazaki, H. Soejima, Y. Hitomi, K. Kumagai, T. Usui, T. Hayashi, D. Yamamoto, R. Fukai, S. Sugita, K. Kitazato, N. Hirata, R. Honda, T. Morota, E. Tatsumi, N. Sakatani, N. Namiki, K. Matsumoto, R. Noguchi, K. Wada, H. Senshu, K. Ogawa, Y. Yokota, Y. Ishihara, Y. Shimaki, M. Yamada, C. Honda, T. Michikami, M. Matsuoka, N. Hirata, M. Arakawa, C. Okamoto, M. Ishiguro, R. Jaumann, J.-P. Bibring, M. Grott, S. Schröder, K. Otto, C. Pilorget, N. Schmitz, J. Biele, T.-M. Ho, A. Moussi-Soffys, A. Miura, H. Noda, T. Yamada, K. Yoshihara, K. Kawahara, H. Ikeda, Y. Yamamoto, K. Shirai, S. Kikuchi, N. Ogawa, H. Takeuchi, G. Ono, Y. Mimasu, K. Yoshikawa, Y. Takei, A. Fujii, Y. Iijima, S. Nakazawa, S. Hosoda, T. Iwata, M. Hayakawa, H. Sawada, H. Yano, R. Tsukizaki, M. Ozaki, F. Terui, S. Tanaka, M. Fujimoto, M. Yoshikawa, T. Saiki, S. Tachibana, S. Watanabe, Y. Tsuda, Macromolecular organic matter in samples of the asteroid (162173) Ryugu. *Science* **379** (2023).

53. T. D. Glotch, G. R. Rossman, O. Aharonson, Mid-infrared (5–100 μm) reflectance spectra and optical constants of ten phyllosilicate minerals. *Icarus.* **192**, 605–622 (2007).

54. C. Lantz, R. Brunetto, M. A. Barucci, S. Fornasier, D. Baklouti, J. Bourçois, M. Godard, Ion irradiation of carbonaceous chondrites: A new view of space weathering on primitive asteroids. *Icarus.* **285**, 43–57 (2017).

55. M. Endreß, A. Bischoff, Carbonates in CI chondrites: Clues to parent body evolution. *Geochim. Cosmochim. Acta* **60**, 489–507 (1996).

56. M. Gounelle, M. E. Zolensky, A terrestrial origin for sulfate veins in CI1 chondrites. *Meteorit. Planet. Sci.* **36**, 1321–1329 (2001).

57. A. J. King, K. J. H. Phillips, S. Strekopytov, C. Vita-Finzi, S. S. Russell, Terrestrial modification of the Ivuna meteorite and a reassessment of the chemical composition of the CI type specimen. *Geochim. Cosmochim. Acta* **268**, 73–89 (2020).

58. M. Noun, D. Baklouti, R. Brunetto, F. Borondics, T. Calligaro, Z. Dionnet, L. L. S. D'Hendecourt, B. Nsouli, I. Ribaud, M. Roumie, S. Della Negra, A mineralogical context for the organic matter in the paris meteorite determined by a multi-technique analysis. *Life (Basel),* **9**, 44 (2019).

59. J. W. Salisbury, D. M. D'Aria, E. Jarosewich, Midinfrared (2.5–13.5 μm) reflectance spectra of powdered stony meteorites. *Icarus.* **92**, 280–297 (1991).

60. E. A. Cloutis, V. B. Pietrasz, C. Kiddell, M. R. M. Izawa, P. Vernazza, T. H. Burbine, F. DeMeo, K. T. Tait, J. F. Bell, P. Mann, D. M. Applin, V. Reddy, Spectral reflectance "deconstruction" of the Murchison CM2 carbonaceous chondrite and implications for spectroscopic investigations of dark asteroids. *Icarus.* **305**, 203–224 (2018).

61. M. E. Zolensky, K. Nakamura, M. Gounelle, E. Tonui, T. Mikouchi, O. Tochikawa, T. Kasama, Mineralogy of Tagish Lake, a unique type 2 carbonaceous chondrite. *Meteorit. Planet. Sci.* **37**, 737–761 (2002).

62. N. Noda, S. Yamashita, Y. Takahashi, M. Matsumoto, Y. Enokido, K. Amano, T. Kawai, H. Sakuma, K. Fukushi, Y. Sekine, T. Nakamura, Anaerobic microscopic analysis of ferrous saponite and its sensitivity to oxidation by earth's air: Lessons learned for analysis of returned samples from mars and carbonaceous asteroids. *Minerals*, **11**, 1244 (2021).

63. K. Mogi, S. Yamashita, T. Nakamura, M. Matsuoka, S. Okumura, Y. Furukawa, "Dehydration process of experimentally heated Murchison without any effects of adsorbed and rehydrated water," paper presented at 80th Annual Meeting of the Meteoritical Society, Santa Fe, NM, 23 to 28, July 2017.

64. S. Rubino, S. Potin, C. Lantz, D. Baklouti, P. Beck, O. Brissaud, H. Leroux, E. Quirico, B. Schmitt, F. Borondics, R. Brunetto, Geometry induced bias in the remote near-IR identification of phyllosilicates on space weathered bodies. *Icarus.* **376**, 114887 (2022).

65. P. A. Bland, G. Cressey, O. N. Menzies, Modal mineralogy of carbonaceous chondrites by X-ray diffraction and Mössbauer spectroscopy. *Meteorit. Planet. Sci.* **39**, 3–16 (2004).

66. A. J. King, P. F. Schofield, K. T. Howard, S. S. Russell, Modal mineralogy of CI and CI-like chondrites by X-ray diffraction. *Geochim. Cosmochim. Acta* **165**, 148–160 (2015).

67. P. Beck, A. Maturilli, A. Garenne, P. Vernazza, J. Helbert, E. Quirico, B. Schmitt, What is controlling the reflectance spectra (0.35–150 μm) of hydrated (and dehydrated) carbonaceous chondrites? *Icarus.* **313**, 124–138 (2018).

68. M. R. M. Izawa, E. A. Cloutis, T. Rhind, S. A. Mertzman, D. M. Applin, J. M. Stromberg, D. M. Sherman, Spectral reflectance properties of magnetites: Implications for remote sensing. *Icarus.* **319**, 525–539 (2019).

69. R. Sultana, O. Poch, P. Beck, B. Schmitt, E. Quirico, S. Spadaccia, L. Patty, A. Pommerol, A. Maturilli, J. Helbert, G. Alemanno, Reflection, emission, and polarization properties of surfaces made of hyperfine grains, and implications for the nature of primitive small bodies. *Icarus.* **395**, 115492 (2023).

70. R. Brunetto, C. Lantz, Y. Fukuda, A. Aléon-Toppani, T. Nakamura, Z. Dionnet, D. Baklouti, F. Borondics, Z. Djouadi, S. Rubino, K. Amano, M. Matsumoto, Y. Fujioka, T. Morita, M. Kukuiri, E. Kagawa, M. Matsuoka, R. Milliken, H. Yurimoto, T. Noguchi, R. Okazaki, H. Yabuta, H. Naraoka, K.

Sakamoto, S. Tachibana, T. Yada, M. Nishimura, A. Nakato, A. Miyazaki, K. Yogata, M. Abe, T. Okada, T. Usui, M. Yoshikawa, T. Saiki, S. Tanaka, F. Terui, S. Nakazawa, S. Watanabe, Y. Tsuda, Ryugu's anhydrous ingredients and their spectral link to primitive dust from the outer solar system. *Astrophys J Lett.* **951**, L33 (2023).

71. T. LePivert-Jolivet, R. Brunetto, C. Pilorget, J.-P. Bibring, A. Nakato, D. Loizeau, L. Riu, V. Hamm, C. Lantz, K. Hatakeda, K. Yogata, D. Baklouti, T. Yada, T. Okada, T. Usui, The MicrOmega ISAS Curation Team, "Grain scale heterogeneities in Ryugu samples as observed by MicrOmega: a key to understand aqueous alteration and space weathering," paper presented at 85th Annual Meeting of the Meteoritical Society, Glasgow, Scotland, 14 to 19 August 2022.

72. M. Matsuoka, T. Nakamura, Y. Kimura, T. Hiroi, R. Nakamura, S. Okumura, S. Sasaki, Pulse-laser irradiation experiments of Murchison CM2 chondrite for reproducing space weathering on C-type asteroids. *Icarus.* **254**, 135–143 (2015).

73. M. Zolensky, R. Barrett, L. Browning, Mineralogy and composition of matrix and chondrule rims in carbonaceous chondrites. *Geochim. Cosmochim. Acta* **57**, 3123–3148 (1993).

74. L. Baker, I. A. Franchi, I. P. Wright, C. T. Pillinger, The oxygen isotopic composition of water from Tagish Lake: Its relationship to low-temperature phases and to other carbonaceous chondrites. *Meteorit. Planet. Sci.* **37**, 977–985 (2002).

75. K. Tomeoka, P. R. Buseck, Matrix mineralogy of the Orgueil CI carbonaceous chondrite. *Geochim. Cosmochim. Acta* **52**, 1627–1640 (1988).

76. M. J. Loeffler, B. S. Prince, A possible explanation for the blue spectral slope observed on B-type asteroids. *Icarus.* **376**, 114881 (2022).

77. S. M. Chemtob, R. D. Nickerson, R. V. Morris, D. G. Agresti, J. G. Catalano, Oxidative alteration of ferrous smectites and implications for the redox evolution of early Mars. *J Geophys Res Planets.* **122**, 2469–2488 (2017).

78. S. Tardivel, P. Sánchez, D. J. Scheeres, Equatorial cavities on asteroids, an evidence of fission events. *Icarus.* **304**, 192–208 (2018).

79. Y. Shimaki, H. Senshu, N. Sakatani, T. Okada, T. Fukuhara, S. Tanaka, M. Taguchi, T. Arai, H. Demura, Y. Ogawa, K. Suko, T. Sekiguchi, T. Kouyama, S. Hasegawa, J. Takita, T. Matsunaga, T. Imamura, T. Wada, K. Kitazato, N. Hirata, N. Hirata, R. Noguchi, S. Sugita, S. Kikuchi, T. Yamaguchi, N. Ogawa, G. Ono, Y. Mimasu, K. Yoshikawa, T. Takahashi, Y. Takei, A. Fujii, H. Takeuchi, Y. Yamamoto, M. Yamada, K. Shirai, Y. Iijima, K. Ogawa, S. Nakazawa, F. Terui, T. Saiki, M. Yoshikawa, Y. Tsuda, S. Watanabe, Thermophysical properties of the surface of asteroid 162173 Ryugu: Infrared observations and thermal inertia mapping. *Icarus.* **348** (2020).

80. F. E. DeMeo, R. P. Binzel, S. M. Slivan, S. J. Bus, An extension of the Bus asteroid taxonomy into the near-infrared. *Icarus.* **202**, 160–180 (2009).
